# Supplementary material for: A system-level model for the microbial regulatory genome
Source: Mol Syst Biol. 2014 Jul 15;10(7):740. doi: 10.15252/msb.20145160 (PMC4299497; doi:10.15252/msb.20145160)
Supplement: Supplementary file 1 — Supplementary Dataset S1 [file msb0010-0740-sd1.doc]

**Supplementary Dataset S1, Related to Figure 2. *H. salinarum* GREs discovered in EGRIN 2.0.**

Column descriptions:

1. GRE index
2. Number of cis-regulatory motifs in the GRE motif cluster
3. Location opf peak in distribution of locations of GRE relative to translation start sites of genes (as plotted in Supplementary Figure 3B)
4. Motif logo of GRE

| **GRE** | **N CRMs** | **Location** | **Logo** |
| --- | --- | --- | --- |
| 1 | 1486 | 103.5 | 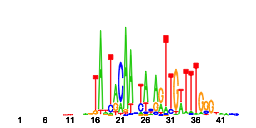 |
| 2 | 1352 | 127.1 | 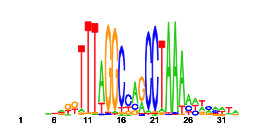 |
| 5 | 777 | 86.0 | 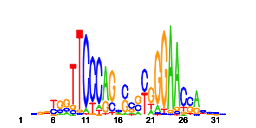 |
| 6 | 776 | 81.8 | 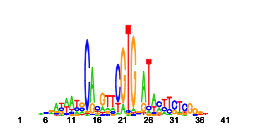 |
| 7 | 761 | 59.4 | 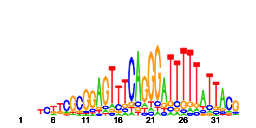 |
| 9 | 529 | 108.0 | 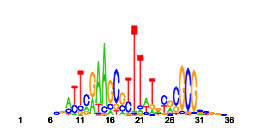 |
| 10 | 489 | 113.1 | 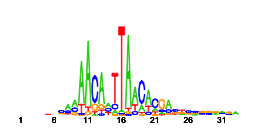 |
| 11 | 485 | 97.5 | 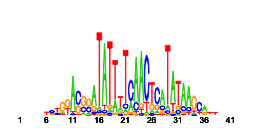 |
| 13 | 447 | 30.3 | 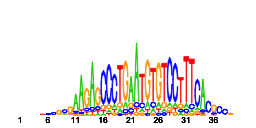 |
| 14 | 438 | 83.5 | 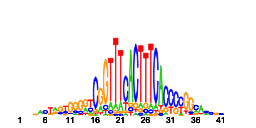 |
| 15 | 418 | 101.4 | 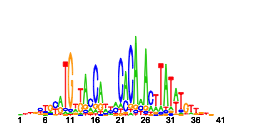 |
| 16 | 393 | 109.7 | 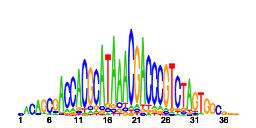 |
| 17 | 390 | 119.8 | 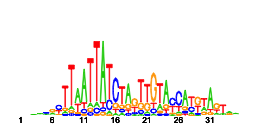 |
| 19 | 372 | 96.0 | 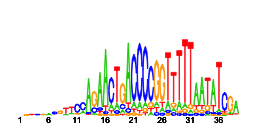 |
| 21 | 362 | 118.5 | 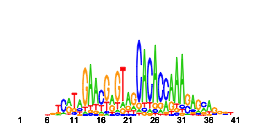 |
| 22 | 360 | 97.2 | 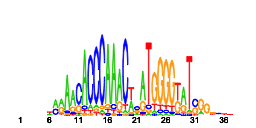 |
| 23 | 339 | 99.4 | 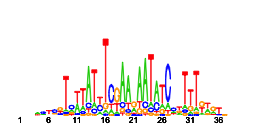 |
| 25 | 333 | 110.3 | 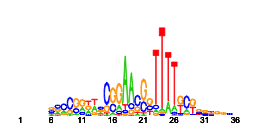 |
| 27 | 309 | 99.8 | 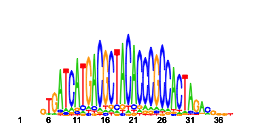 |
| 28 | 301 | 108.4 | 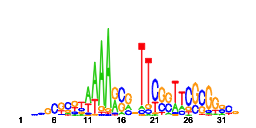 |
| 29 | 293 | 7.1 | 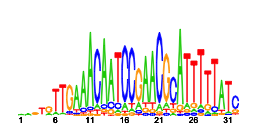 |
| 30 | 273 | 116.3 | 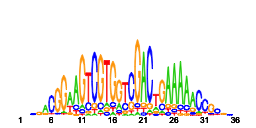 |
| 32 | 252 | 106.9 | 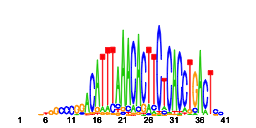 |
| 33 | 246 | 108.2 | 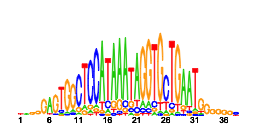 |
| 35 | 232 | 110.3 | 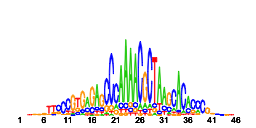 |
| 37 | 222 | 43.2 | 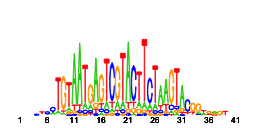 |
| 40 | 217 | 81.9 | 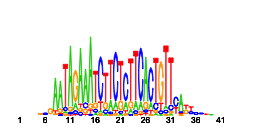 |
| 42 | 209 | 114.3 | 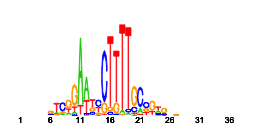 |
| 45 | 202 | 112.9 | 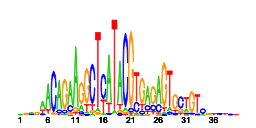 |
| 46 | 198 | 124.2 | 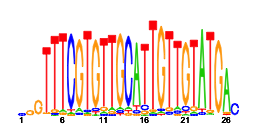 |
| 49 | 186 | 85.9 | 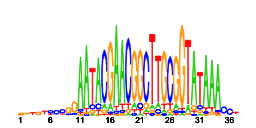 |
| 50 | 182 | 107.0 | 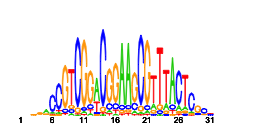 |
| 53 | 177 | 121.1 | 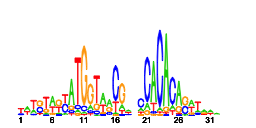 |
| 54 | 175 | 114.4 | 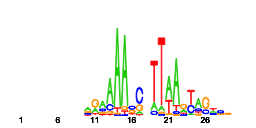 |
| 57 | 169 | 94.4 | 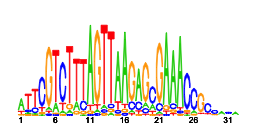 |
| 61 | 165 | 106.7 | 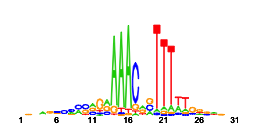 |
| 64 | 158 | 51.7 | 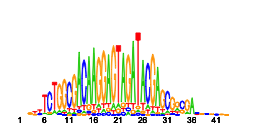 |
| 66 | 150 | 61.2 | 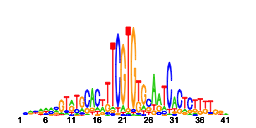 |
| 68 | 143 | 72.2 | 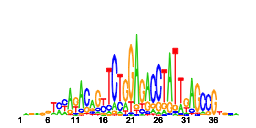 |
| 69 | 143 | 113.2 | 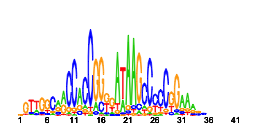 |
| 72 | 138 | 119.6 | 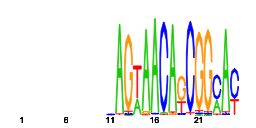 |
| 74 | 127 | 104.4 | 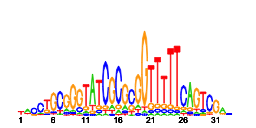 |
| 75 | 124 | 104.0 | 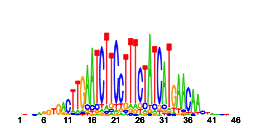 |
| 77 | 121 | 83.2 | 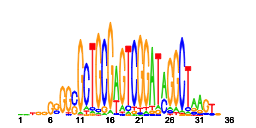 |
| 78 | 120 | 53.0 | 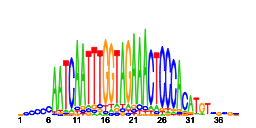 |
| 81 | 115 | 124.5 | 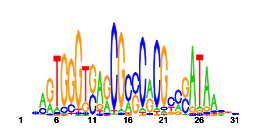 |
| 83 | 113 | 120.6 | 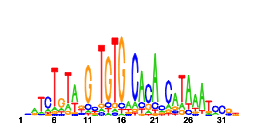 |
| 85 | 111 | 122.3 | 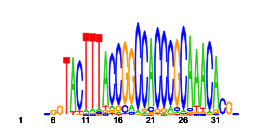 |
| 87 | 109 | 103.4 | 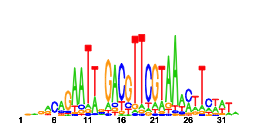 |
| 88 | 106 | 72.1 | 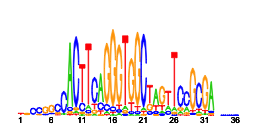 |
| 89 | 103 | 72.1 | 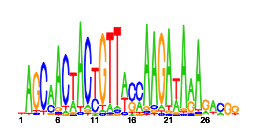 |
| 90 | 103 | 123.2 | 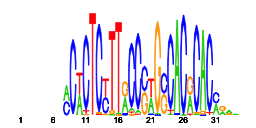 |
| 92 | 102 | 111.1 | 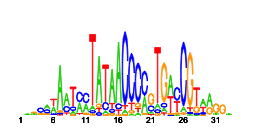 |
| 93 | 100 | 113.5 | 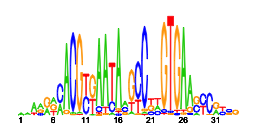 |
| 94 | 99 | 59.5 | 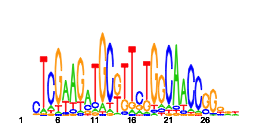 |
| 95 | 98 | 96.6 | 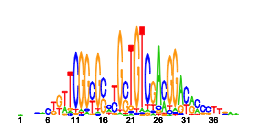 |
| 96 | 98 | 104.8 | 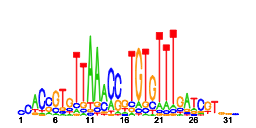 |
| 98 | 97 | 109.0 | 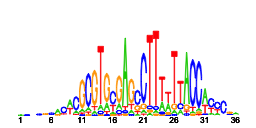 |
| 100 | 96 | 10.5 | 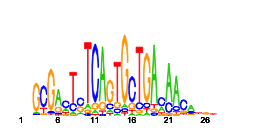 |
| 102 | 95 | 106.8 | 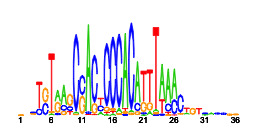 |
| 103 | 94 | 103.0 | 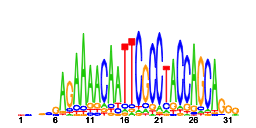 |
| 105 | 93 | 117.7 | 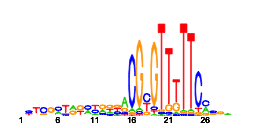 |
| 106 | 93 | 14.5 | 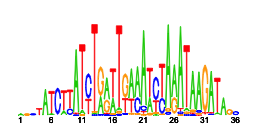 |
| 107 | 92 | 124.7 | 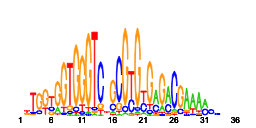 |
| 108 | 92 | 114.3 | 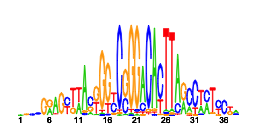 |
| 111 | 90 | 102.8 | 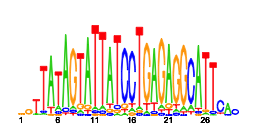 |
| 114 | 89 | 16.7 | 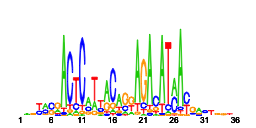 |
| 116 | 88 | 110.3 | 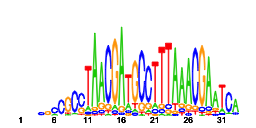 |
| 117 | 88 | 36.8 | 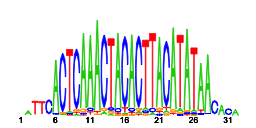 |
| 118 | 87 | 107.2 | 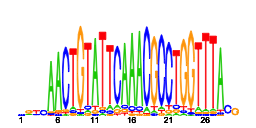 |
| 120 | 85 | 35.6 | 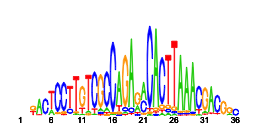 |
| 122 | 84 | 117.0 | 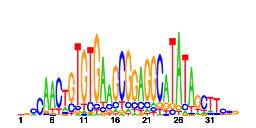 |
| 128 | 79 | 34.2 | 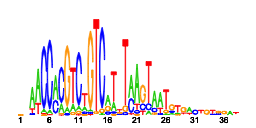 |
| 130 | 78 | 66.3 | 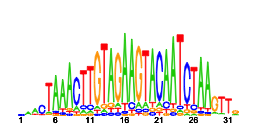 |
| 131 | 77 | 108.2 | 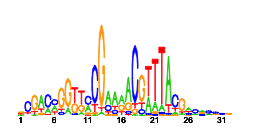 |
| 133 | 77 | 123.8 | 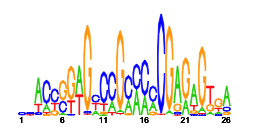 |
| 134 | 77 | 140.3 | 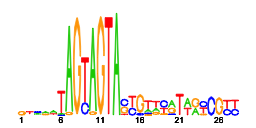 |
| 136 | 76 | 58.0 | 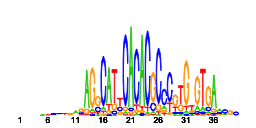 |
| 138 | 75 | 84.6 | 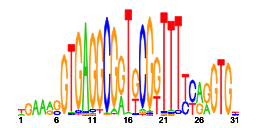 |
| 140 | 73 | 126.3 | 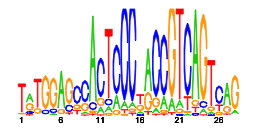 |
| 143 | 70 | 113.2 | 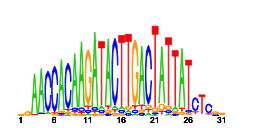 |
| 148 | 64 | 117.1 | 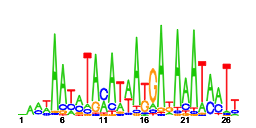 |
| 150 | 63 | 9.2 | 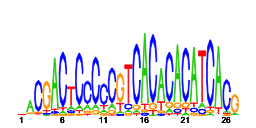 |
| 153 | 62 | 62.5 | 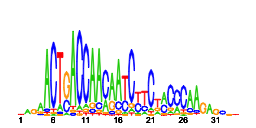 |
| 154 | 61 | 109.9 | 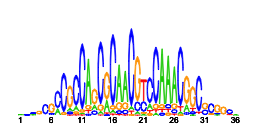 |
| 160 | 59 | 125.5 | 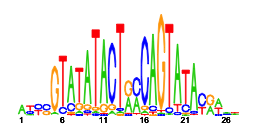 |
| 164 | 59 | 113.6 | 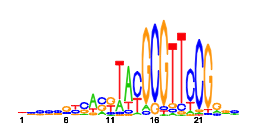 |
| 165 | 58 | 110.7 | 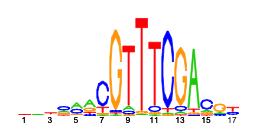 |
| 166 | 58 | 122.2 | 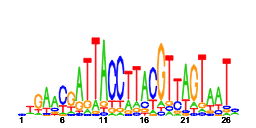 |
| 170 | 57 | 122.6 | 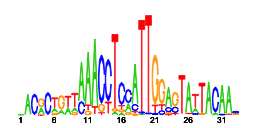 |
| 171 | 57 | 57.3 | 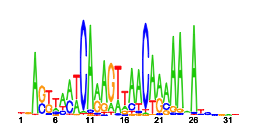 |
| 186 | 51 | 114.3 | 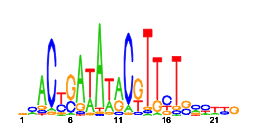 |
| 187 | 50 | 108.8 | 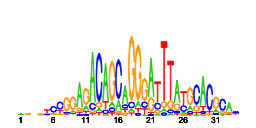 |
| 188 | 50 | 101.2 | 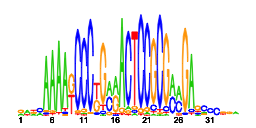 |
| 189 | 50 | 107.3 | 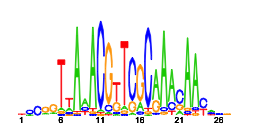 |
| 190 | 50 | 47.5 | 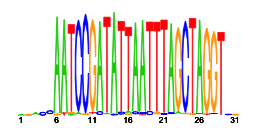 |
| 196 | 47 | 15.3 | 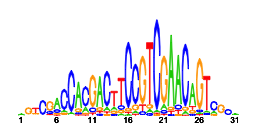 |
| 201 | 46 | 58.2 | 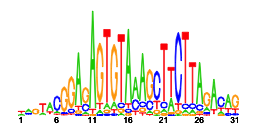 |
| 205 | 45 | 33.7 | 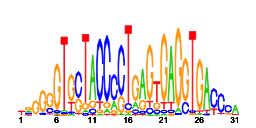 |
| 207 | 45 | 118.5 | 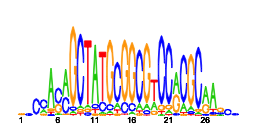 |
| 210 | 44 | 111.0 |  |
| 212 | 44 | 54.2 |  |
| 219 | 43 | 89.5 |  |
| 221 | 42 | 84.1 |  |
| 222 | 42 | 73.9 |  |
| 226 | 41 | 107.8 |  |
| 227 | 40 | 102.4 |  |
| 229 | 40 | 87.9 |  |
| 233 | 39 | 15.6 |  |
| 238 | 38 | 23.7 |  |
| 247 | 36 | 29.3 |  |
| 249 | 36 | 109.9 |  |
| 250 | 35 | 110.0 |  |
| 254 | 35 | 69.2 |  |
| 255 | 35 | 138.1 |  |
| 256 | 34 | 13.2 |  |
| 260 | 34 | 13.5 |  |
| 261 | 34 | 109.8 |  |
| 264 | 34 | 114.4 |  |
| 266 | 34 | 84.6 |  |
| 267 | 34 | 126.0 |  |
| 270 | 33 | 111.0 |  |
| 271 | 33 | 59.9 |  |
| 274 | 33 | 103.6 |  |
| 275 | 33 | 45.0 |  |
| 276 | 33 | 80.1 |  |
| 277 | 33 | 79.7 |  |
| 278 | 32 | 114.0 |  |
| 283 | 31 | 93.5 |  |
| 287 | 31 | 100.8 |  |
| 291 | 31 | 119.2 |  |
| 296 | 30 | 104.1 |  |
| 298 | 30 | 103.2 |  |
| 299 | 30 | 80.2 |  |
| 300 | 30 | 59.5 |  |
