# Supplementary material for: A system-level model for the microbial regulatory genome
Source: Mol Syst Biol. 2014 Jul 15;10(7):740. doi: 10.15252/msb.20145160 (PMC4299497; doi:10.15252/msb.20145160)
Supplement: Supplementary file 2 — Supplementary Dataset S2 [file msb0010-0740-sd2.doc]

**Supplementary Dataset S2, Related to Figure 2. *E. coli* GREs discovered in EGRIN 2.0.**

Column descriptions:

1. GRE index
2. Number of cis-regulatory motifs in the GRE motif cluster
3. Location of peak in distribution of locations of GRE relative to translation start sites of genes (as plotted in Supplementary Figure 3B)
4. Best transcription factor match from experimentally mapped TFBS in RegulonDB
5. FDR q-value for significance of transcription factor match in column (4)
6. Fraction of cis-regulatory motifs in the GRE motif cluster with significant matches to the transcription factor listed in column (4)
7. Motif logo of GRE

| **GRE** | **N.motifs** | **Location** | **RDB.Match** | **RDB.Match.Qval** | **RDB.Match.Fraction** | **Logo** |
| --- | --- | --- | --- | --- | --- | --- |
| 1 | 1502 | 20.7 |  | NA | NA | 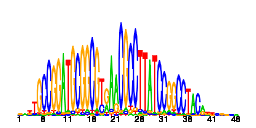 |
| 2 | 435 | 74.9 |  | NA | NA | 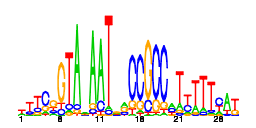 |
| 3 | 420 | 75.2 | Fur | 0 | 0.97 | 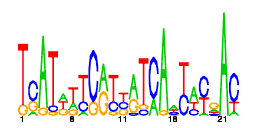 |
| 4 | 331 | 57.9 | PurR | 0 | 0.99 | 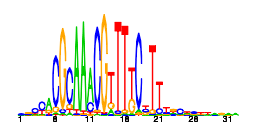 |
| 5 | 320 | 51.7 | ArcA | 2.6e-111 | 0.62 | 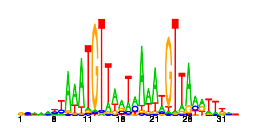 |
| 6 | 317 | 72.5 |  | NA | NA | 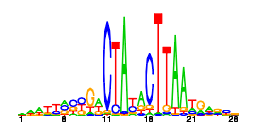 |
| 7 | 285 | 102.0 |  | NA | NA | 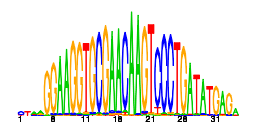 |
| 8 | 258 | 24.4 | CytR | 1.9e-57 | 0.28 | 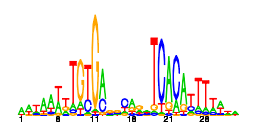 |
| 9 | 250 | 84.3 |  | NA | NA | 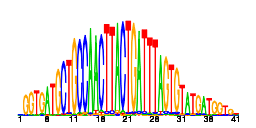 |
| 10 | 213 | 41.3 |  | NA | NA | 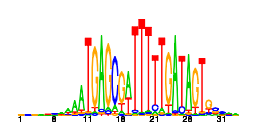 |
| 12 | 210 | 69.6 | ArgR | 0 | 1 | 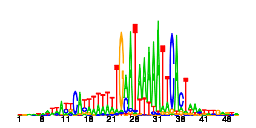 |
| 13 | 184 | 83.3 |  | NA | NA | 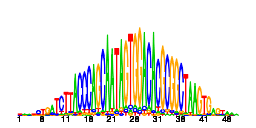 |
| 14 | 174 | 111.1 |  | NA | NA | 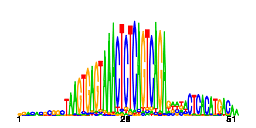 |
| 15 | 152 | 45.6 |  | NA | NA | 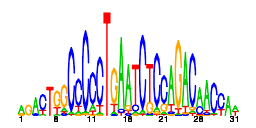 |
| 16 | 146 | 20.0 | GadE | 0 | 0.95 | 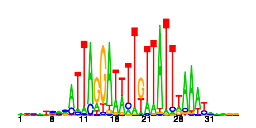 |
| 17 | 143 | 115.7 |  | NA | NA | 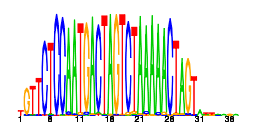 |
| 18 | 142 | 40.7 | FNR | 1.9e-100 | 0.87 | 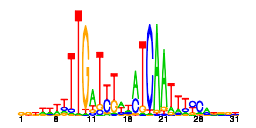 |
| 19 | 120 | 53.5 | MetJ | 4.2e-252 | 0.97 | 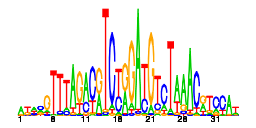 |
| 20 | 102 | 18.7 |  | NA | NA | 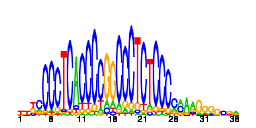 |
| 21 | 98 | 54.5 | FlhDC | 1.8e-99 | 0.8 | 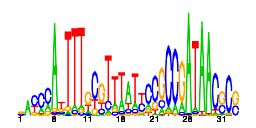 |
| 22 | 92 | 95.9 |  | NA | NA | 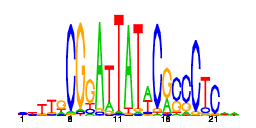 |
| 23 | 85 | 46.9 |  | NA | NA | 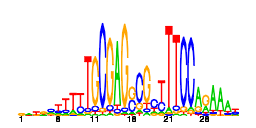 |
| 24 | 83 | 20.8 |  | NA | NA | 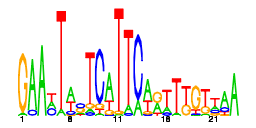 |
| 25 | 81 | 51.6 | MelR | 1.7e-07 | 0.11 | 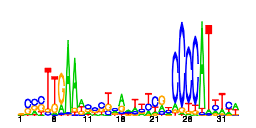 |
| 26 | 77 | 71.1 |  | NA | NA | 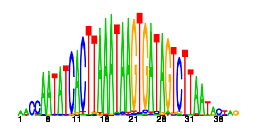 |
| 27 | 74 | 111.4 |  | NA | NA | 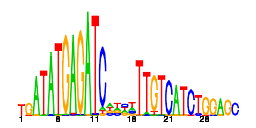 |
| 28 | 73 | 66.1 |  | NA | NA | 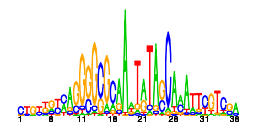 |
| 29 | 71 | 68.4 |  | NA | NA | 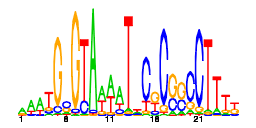 |
| 30 | 69 | 71.8 | IclR | 1.4e-09 | 0.17 | 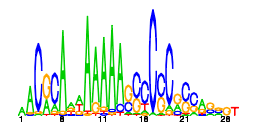 |
| 31 | 67 | 71.3 | NarP | 1.3e-21 | 0.34 | 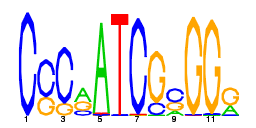 |
| 32 | 61 | 84.4 |  | NA | NA | 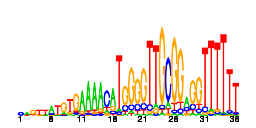 |
| 33 | 60 | 18.2 |  | NA | NA | 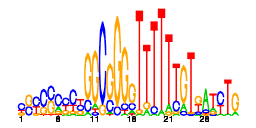 |
| 34 | 57 | 46.3 | MalT | 6.5e-102 | 0.98 | 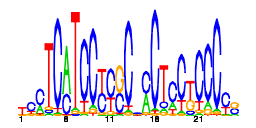 |
| 35 | 54 | 20.4 | GadW | 8.1e-74 | 0.87 | 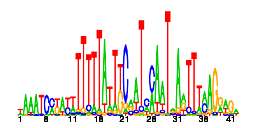 |
| 36 | 53 | 37.2 |  | NA | NA | 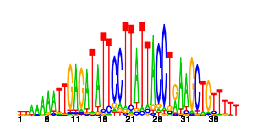 |
| 37 | 51 | 71.7 |  | NA | NA | 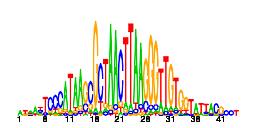 |
| 38 | 51 | 33.7 | PhoB | 0.003 | 0.18 | 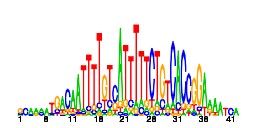 |
| 39 | 50 | 89.7 |  | NA | NA | 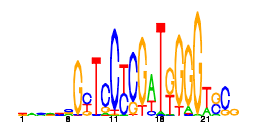 |
| 40 | 48 | 79.7 |  | NA | NA | 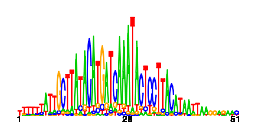 |
| 41 | 47 | 73.2 |  | NA | NA | 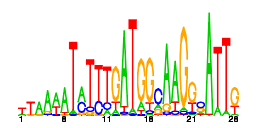 |
| 42 | 45 | 82.8 | LexA | 1.1e-60 | 0.98 | 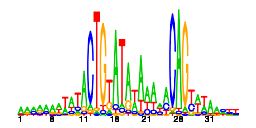 |
| 43 | 45 | 91.9 |  | NA | NA | 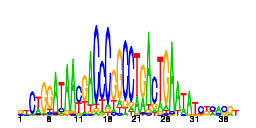 |
| 44 | 45 | 19.9 | FruR | 9.2e-71 | 0.93 | 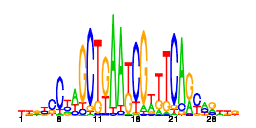 |
| 45 | 45 | 15.0 | GntR | 4.4e-08 | 0.22 | 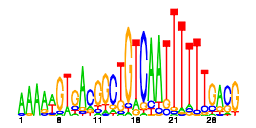 |
| 47 | 43 | 24.0 |  | NA | NA | 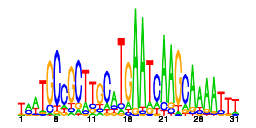 |
| 48 | 43 | 13.5 |  | NA | NA | 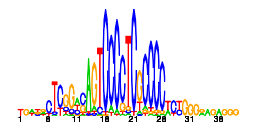 |
| 49 | 43 | 12.8 | UlaR | 0.0022 | 0.12 | 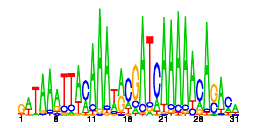 |
| 50 | 43 | 22.3 | CysB | 1.6e-06 | 0.28 | 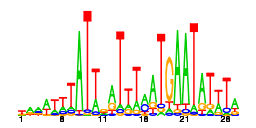 |
| 51 | 41 | 73.4 | RcsB | 1.8e-54 | 0.88 | 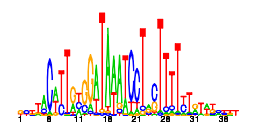 |
| 52 | 40 | 42.0 |  | NA | NA | 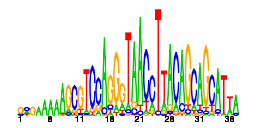 |
| 54 | 39 | 41.0 |  | NA | NA | 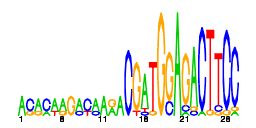 |
| 55 | 39 | 70.3 |  | NA | NA | 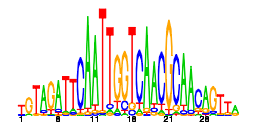 |
| 56 | 39 | 58.2 | CynR | 2.4e-05 | 0.13 | 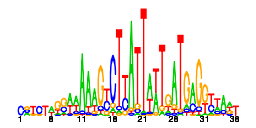 |
| 57 | 38 | 12.8 | NrdR | 1e-05 | 0.13 | 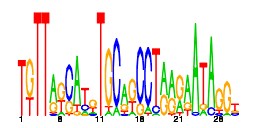 |
| 58 | 38 | 65.8 | CueR | 9.5e-81 | 0.92 | 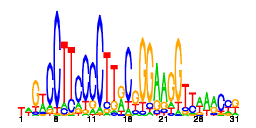 |
| 59 | 37 | 37.1 |  | NA | NA | 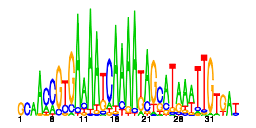 |
| 60 | 36 | 71.0 |  | NA | NA | 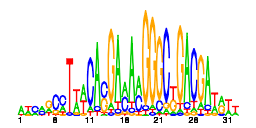 |
| 61 | 36 | 76.5 |  | NA | NA | 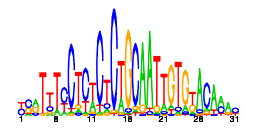 |
| 63 | 35 | 30.6 | FlhDC | 3.9e-28 | 0.71 | 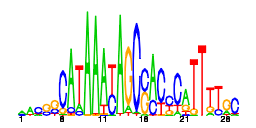 |
| 64 | 35 | 32.8 |  | NA | NA | 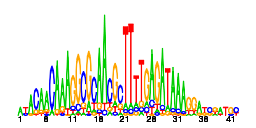 |
| 65 | 35 | 77.3 |  | NA | NA | 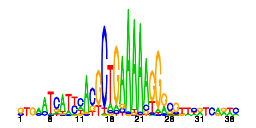 |
| 66 | 34 | 16.3 | FlhDC | 8.4e-27 | 0.71 | 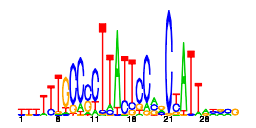 |
| 67 | 34 | 32.7 | RhaS | 5.3e-06 | 0.15 | 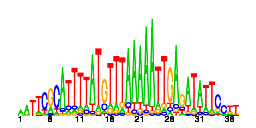 |
| 68 | 33 | 18.4 |  | NA | NA | 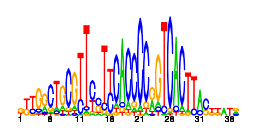 |
| 69 | 32 | 45.7 |  | NA | NA | 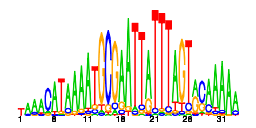 |
| 71 | 31 | 78.6 | OxyR | 3.5e-08 | 0.42 | 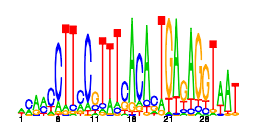 |
| 73 | 31 | 38.9 |  | NA | NA | 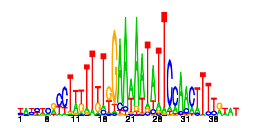 |
| 74 | 31 | 15.2 |  | NA | NA | 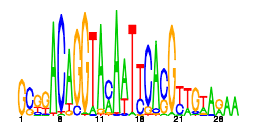 |
| 75 | 31 | 104.7 |  | NA | NA | 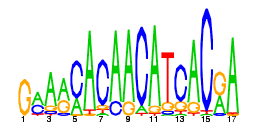 |
| 76 | 30 | 101.1 |  | NA | NA | 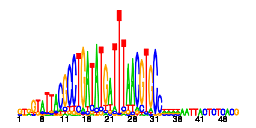 |
| 77 | 30 | 43.4 |  | NA | NA | 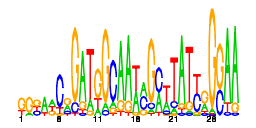 |
| 78 | 30 | 70.0 |  | NA | NA | 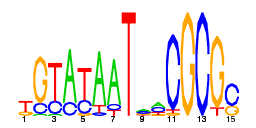 |
| 79 | 30 | 14.3 |  | NA | NA | 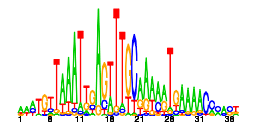 |
| 80 | 30 | 58.7 |  | NA | NA | 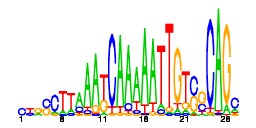 |
| 81 | 29 | 29.0 |  | NA | NA | 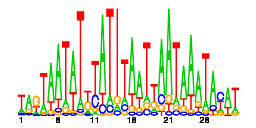 |
| 82 | 29 | 16.4 |  | NA | NA | 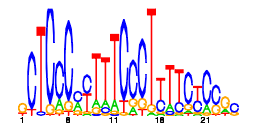 |
| 83 | 29 | 26.5 | UlaR | 0.0043 | 0.14 | 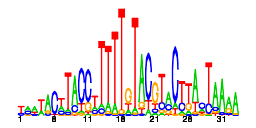 |
| 84 | 29 | 22.8 |  | NA | NA | 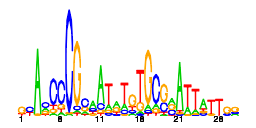 |
| 85 | 29 | 26.7 |  | NA | NA | 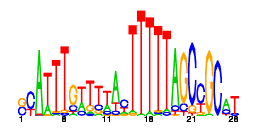 |
| 87 | 28 | 23.2 | MetJ | 0.0033 | 0.18 | 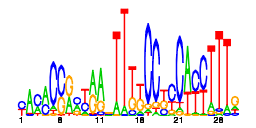 |
| 88 | 27 | 9.2 |  | NA | NA | 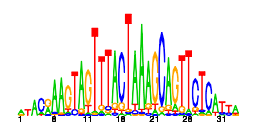 |
| 89 | 27 | 47.0 |  | NA | NA | 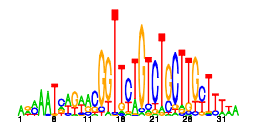 |
| 90 | 27 | 69.8 |  | NA | NA | 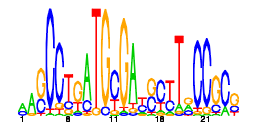 |
| 91 | 27 | 45.3 |  | NA | NA | 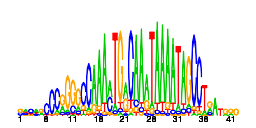 |
| 92 | 27 | 82.9 |  | NA | NA | 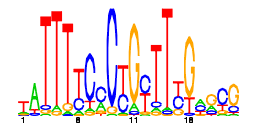 |
| 93 | 27 | 62.9 |  | NA | NA | 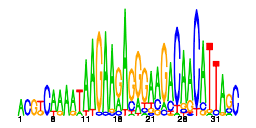 |
| 94 | 27 | 45.6 |  | NA | NA | 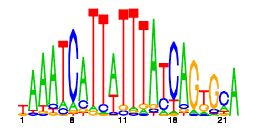 |
| 95 | 26 | 58.5 | CysB | 2e-27 | 0.85 | 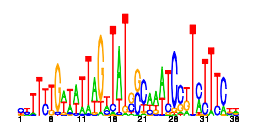 |
| 96 | 26 | 81.5 |  | NA | NA | 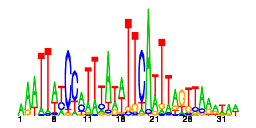 |
| 97 | 26 | 46.9 |  | NA | NA | 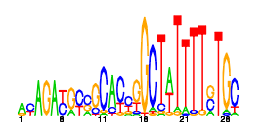 |
| 98 | 25 | 35.6 |  | NA | NA | 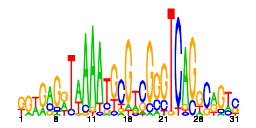 |
| 99 | 25 | 24.7 |  | NA | NA | 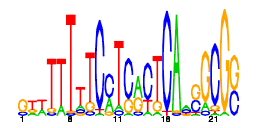 |
| 100 | 25 | 58.9 |  | NA | NA | 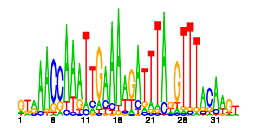 |
| 101 | 24 | 13.4 |  | NA | NA | 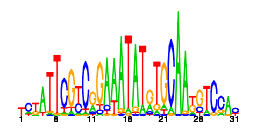 |
| 102 | 24 | 14.4 | MalT | 5.1e-06 | 0.29 | 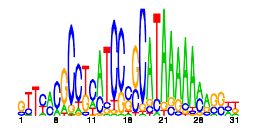 |
| 103 | 24 | 93.2 | FlhDC | 5.4e-30 | 0.92 | 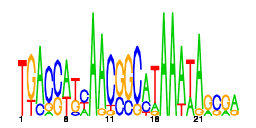 |
| 104 | 24 | 43.8 |  | NA | NA | 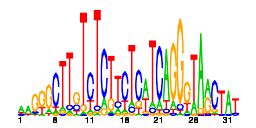 |
| 105 | 24 | 90.0 | Zur | 0.00049 | 0.17 | 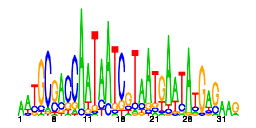 |
| 106 | 24 | 76.5 |  | NA | NA | 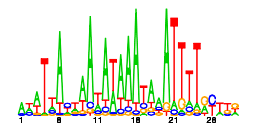 |
| 107 | 24 | 69.7 |  | NA | NA | 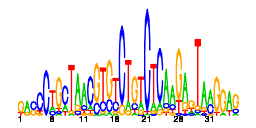 |
| 108 | 24 | 76.1 | NarL | 7.6e-11 | 0.62 |  |
| 109 | 24 | 31.8 |  | NA | NA |  |
| 110 | 23 | 9.2 |  | NA | NA |  |
| 113 | 23 | 25.7 |  | NA | NA |  |
| 114 | 23 | 47.6 |  | NA | NA |  |
| 115 | 23 | 25.8 |  | NA | NA |  |
| 116 | 23 | 60.4 | MngR | 1.4e-18 | 0.52 |  |
| 117 | 23 | 93.3 |  | NA | NA |  |
| 118 | 23 | 17.5 |  | NA | NA |  |
| 119 | 22 | 105.1 | ArgR | 7.6e-27 | 0.95 |  |
| 120 | 21 | 16.9 | AsnC | 0.016 | 0.14 |  |
| 121 | 21 | 17.7 |  | NA | NA |  |
| 122 | 21 | 72.8 |  | NA | NA |  |
| 123 | 21 | 51.1 |  | NA | NA |  |
| 124 | 21 | 25.8 | OxyR | 0.013 | 0.29 |  |
| 125 | 21 | 16.3 |  | NA | NA |  |
| 126 | 21 | 80.8 |  | NA | NA |  |
| 127 | 21 | 68.6 |  | NA | NA |  |
| 128 | 21 | 84.7 |  | NA | NA |  |
| 129 | 21 | 51.1 |  | NA | NA |  |
| 130 | 21 | 29.0 |  | NA | NA |  |
| 131 | 20 | 49.8 | CysB | 1.9e-26 | 0.95 |  |
| 133 | 20 | 25.5 |  | NA | NA |  |
| 134 | 20 | 48.7 | CytR | 7.1e-06 | 0.35 |  |
| 135 | 20 | 40.6 |  | NA | NA |  |
| 136 | 20 | 84.9 |  | NA | NA |  |
| 137 | 20 | 44.2 |  | NA | NA |  |
| 139 | 19 | 89.9 |  | NA | NA |  |
| 140 | 19 | 55.9 |  | NA | NA |  |
| 141 | 19 | 70.6 |  | NA | NA |  |
| 142 | 19 | 16.9 |  | NA | NA |  |
| 143 | 19 | 19.1 |  | NA | NA |  |
| 144 | 19 | 19.3 |  | NA | NA |  |
| 145 | 19 | 46.8 |  | NA | NA |  |
| 146 | 19 | 30.6 | RutR | 4.7e-35 | 0.89 |  |
| 147 | 19 | 74.4 |  | NA | NA |  |
| 148 | 19 | 14.4 |  | NA | NA |  |
| 149 | 19 | 40.7 |  | NA | NA |  |
| 151 | 18 | 57.0 |  | NA | NA |  |
| 152 | 18 | 44.6 | GadW | 0.0096 | 0.22 |  |
| 153 | 18 | 9.3 |  | NA | NA |  |
| 154 | 18 | 65.8 |  | NA | NA |  |
| 156 | 18 | 43.2 |  | NA | NA |  |
| 157 | 18 | 15.5 |  | NA | NA |  |
| 158 | 18 | 56.2 |  | NA | NA |  |
| 159 | 18 | 73.8 |  | NA | NA |  |
| 160 | 18 | 52.0 |  | NA | NA |  |
| 161 | 18 | 70.5 |  | NA | NA |  |
| 162 | 18 | 20.6 |  | NA | NA |  |
| 163 | 18 | 148.1 | TrpR | 2.3e-22 | 0.67 |  |
| 164 | 18 | 34.6 |  | NA | NA |  |
| 165 | 18 | 13.9 |  | NA | NA |  |
| 166 | 18 | 21.1 |  | NA | NA |  |
| 167 | 17 | 13.2 | FlhDC | 1.5e-17 | 0.82 |  |
| 168 | 17 | 79.4 |  | NA | NA |  |
| 169 | 17 | 98.8 | IscR | 0.0072 | 0.24 |  |
| 171 | 17 | 94.5 |  | NA | NA |  |
| 172 | 17 | 79.7 | UlaR | 6.2e-18 | 0.65 |  |
| 173 | 17 | 33.7 |  | NA | NA |  |
| 174 | 17 | 56.3 |  | NA | NA |  |
| 175 | 17 | 44.7 |  | NA | NA |  |
| 176 | 16 | 70.4 |  | NA | NA |  |
| 177 | 16 | 68.8 |  | NA | NA |  |
| 178 | 16 | 22.9 | FlhDC | 0 | 1 |  |
| 179 | 16 | 63.6 | FruR | 0.0039 | 0.25 |  |
| 180 | 16 | 86.0 |  | NA | NA |  |
| 181 | 16 | 98.0 |  | NA | NA |  |
| 182 | 16 | 30.2 |  | NA | NA |  |
| 183 | 16 | 73.5 |  | NA | NA |  |
| 184 | 16 | 45.9 |  | NA | NA |  |
| 185 | 16 | 73.9 | IscR | 1.1e-21 | 0.88 |  |
| 186 | 16 | 37.8 |  | NA | NA |  |
| 187 | 16 | 26.6 |  | NA | NA |  |
| 188 | 16 | 10.3 |  | NA | NA |  |
| 189 | 16 | 67.2 | CysB | 0.0049 | 0.31 |  |
| 190 | 16 | 79.3 |  | NA | NA |  |
| 191 | 16 | 34.5 | NsrR | 0.0054 | 0.38 |  |
| 192 | 16 | 61.8 |  | NA | NA |  |
| 193 | 16 | 75.7 |  | NA | NA |  |
| 194 | 16 | 12.9 | IdnR | 0.0073 | 0.12 |  |
| 195 | 16 | 95.0 |  | NA | NA |  |
| 196 | 15 | 62.9 |  | NA | NA |  |
| 197 | 15 | 77.9 |  | NA | NA |  |
| 198 | 15 | 32.8 |  | NA | NA |  |
| 199 | 15 | 71.3 |  | NA | NA |  |
| 200 | 15 | 18.0 | AraC | 1.7e-05 | 0.33 |  |
| 201 | 15 | 56.0 |  | NA | NA |  |
| 202 | 15 | 54.6 | MprA | 5.6e-33 | 0.87 |  |
| 203 | 15 | 13.4 | EvgA | 0.0053 | 0.13 |  |
| 204 | 15 | 16.2 | NarL | 4.8e-05 | 0.53 |  |
| 205 | 15 | 73.9 |  | NA | NA |  |
| 206 | 15 | 89.6 |  | NA | NA |  |
| 207 | 15 | 81.5 |  | NA | NA |  |
| 208 | 14 | 69.0 | CysB | 2.6e-15 | 0.86 |  |
| 209 | 14 | 60.1 |  | NA | NA |  |
| 210 | 14 | 21.2 |  | NA | NA |  |
| 211 | 14 | 52.2 |  | NA | NA |  |
| 212 | 14 | 20.6 |  | NA | NA |  |
| 213 | 14 | 102.6 |  | NA | NA |  |
| 214 | 14 | 59.7 |  | NA | NA |  |
| 215 | 14 | 19.5 | FhlA | 0 | 1 |  |
| 216 | 14 | 22.2 | H-NS | 0.014 | 0.36 |  |
| 217 | 14 | 25.6 |  | NA | NA |  |
| 218 | 14 | 38.1 |  | NA | NA |  |
| 219 | 14 | 84.0 |  | NA | NA |  |
| 220 | 14 | 59.7 |  | NA | NA |  |
| 221 | 14 | 21.3 |  | NA | NA |  |
| 222 | 14 | 13.4 |  | NA | NA |  |
| 223 | 14 | 31.3 |  | NA | NA |  |
| 224 | 14 | 86.1 |  | NA | NA |  |
| 225 | 14 | 39.8 |  | NA | NA |  |
| 226 | 14 | 89.0 |  | NA | NA |  |
| 227 | 14 | 90.4 |  | NA | NA |  |
| 228 | 14 | 79.1 |  | NA | NA |  |
| 229 | 14 | 36.3 |  | NA | NA |  |
| 230 | 13 | 98.4 |  | NA | NA |  |
| 232 | 13 | 13.4 |  | NA | NA |  |
| 233 | 13 | 55.3 |  | NA | NA |  |
| 234 | 13 | 63.7 |  | NA | NA |  |
| 236 | 13 | 57.0 |  | NA | NA |  |
| 237 | 13 | 40.9 |  | NA | NA |  |
| 238 | 13 | 53.2 |  | NA | NA |  |
| 239 | 13 | 33.9 |  | NA | NA |  |
| 240 | 13 | 109.7 |  | NA | NA |  |
| 241 | 13 | 15.4 |  | NA | NA |  |
| 242 | 13 | 86.2 |  | NA | NA |  |
| 243 | 13 | 44.4 |  | NA | NA |  |
| 244 | 13 | 73.6 |  | NA | NA |  |
| 245 | 13 | 72.1 |  | NA | NA |  |
| 246 | 13 | 75.5 | Zur | 2.1e-09 | 0.46 |  |
| 247 | 13 | 66.3 |  | NA | NA |  |
| 248 | 13 | 72.4 |  | NA | NA |  |
| 249 | 13 | 41.4 |  | NA | NA |  |
| 250 | 13 | 77.1 |  | NA | NA |  |
| 251 | 13 | 48.1 | AscG | 0.011 | 0.15 |  |
| 252 | 13 | 17.6 |  | NA | NA |  |
| 253 | 13 | 42.8 |  | NA | NA |  |
| 254 | 13 | 81.3 |  | NA | NA |  |
| 255 | 13 | 27.4 | RutR | 1.9e-05 | 0.31 |  |
| 257 | 13 | 62.9 |  | NA | NA |  |
| 259 | 12 | 55.2 |  | NA | NA |  |
| 261 | 12 | 65.3 | OxyR | 0.038 | 0.33 |  |
| 262 | 12 | 116.4 | DeoR | 5.8e-07 | 0.33 |  |
| 263 | 12 | 66.2 |  | NA | NA |  |
| 264 | 12 | 54.7 |  | NA | NA |  |
| 265 | 12 | 20.1 | RcsB | 0.0018 | 0.33 |  |
| 266 | 12 | 93.5 |  | NA | NA |  |
| 267 | 12 | 84.4 |  | NA | NA |  |
| 268 | 12 | 95.9 |  | NA | NA |  |
| 269 | 12 | 14.5 | FlhDC | 1.9e-05 | 0.5 |  |
| 270 | 12 | 100.1 |  | NA | NA |  |
| 271 | 12 | 67.7 |  | NA | NA |  |
| 272 | 12 | 79.9 | IdnR | 0.0032 | 0.17 |  |
| 273 | 12 | 13.8 |  | NA | NA |  |
| 274 | 12 | 87.6 | PhoB | 0.0094 | 0.33 |  |
| 275 | 12 | 67.2 |  | NA | NA |  |
| 276 | 12 | 67.6 |  | NA | NA |  |
| 277 | 12 | 51.4 |  | NA | NA |  |
| 278 | 12 | 59.1 |  | NA | NA |  |
| 279 | 12 | 73.7 |  | NA | NA |  |
| 280 | 12 | 14.0 | MalT | 0 | 1 |  |
| 281 | 12 | 71.0 |  | NA | NA |  |
| 283 | 12 | 20.4 | OxyR | 3.3e-11 | 0.83 |  |
| 284 | 12 | 58.3 |  | NA | NA |  |
| 285 | 12 | 35.0 |  | NA | NA |  |
| 286 | 12 | 97.3 | EvgA | 2.3e-05 | 0.25 |  |
| 287 | 12 | 88.7 |  | NA | NA |  |
| 288 | 12 | 74.7 |  | NA | NA |  |
| 289 | 12 | 68.4 |  | NA | NA |  |
| 291 | 11 | 35.3 | Lrp | 0 | 1 |  |
| 292 | 11 | 25.5 |  | NA | NA |  |
| 293 | 11 | 23.4 |  | NA | NA |  |
| 294 | 11 | 61.0 | PurR | 4.4e-07 | 0.64 |  |
| 295 | 11 | 81.7 |  | NA | NA |  |
| 296 | 11 | 79.5 |  | NA | NA |  |
| 297 | 11 | 36.4 |  | NA | NA |  |
| 298 | 11 | 53.3 |  | NA | NA |  |
| 299 | 11 | 99.0 |  | NA | NA |  |
| 300 | 11 | 98.2 | SoxS | 3e-06 | 0.64 |  |
| 301 | 11 | 16.5 | Fur | 8.4e-11 | 0.91 |  |
| 302 | 11 | 52.9 |  | NA | NA |  |
| 303 | 11 | 44.0 |  | NA | NA |  |
| 304 | 11 | 31.2 |  | NA | NA |  |
| 305 | 11 | 18.3 | UlaR | 5.7e-14 | 0.73 |  |
| 306 | 11 | 31.6 |  | NA | NA |  |
| 307 | 11 | 59.1 | TorR | 1.1e-10 | 0.73 |  |
| 308 | 11 | 34.3 |  | NA | NA |  |
| 309 | 11 | 30.9 | Zur | 0.021 | 0.18 |  |
| 310 | 11 | 97.3 |  | NA | NA |  |
| 311 | 11 | 10.3 |  | NA | NA |  |
| 312 | 11 | 33.3 |  | NA | NA |  |
| 313 | 11 | 70.9 |  | NA | NA |  |
| 314 | 11 | 57.9 |  | NA | NA |  |
| 315 | 11 | 73.0 |  | NA | NA |  |
| 316 | 11 | 61.1 |  | NA | NA |  |
| 317 | 11 | 83.6 |  | NA | NA |  |
| 318 | 11 | 67.1 |  | NA | NA |  |
| 319 | 11 | 19.4 |  | NA | NA |  |
| 320 | 11 | 31.6 |  | NA | NA |  |
| 321 | 11 | 95.4 | Zur | 0 | 1 |  |
| 322 | 11 | 58.7 |  | NA | NA |  |
| 323 | 11 | 75.7 |  | NA | NA |  |
| 324 | 11 | 75.2 |  | NA | NA |  |
| 325 | 11 | 75.3 |  | NA | NA |  |
| 326 | 11 | 124.0 | GadW | 1.9e-05 | 0.45 |  |
| 327 | 11 | 103.3 |  | NA | NA |  |
| 328 | 11 | 38.7 | NarL | 0 | 1 |  |
| 329 | 11 | 47.7 | NarL | 1.5e-08 | 0.82 |  |
| 330 | 10 | 25.1 |  | NA | NA |  |
| 331 | 10 | 56.4 | RcsB | 0.016 | 0.3 |  |
| 333 | 10 | 82.5 |  | NA | NA |  |
| 334 | 10 | 95.5 |  | NA | NA |  |
| 335 | 10 | 138.3 |  | NA | NA |  |
| 336 | 10 | 79.3 |  | NA | NA |  |
| 337 | 10 | 41.1 |  | NA | NA |  |
| 338 | 10 | 32.5 |  | NA | NA |  |
| 340 | 10 | 19.8 |  | NA | NA |  |
| 341 | 10 | 38.7 |  | NA | NA |  |
| 342 | 10 | 81.7 |  | NA | NA |  |
| 343 | 10 | 68.6 |  | NA | NA |  |
| 345 | 10 | 16.0 | FNR | 0.00027 | 0.7 |  |
| 346 | 10 | 53.1 |  | NA | NA |  |
| 347 | 10 | 59.7 |  | NA | NA |  |
| 348 | 10 | 37.1 |  | NA | NA |  |
| 349 | 10 | 14.8 |  | NA | NA |  |
| 350 | 10 | 28.0 | GlpR | 8.1e-10 | 0.7 |  |
| 351 | 10 | 70.6 |  | NA | NA |  |
| 352 | 10 | 55.2 |  | NA | NA |  |
| 353 | 10 | 25.7 |  | NA | NA |  |
| 354 | 10 | 97.6 |  | NA | NA |  |
| 355 | 10 | 52.0 |  | NA | NA |  |
| 356 | 10 | 84.7 | AsnC | 0.03 | 0.2 |  |
| 357 | 10 | 14.4 | MurR | 5.7e-17 | 0.8 |  |
| 358 | 10 | 69.4 |  | NA | NA |  |
| 359 | 10 | 81.5 |  | NA | NA |  |
| 360 | 10 | 88.4 |  | NA | NA |  |
| 361 | 10 | 24.7 |  | NA | NA |  |
| 362 | 10 | 69.2 |  | NA | NA |  |
| 363 | 10 | 70.7 |  | NA | NA |  |
| 364 | 10 | 60.8 |  | NA | NA |  |
| 365 | 10 | 68.5 |  | NA | NA |  |
| 369 | 10 | 46.9 | CynR | 6.9e-21 | 0.9 |  |
| 370 | 10 | 23.8 |  | NA | NA |  |
| 371 | 10 | 107.5 |  | NA | NA |  |
| 372 | 10 | 50.4 |  | NA | NA |  |
| 373 | 10 | 13.2 | OxyR | 0.016 | 0.4 |  |
| 374 | 10 | 68.0 |  | NA | NA |  |
| 375 | 10 | 98.9 |  | NA | NA |  |
| 376 | 10 | 16.8 | NadR | 2e-06 | 0.3 |  |
| 377 | 10 | 126.6 |  | NA | NA |  |
| 378 | 10 | 43.1 | XylR | 0.013 | 0.2 |  |
| 379 | 10 | 101.9 |  | NA | NA |  |
| 380 | 10 | 17.9 | DeoR | 0.0034 | 0.2 |  |
| 381 | 10 | 41.0 |  | NA | NA |  |
| 382 | 10 | 35.8 | MarA | 2.2e-08 | 0.8 |  |
| 383 | 10 | 56.0 |  | NA | NA |  |
| 384 | 10 | 72.9 |  | NA | NA |  |
| 385 | 10 | 14.8 | UlaR | 1.5e-07 | 0.5 |  |
| 387 | 10 | 25.5 |  | NA | NA |  |
| 388 | 10 | 74.2 |  | NA | NA |  |
| 389 | 10 | 66.6 |  | NA | NA |  |
| 390 | 10 | 32.5 | NsrR | 0.031 | 0.4 |  |
| 391 | 10 | 49.7 |  | NA | NA |  |
| 392 | 10 | 63.8 |  | NA | NA |  |
| 393 | 10 | 38.6 |  | NA | NA |  |
| 394 | 10 | 20.8 |  | NA | NA |  |
| 397 | 10 | 105.9 |  | NA | NA |  |
| 398 | 10 | 106.0 |  | NA | NA |  |
| 400 | 10 | 64.1 |  | NA | NA |  |
| 401 | 10 | 75.1 |  | NA | NA |  |
| 402 | 10 | 103.3 |  | NA | NA |  |
