# Supplementary material for: A system-level model for the microbial regulatory genome
Source: Mol Syst Biol. 2014 Jul 15;10(7):740. doi: 10.15252/msb.20145160 (PMC4299497; doi:10.15252/msb.20145160)
Supplement: Supplementary file 10 — Supplementary Information [file msb0010-0740-sd10.pdf]

Supplementary Information

Aaron N. Brooks[1,2,*], David J. Reiss[1,*,†], Antoine Allard[3], Wei-Ju Wu[1], Diego M. Salvanha[4], Christopher L. Plaisier[1], Sriram Chandrasekaran[1], Min Pan[1], Amardeep Kaur[1] and Nitin S. Baliga[1,2,5,6,†]

[1]Institute for Systems Biology, 1441 N 34th Street, Seattle, WA 98103
[2]Molecular and Cellular Biology Program, University of Washington, Seattle, WA 98195
[3]Dpartement de Physique, de Gnie Physique et d'Optique, Universit Laval, Qubec, QC, Canada
[4]LabPIB, Department of Computing and Mathematics FFCLRP-USP, University of Sao Paulo, Ribeirao Preto, Brazil
[5]Departments of Microbiology and Biology, University of Washington, Seattle, WA 98195
[6]Lawrence Berkeley National Laboratories, Berkeley, CA 94720
[*]Equal contribution
[†]To whom correspondence should be addressed:
nbaliga@systemsbiology.org; dreiss@systemsbiology.org

June 6, 2014

Contents

1 Online materials **5**

2 Experimental data used for model construction **5**
2.1 mRNA expression data 5
2.1.1 *H. salinarium NRC-1* compendium 5
2.1.2 *E. coli K-12 MG1655* expression compendia 6
2.1.2.1 Use of the DISTILLER data compendium for model training 6
2.1.2.2 Use of the DREAM5 data compendium for model validation 6
2.2 Additional data integrated for model construction 7
2.2.1 Genome sequence data and annotatations for cMonkey analysis 7
2.2.2 Operon membership predictions used for cMonkey analysis 7
2.2.3 Predicted transcriptional regulators used for Inferelator analysis 7
2.2.3.1 *H. salinarium NRC-1* 7
2.2.3.2 *E. coli K-12 MG1655* 7
2.2.4 Functional association networks integrated into cMonkey analysis 7

3 Independent data used for model validation **8**
3.1 *H. salinarium NRC-1* 8
3.1.1 Tiling array transcriptome measurements 8
3.1.2 ChIP-chip transcription factor binding measurements for global regulators 8
3.1.3 *kdp* promoter serial truncation measurements 8
3.2 *E. coli K-12 MG1655* 8
3.2.1 Tiling array transcriptome measurements 8
3.2.2 PurR/ΔPurR expression data and ChIP-chip transcription factor binding sites 8
3.2.3 Fitness measurements 9

3.2.4 Effector molecule measurements 9
3.2.5 Experimentally mapped *E. coli* transcription factor binding sites 9
3.2.6 Experimentally measured *E. coli* transcription factor regulatory targets 9

4 Computational methods **9**
4.1 cMonkey: integrated biclustering algorithm, updated for ensemble analysis 9
4.1.1 Introduction and summary 9
4.1.2 Updates since original publication 10
4.1.3 Detailed cMonkey algorithm description 10
4.1.4 Parameter ranges used for EGRIN 2.0 11
4.1.5 cMonkey software availability 12
4.2 Inferelator: inference of transcriptional regulatory influences 13
4.2.1 Introduction and summary 13
4.2.2 Updates since original publication 13
4.2.3 Detailed algorithm description 13
4.3 EGRIN 2.0 model construction 14
4.3.1 Background and motivation 14
4.3.2 "Ensemble of EGRINs": generation and statistical mining 15
4.3.3 Clustering of cis-regulatory motifs to identify GREs 17
4.3.4 Genome-wide scanning of motifs to obtain GRE locations 18
4.3.5 Identifying corems 19
4.3.5.1 Gene-gene co-occurrence network 19
4.3.5.2 Network backbone extraction 19
4.3.5.3 Network link-community detection 20
4.4 Functional enrichment estimates for genes in corems 20
4.5 Conditional co-regulation of genes organized in corems 21
4.6 Conditionality of GRE influence 22
4.7 Detection of conditional operons 22
4.8 Environmental ontology construction and usage 22

5 Model validation **24**
5.1 Global validation of gene regulatory elements predicted by EGRIN 2.0 24
5.2 Global validation of regulatory interactions predicted by EGRIN 2.0 24
5.2.1 Conversion of EGRIN 2.0 Inferelator influence predictions into a GRN 25
5.2.2 Conversion of EGRIN 2.0 GRE detections into a predicted GRN 25
5.2.3 Integration of predicted EGRIN 2.0 Inferelator- and GRE-based GRNs 25
5.2.4 Network comparisons and global performance assessments 26
5.3 Validation of condition-specific operon isoforms by tiling array transcriptome measurements 27
5.4 Gene-gene co-fitness correlations in regulatory modules 28

6 Model evaluation **32**
6.1 Comparison with other module detection algorithms 32
6.2 Convergence and stability of the inferred network 32
6.3 Discovery of corems in an independent data set 33

7 Additional Supporting Figures Referenced From Main Text **34**

List of Figures

S1 Detailed workflow for EGRIN 2.0 inference procedure . . . 16
S2 Motif clustering and GRE identification . . . 18
S3 Genome-wide distribution of GREs relative to experimentally mapped transcriptional start sites in *H. salinarum* . . . 19
S4 Corem density as a function of clustering cutoff threshold . . . 21
S5 Corem statistics . . . 21
S6 Deciphering GREs responsible for regulating corems . . . 23
S7 Environmental ontology hierarchically organizes relationships between experimental conditions from metadata collected across 1495 experiments in *H. salinarum* . . . 24
S8 Precision-recall performance for *E. coli* networks. . . . 26
S9 Ensemble performance of individual GRN predictions . . . 27
S10 Integration of GRE discovery and Inferelator predictions yields comprehensive and detailed gene regulatory networks . . . 29
S11 GREs regulate multiple transcript isoforms from operons in *E. coli*, *dppABCDF* . . . 29
S12 GREs regulate multiple transcript isoforms from operons in *E. coli*, *galETKM* . . . 30
S13 GREs regulate multiple transcript isoforms from operons in *E. coli*, *ptsH-ptsI-crr* . . . 30
S14 EGRIN 2.0 models highly correlated co-fitness relationships that cannot be explained by operons or regulons . . . 31
S15 Number of TFs in RegulonDB re-discovered by various regulatory module detection methods. . . 32
S16 Convergence of EGRIN 2.0 gene co-occurrence networks. . . . 33
S17 Reproducibility of corems across data sets . . . 34
S18 Differential GRE activity in *pyrL* promoter, *E. coli* . . . 35
S19 GREs regulate multiple transcript isoforms from operons in *H. salinarum*, *nirH-VNG1775C-hemA*. . . 35
S20 GREs regulate multiple transcript isoforms from operons in *H. salinarum*, *sdhCDBA*. . . . 36
S21 GREs regulate multiple transcript isoforms from operons in *H. salinarum*, *VNG2211H-endA-trpS1*. 36
S22 Alternate regulatory modes for *dpp* operon predicted by corems . . . 37
S23 Network representation of transcriptional isoforms for the *dpp* operon predicted by corems . . . 38
S24 *dppA* is more tightly co-expressed with genes of hc6326 in some environments than the other genes in the *dpp* operon . . . 38
S25 Evidence for condition-specific transcript isoforms of the *dpp* operon in *E. coli* . . . 39
S26 Corems model the mechanistic basis for conditional subdivision of the PurR regulon, *E. coli* . . . 39
S27 Corems integrate diverse regulatory mechanisms, *E. coli* . . . 40
S28 Genes from corems related to nucleotide biosynthesis have highly similar fitness effects when they are deleted . . . 40
S29 Corems model fitness effects that occur in specific environments . . . 41
S30 Metabolite correlations may explain co-regulation within metabolically-linked corems . . . 41

List of Tables

S1 cMonkey parameters used for the *H. salinarium NRC-1* ensemble. 12
S2 cMonkey parameters used for the *E. coli K-12 MG1655* ensemble. 12
S3 Global properties of *H. salinarum* and *E. coli* ensembles 17

Abstract

Microbes can tailor transcriptional responses to diverse environmental challenges despite having streamlined genomes and a limited number of regulators. Here, we present data-driven models that capture the dynamic interplay of the environment and genome-encoded regulatory programs of two types of prokaryotes: E. coli (a bacterium) and H. salinarum (an archaeon). The models reveal how the genome-wide distributions of cis-acting gene regulatory elements and the conditional influences of transcription factors at each of those elements encode programs for eliciting a wide array of environment-specific responses. We demonstrate how these programs partition transcriptional regulation of genes within regulons and operons to re-organize gene-gene functional associations in each environment. The models capture fitness-relevant co-regulation by different transcriptional control mechanisms acting across the entire genome, to define a generalized, system-level organizing principle for prokaryotic gene regulatory networks that goes well beyond existing paradigms of gene regulation.

1 Online materials

Additional figures, tables, supporting data, and comprehensive model predictions are available at: `http://egrin2.systemsbiology.net`.

2 Experimental data used for model construction

2.1 mRNA expression data

2.1.1 *H. salinarum NRC-1* compendium

A compendium of 1495 transcriptome profiles were collated from a wide array of experiments conducted by our lab over the past decade that cover dynamic transcriptional responses to varied growth (1159 arrays), nutritional (161 arrays), and stress conditions (1102 arrays), including variation in temperature (256 arrays), oxygen (285 arrays), light (786 arrays), salinity (20 arrays), metal ions (274 arrays), and genetic perturbations (643 arrays). We categorized the experiments using extensive metadata collected at the time of the experiment. We used this metadata to construct a GO-like ontology of the relationships between all experiments (discussed in detail below). The annotation counts above are derived from this resource (note that a single array can receive more than one annotation). A full list of the metadata, annotations, and ontology is available on the web service. 1159 of the arrays are published ([5, 4, 9, 20, 21, 31, 32, 52, 53, 54, 62, 63]. 336 of the arrays are new for this study. Experimental protocols are identical to [9]. These data, including expression levels ($\log_2$ ratios vs. reference samples) and experimental metadata, are available online as a tab-delimited spreadsheet.

Each array in the *H. salinarum* compendium was collected using the same platform, using the same reference, and processed and normalized using the same protocol. More specifically, each RNA sample was hybridized along with a *H. salinarum NRC-1* reference RNA prepared under standard conditions (mid-logarithmic phase batch cultures grown at 37°C in CM, OD = 0.5). Samples were hybridized to a 70-mer oligonucleotide array containing the 2400 non-redundant open reading frames (ORFs) of the *H. salinarum NRC-1* genome as described in [5]. Each ORF was spotted on each array in quadruplicate and dye flipping was conducted (to rule out bias in dye incorporation) for all samples, yielding eight technical replicates per gene per sample. At least two independent biological replicates exist for all experimental conditions for a total of 16 replicates per gene per condition. Direct RNA labeling, slide hybridization, and washing protocols were performed as described by [22, 53]. Raw intensity signals from each slide were processed by the SBEAMS-microarray pipeline [42] (www.SBEAMS.org/microarray), in which the data were median normalized and subjected to significant analysis of microarrays (SAM) and variability and error estimates analysis (VERA). Each data point was assigned a significance statistic, λ, using maximum likelihood [26].

2.1.2 *E. coli K-12 MG1655* expression compendia

2.1.2.1 Use of the DISTILLER data compendium for model training

A total of 868 *E. coli K-12 MG1655* transcriptome profiles were compiled by [39] for use with their DISTILLER algorithm. These data were collated from publicly available microarray databases: 44 arrays from Stanford Microarray Database [18], 617 from Gene Expression Omnibus [7] and 36 from ArrayExpress [47], as well as 181 arrays from supplementary data in literature (for four different experiments). The experiments cover a range of conditions, including varying carbon sources (136 arrays), pH (46 arrays), oxygen (284 arrays), metals (27 arrays) and temperature (23 arrays). Overall, the compendium consists of measurements from single channel (407 arrays; including 298 Affymetrix, and 109 P33) and dual channel (460 arrays; including 337 DNA/cDNA and 126 oligonucleotide) platforms.

These microarray measurements were normalized by the authors [39], as follows: "If possible, raw intensities were preferred as data source over normalized data provided by the public repository. Dual-channel data were loess fitted to remove nonlinear, dye-related discrepancies. No background correction procedures were performed to avoid an increase in expression logratio variance for lower, less reliable intensity levels. Whenever raw data were available, single-channel data were first normalized per experiment with RMA. Logratios were then created for the single-channel data in order to combine them with the dual channel measurements. For each single-channel array, expression logratios were computed by comparing the normalized values against an artificial reference array. This artificial reference array was constructed on a per experiment basis by taking the median expression of each gene across all arrays in the corresponding experiment. When deemed necessary (e.g. experiments normalized by MAS5.0 for which the raw data was not available), a loess fit was performed on these logratios. To ensure that the artificial reference was not altered by this intensity dependent non-linear rescaling, the artificial reference expression levels were chosen for the average log intensity (instead of the mean expression levels of the respective array and the artificial reference). To ensure comparability between arrays with a different reference, gene expression profiles were median centered across arrays that share the same reference. An additional variance rescaling of the gene expression profiles was performed to render genes with differing magnitudes of expression changes more comparable."

The authors further note that, "the array composition of the modules generated by DISTILLER is not biased towards arrays from any specific platform, indicating a correct preprocessing of the microarray compendium." [39] It is for this reason that we chose this normalized *E. coli* microarray compendium for EGRIN 2.0 analysis.

2.1.2.2 Use of the DREAM5 data compendium for model validation

To ascertain the generalizability of EGRIN 2.0 models across data sets, we inferred a second *E. coli* EGRIN 2.0 model on an independent *E. coli* gene expression compendium. By comparing this model to the original model we inferred using the DISTILLER data set, we were able (1) to understand what, if any, systematic biases exist due to normalization procedures, and (2) to cross-validate EGRIN 2.0 predictions across two data set. Detailed discussion of the results from this analysis are provided in Section 5.

We obtained the de-anonymized *E. coli* microarray compendium from the DREAM5 competition website [41]. According to the authors, these data were "compiled for *E. coli*, where all chips are the same Affymetrix platform, the *E. coli* Antisense Genome Array. Chips were downloaded from GEO (Platform ID: GPL199). In total, 805 chips with available raw data Affymetrix files (.CEL files) were compiled." Additionally, "Microarray normalization was done using Robust Multichip Averaging (RMA) 9 through the software RMAExpress. All 160 chips were uploaded into RMAExpress and normalization was done as one batch. All arrays were background adjusted, quantile normalized, and probesets were summarized using median polish. Normalized data was exported as log-transformed expression values. Mapping of Affymetrix probeset ids to gene ids was done using the library files made available from Affymetrix. Control probesets and probesets that did not map unambiguously to one gene were removed, specifically probeset ids ending in _x, _s, _i were removed. Lastly, if multiple probesets mapped to a single gene, then expression values were averaged within each chip."

Compared to the DISTILLER [39] data set, the DREAM5 [41] compendium contained a different subset of the available *E. coli* transcriptome measurements from a different combination of platforms. While one might expect a number of arrays to be common between the two compendia, we discovered that the two data sets differed

substantially in their statistical properties. The maximum Pearson correlation between arrays across the two data sets, for example, was ~ 0.63. Interestingly, the correlation among expression profiles of genes within predicted operons [49] was higher in the DREAM5 compendium (mean ~ 0.83) than the DISTILLER compendium (mean ~ 0.32). This is likely due to a combination of differences in the experiments/platforms included and normalization procedures.

2.2 Additional data integrated for model construction

2.2.1 Genome sequence data and annotatations for cMonkey analysis

We used genome sequences and gene annotations (coding regions) collated in RSA-tools [60] for both organisms in this study (*H. salinarium NRC-1* and *E. coli K-12 MG1655*). These data were themselves collated to annotate regulatory sequences of all sequenced genomes in RefSeq. Rather than using the RSA-tools-annotated promoter regions, we computed them ourselves as regions (-250 nt to +50 nt) surrounding the annotated translation start site of each gene/operon (see below for operon annotations).

In all cases where probe identifiers in the mRNA expression compendia used for this analysis could not be directly matched to gene annotations (or operon predictions or functional associations; see below), we used the RSA-tools "feature_names.tab" table of identifier synonyms to perform the match. In cases where the match was still not possible, we excluded the probe/ annotation/ association from analysis.

2.2.2 Operon membership predictions used for cMonkey analysis

We used operon predictions for both *H. salinarium NRC-1* and *E. coli K-12 MG1655* predicted by [49] from the Microbes Online database [2]. These predictions are updated regularly. The predictions are based upon genomic proximity and co-expression in publicly-available microarray data compendia. We used the versions downloaded from the website as of March, 2009. These included predicted operon memberships for 826 genes in *H. salinarium NRC-1* and for 2,639 genes in *E. coli K-12 MG1655*.

2.2.3 Predicted transcriptional regulators used for Inferelator analysis

2.2.3.1 *H. salinarium NRC-1*

For *H. salinarium NRC-1*, we used the same set of putative transcription factors (TFs) as [10, 9]. This list of 124 regulators was selected from among the 2,400 *H. salinarium NRC-1* genes which are annotated as known or putative TFs based upon sequence or predicted structural homology [8].

2.2.3.2 *E. coli K-12 MG1655*

To enable direct comparison of our results to DREAM5, we used the list of 296 putative *E. coli K-12 MG1655* transcriptional regulators collated by [41]. Their list was obtained by combining the list of TFs defined by RegulonDB [24] with TFs identified using Gene Ontology (GO) terms: *biological process* terms related to transcription (`GO:0009299;mRNA transcription` or `GO:0006351;transcription, DNA dependent`) and GO *molecular function* `GO:0003677;DNA binding` or any child terms.

2.2.4 Functional association networks integrated into cMonkey analysis

We used EMBL STRING [58] v9.0 database of predicted functional associations between genes for both organisms (*H. salinarium NRC-1* and *E. coli K-12 MG1655*) to constrain module construction in cMonkey, as described below. The confidence scores estimated by [58] were incorporated into the cMonkey constraints. These networks included 151,826 associations among 2,559 genes in *H. salinarium NRC-1*, and 878,972 associations among 4,136 genes in *E. coli K-12 MG1655*.

3 Independent data used for model validation

Model validation data was not used for model construction.

3.1 *H. salinarium NRC-1*

3.1.1 Tiling array transcriptome measurements

We generated *H. salinarum NRC-1* high-resolution (12 nt) tiling array transcriptome measurements over 12 points along the growth curve in rich media. These were analyzed and published in a separate study [35]. Locations of putative transcription breaks in these data were identified in [35] using multivariate recursive partitioning, including signals from both relative changes in expression along the growth curve, as well as raw RNA hybridization signal. For more details, see [35].

3.1.2 ChIP-chip transcription factor binding measurements for global regulators

Global binding of eight general transcription factors (seven TFBs [TFBa, TFBb, TFBc, TFBd, TFBe, TFBf, and TFBg] and one TBP [TbpB]) and three specific TFs (Trh3, Trh4, and VNG1451C) in *H. salinarum* were collected in our lab by ChIP-chip. A detailed protocol is described in [22]. Briefly, ChIP-enriched and amplified DNA for eleven regulators was hybridized to a low-resolution (500 nt resolution) custom PCR-product array spotted in-house. The resulting intensities were analyzed using MeDiChI [51] to obtain binding site locations with an average precision of 50 nt. Local false discovery rates (LFDRs) were quantified by simulation. For more details on the ChIP-chip analysis methodology used in this work, see [51].

3.1.3 *kdp* promoter serial truncation measurements

H. salinarum NRC-1 kdpFABC truncation data were obtained from [34]. Briefly, the authors measured relative induction of a transcriptional reporter after serial truncation of the *H. salinarum* R1 *kdpFABC* promoter. The authors measured β-Galactosidase activities from truncated transcriptional fusions of the *kdpFABC* promoter to *bgaH*. β-Galactosidase activities were measured in triplicate from cultures grown in inducing (3 mM K^+) and non-inducing (100 mM K^+) conditions. We obtained data corresponding to Figure 4 of the paper, in which the authors quantify the fractional β-Galactosidase activity (non-induced/induced) among the serial truncations (private communication). We overlaid motif predictions from EGRIN 2.0 on this data set to reach our conclusions.

3.2 *E. coli K-12 MG1655*

3.2.1 Tiling array transcriptome measurements

We measured *E. coli K-12 MG1655* tiling array transcriptome profiles at nine different time points during growth in rich media (LB). Growth phases spanned lag-phase (OD600 = 0.05) to late stationary-phase (OD600 = 7.3). RNA samples were prepared by hot phenol-chloroform extraction [33]. RNA was directly labeled and hybridized to custom Agilent tiling arrays containing 60mer probes tiled across both strands of the *E. coli K-12 MG1655* genome using a sliding window of 23 nt (GEO Platform GPL18392), as in [35]. Expression measurements were quantile-normalized as in [64] and analyzed for condition-specific transcriptional isoforms following the segmentation protocol described in [35]. Data is available on GEO (GSE55879).

3.2.2 PurR/ΔPurR expression data and ChIP-chip transcription factor binding sites

E. coli PurR/ΔPurR expression data and ChIP-chip transcription factor binding measurements collected in the presence of adenine were taken from [15]. ChIP-chip relative intensities were re-analyzed using MeDiChI [51] to obtain binding site locations with an average precision of $\sim$25 nt.

3.2.3 Fitness measurements

E. coli fitness measurements across 324 conditions were generated by [44]. In short, the authors quantitated growth rates for 3979 single gene deletions in each of 324 environments with variable stress, drug, and environmental challenges. *E. coli* mutant colony sizes were quantified on agar plates. Fitness correlations were obtained directly from the authors: http://ecoliwiki.net/tools/chemgen/. Each correlation value represents the Pearson correlation of fitness (*i.e.*, relative growth rate) for pairs of single gene deletion mutants measured across all 324 conditions that are also present in our analysis. Relative fitness scores were also obtained directly from the authors.

3.2.4 Effector molecule measurements

E. coli effector molecule measurements were taken from [28]. The authors measured metabolite levels using capillary electrophoresis time-of-flight mass spectrometry (CE-TOFMS) in *E. coli K-12 MG1655*, as well as several other biomolecules (*e.g.*., RNA and protein). *E. coli* was grown in a chemostat at several different dilution rates (0.1, 0.2, 0.4, 0.5, and 0.7 hours^{1}). We obtained the metabolite levels from the authors and computed Pearson correlation between metabolites assigned to regulate TFs by RegPrecise [46].

3.2.5 Experimentally mapped *E. coli* transcription factor binding sites

We compared genome-wide locations of GREs in the *E. coli* EGRIN 2.0 model with experimentally-mapped binding sites from the RegulonDBdatabase [24]. To maintain consistency with our comparisons against the DREAM5 community networks [41], we used version 6.8 of the database. For binding sites, we used the BindingSiteSet table, filtered for only interactions with experimental evidence, and used only TFs with ≥ 3 unique binding sites – a total of 88 TFs.

3.2.6 Experimentally measured *E. coli* transcription factor regulatory targets

For the *E. coli* gold standard network, we used the same network as that used by [41] for validation of the DREAM5 *E. coli* community predicted regulatory networks. This gold standard is based upon version 6.8 of the RegulonDB database [24], and only interactions with at least one strong evidence were included, for a total of 2,066 interactions. We mapped the $aaaX$-style gene names in the DREAM5 gold standard to the $b1234$ in cMonkey using a translation table compiled in the EcoGene database, version 3.0 [66]. We were able to map a total of 4,273 gene names. The final gold standard consisted of 2,064 interactions between 141 TFs and 997 target genes. The final, complete gold standard network used for all analyses is available online.

4 Computational methods

4.1 cMonkey: integrated biclustering algorithm, updated for ensemble analysis

4.1.1 Introduction and summary

The cMonkey integrated biclustering algorithm was described and fully benchmarked in [50]. In short, the algorithm computes putatively co-regulated modules of genes over subsets of experimental conditions from gene expression data, constrained by information provided by genome sequence (*de novo* identification of conserved *cis*-regulatory motifs in gene promoters), and functional association networks. Its defining characteristic is that it combines all three types of data (expression, sequence and networks) together into an integrated model that uses a stochastic optimization procedure to identify modules that best satisfy all three constraints, simultaneously.

We refer the reader interested in details of the cMonkey data integration model and optimization procedure to [50]. Here, we briefly summarize the procedure as it was utilized in the EGRIN 2.0 model construction. The cMonkey integrated biclustering algorithm identifies groups of genes co-regulated under subsets of experimental conditions, by integrating various orthogonal pieces of information that support evidence for their co-regulation, and optimizing biclusters such that they are supported by one or more of those additional constraints. The three sources

of evidence for co-regulation leveraged by cMonkey to score gene clusters are (1) tight co-expression in subsets of available gene expression measurements (similarity of expression profiles); (2) quality of *de novo* detected *cis*-regulatory motifs in gene promoters (putative co-binding of common regulators); and (3) significant connectivity in functional association or physical interaction networks (co-functionality). The algorithm served as the cornerstone for the construction of the first global, predictive Environmental Gene Regulatory Influence Network (EGRIN) model for *H. salinarium NRC-1*[9], and has now been applied to many additional organisms (*e.g.*, [65] and unpublished).

To run cMonkey as part of an ensemble-based inference approach required significant updates to the cMonkey algorithm. These updates primarily addressed computational inefficiencies that led to long runtime. The primary algorithm modification in the new implementation is global optimization (rather than the local, individual cluster optimization utilized by the original procedure). Additional algorithm updates include changes to the individual scoring scheme for subnetwork clustering, as well as integration of the scores. All of these changes improved the procedure's runtime without significantly affecting the algorithm's performance.

4.1.2 Updates since original publication

For incorporation into the EGRIN 2.0 ensemble analysis, the cMonkey procedure and software was overhauled to improve runtime performance and decrease memory usage. These modifications did not quantifiably affect overall bicluster quality. Changes to the algorithm (and parameters used for EGRIN 2.0 construction) relative to the earlier version described in [50] are as follows:

1. Iteratively re-weighted constrained logistic regression to determine gene/condition probabilities for bicluster membership was replaced with a non-parametric cumulative distribution function on gene/condition scores. Since the non-parametric function does not need to be re-weighted, it is significantly faster to compute.

2. Rather than constraining the number of bicluster assignments per gene/condition using a probability distribution, cMonkey now assigns a fixed number of biclusters to each gene/condition, per run (a user-defined parameter). For this study the parameter was set to 2 for genes, and to $k/2$ for conditions, where k is the total number of biclusters in the run, also a user-defined parameter. This modification effectively altered the bicluster optimization from a local problem (single bicluster) with limited cross-bicluster constraints to a global problem, similar in principle to the widely used k-means clustering algorithm.

3. Since cMonkey uses the updated constraint (of 2; see above) to choose the two "best" biclusters for each gene (and likewise the best $k/2$ biclusters for each condition), there is no sampling as in the prior version. Instead, stochasticity is added to prevent the optimization from falling into local minima. The algorithm allows at most one change in bicluster assignment per gene/condition, per iteration. This is accomplished by adding a small amount of noise to each gene/condition's bicluster membership weight. The noise occasionally allows moves that decrease a bicluster's total score. This noise decreases towards zero as the number of iterations increases.

4. Motif detection is the most computationally expensive part of the procedure. To circumvent significant computation time, we limit motif detection to every 100 iterations (for a typical, 2,000 iteration cMonkey run). During the 99 iterations between motif searches, the biclusters are optimized to contain instances of those detected motif(s). We found that this does not impair the ability of cMonkey to detect motifs.

The overall effect of these changes (in addition to other minor modifications and improvements) resulted in an algorithm run-time reduction of about 4-fold. This, in turn, enabled cMonkey to be run numerous times on a modest 8-core compute node (*e.g.*, a c1.xlarge Amazon EC2 node) in under six hours per run (compared to several days to a week for the original cMonkey). Practically, the effect of these modifications to the algorithm resulted in a single cMonkey run generating fewer duplicate biclusters, primarily because each gene is allowed to be a member of only two biclusters. We found that this increased the overall diversity of regulation discovered by each cMonkey run (condition-specific gene clusters and corresponding *cis*-GREs).

4.1.3 Detailed cMonkey algorithm description

The cMonkey algorithm initiates by seeding k biclusters, typically using the simple, widely-used and effective k-means clustering on the input expression data set. cMonkey itself performs a global optimization, in many ways similar to the k-means clustering algorithm, which we used as a model. After beginning with an initial assignment

of each gene into k clusters and a chosen distance metric, the basic k-means algorithm iterates between two steps until convergence: (1) (re-)assign each gene to the cluster with the closest centroid and (2) update the centroids of each modified cluster. The updated cMonkey algorithm performs an analogous set of moves with four primary distinctions: (1) the distance of each gene to the "centroid" of each cluster is computed using a measure that combines condition-specific expression profile similarity, cis-regulatory motif similarity, and connectedness in one or more gene association networks; (2) each gene can be (re-)assigned to more than one cluster (default 2); (3) at each step, conditions (in addition to genes) are moved among biclusters to improve their cohesiveness; and (4) at each step, genes and conditions are not always assigned to the most appropriate clusters. We now elaborate upon these four details.

cMonkey begins each iteration with a set of bicluster memberships $\{m_i\}$ for each element (gene or condition) i, where by default $\|m_i\| = 2$ for genes and $\|m_i\| = N_c/2$ for conditions (N_c is the number of conditions, or measurements, in the expression data set; note that for standard k-means clustering, $\|m_i\| = 1$ for genes and $\|m_i\| = N_c$ for conditions). cMonkey then computes score matrices (log-likelihoods, in practice) $\mathbf{R}_{ij}$, $\mathbf{S}_{ij}$, and $\mathbf{T}_{ij}$, for membership of each element i in each bicluster j, based upon, respectively, co-expression with the current gene members ($\mathbf{R}$), similarity of motifs in gene promoters ($\mathbf{S}$), and connectivity of genes in networks ($\mathbf{T}$). For the network scores ($\mathbf{T}$), the originally published procedure [50] computed a p-value for enrichment of network edges among genes in each bicluster using the cumulative hypergeometric distribution. This computation was inefficient, and moreover could not account for weighted edges in the input networks, so we replaced it with a more standard weighted network clustering coefficient [61], evaluated only over the genes within each bicluster.

Following computation of the individual component scores, cMonkey computes a score matrix $\mathbf{M}_{ij}$ containing the integrated score (a weighted sum of log-likelihoods) supporting the inclusion of gene i in bicluster j. At this stage the updated version of cMonkey then computes a cumulative density distribution from each bicluster's $\mathbf{M}_{\cdot j}$ to obtain a posterior probability distribution p_{ij}, that each element i should be in each cluster j, which is used to classify cluster members based upon these scores. The width of the density distribution kernel is set dynamically to be larger for smaller (fewer gene) biclusters, so as to increase the tendency to add genes to small biclusters, rather than to remove them. In the updated procedure, we then add a small amount of normally-distributed random "noise:" to the scores $\mathbf{M}_{ij}$, in order to achieve a similar type of stochasticity to the original version of the algorithm (which was originally obtained using sampling, and which helps prevent the algorithm from falling into local minima; this noise decreases during the run to zero at the final iteration). The result of this noise is that at the beginning of a cMonkey run, biclusters are rather poorly defined (co-expression, for example, is weak), but during the course of a full set of 2,000 iterations, as this noise is decreased, the biclusters settle into minima.

Finally, at the end of each iteration, cMonkey chooses a random subset of elements (genes or conditions) i, and moves i into bicluster j if, for any biclusters j' which it is already a member, $p_{ij} > p_{ij'}, \forall j'$, and out of the corresponding worse bicluster j' for which $p_{ij} > p_{ij'}$. Thus, as with the k-means clustering algorithm, the updated cMonkey performs a global optimization of all biclusters by moving elements among biclusters to improve each element's membership scores.

4.1.4 Parameter ranges used for EGRIN 2.0

The default values for all additional parameters used for cMonkey, and for MEME (which is used by cMonkey for motif detection; [3]), are the same as those itemized in [50]. These defaults were used exclusively for all *H. salinarium NRC-1* cMonkey runs. These default parameters are itemized in Table S1. The only parameter that varied from run-to-run for the *H. salinarium NRC-1* cMonkey runs was the number of conditions (columns in the expression matrix) included. As cMonkey development was occurring in parallel to development of the EGRIN 2.0 modeling approach (primarily involving bug fixes), we also list the official cMonkey version number(s) used.

In contrast to the *H. salinarium NRC-1* runs, for the *E. coli K-12 MG1655* runs, we varied several parameters randomly between the ranges itemized in Table S2.

Parameter	Value	Note
Version	4.5.4(174), 4.6(191), 4.6.2(109)	cMonkey package versions (and number of runs) used
N_{conds}	242:300	Number of conditions included (range)
k	250	Number of biclusters
N_{gene}	2	Number of biclusters per gene
N_{iter}	2000	Number of iterations
w_{max}	24	Maximum MEME motif width
w_{min}	6	Minimum MEME motif width
l_{search}	-150 – +20	MEME search distance from gene start
l_{scan}	-250 – +30	MEME scan distance from gene start
n_{motif}	2	Number of MEME motifs searched per bicluster
s_{r}	1	Scaling factor for expression scores
s_{m}	1	Scaling factor for motif scores
s_{n}	0.5	Scaling factor for network scores
w_{op}	0.5	Relative weight for operon network
w_{string}	0.5	Relative weight for STRING network

Table S1: cMonkey parameters used for the *H. salinarium NRC-1* ensemble.

Parameter	Value	Note
Version	4.9.0(106)	cMonkey package versions (and number of runs) used
N_{conds}	181:279	Number of conditions included (range)
k	352:547	Number of biclusters
N_{gene}	2	Number of biclusters per gene
N_{iter}	2000	Number of iterations
w_{max}	12:30	Maximum MEME motif width
w_{min}	6	Minimum MEME motif width
l_{search}	-(100:200) – +(0:20)	MEME search distance from gene start
l_{scan}	-(-150:250) – +(0:50)	MEME scan distance from gene start
n_{motif}	1:3	Number of MEME motifs searched per bicluster
s_{r}	2:4	Scaling factor for expression scores
s_{m}	0.5	Scaling factor for motif scores
s_{n}	0.5	Scaling factor for network scores
w_{op}	0:1	Relative weight for operon network
w_{string}	0:1	Relative weight for STRING network

Table S2: cMonkey parameters used for the *E. coli K-12 MG1655* ensemble.

4.1.5 cMonkey software availability

The cMonkey software is available as an open-source R package [27]. With this package the algorithm can be easily applied to nearly any sequenced microbial species (given user-supplied expression data). The package automatically downloads and integrates genome and annotation data from various external sources, including RSA-tools [60]; Microbes Online [2]; and EMBL STRING [58]. Additionally, the package can generate interactive web-based and Cytoscape output [56], allowing users to explore the resulting modules and motifs in the context of external data, software, and databases via the Gaggle [57]. Examples of automatically generated output are available at the cMonkey web site. Supplementary R packages with example expression data for organisms including *H. salinarium NRC-1* and *E. coli K-12 MG1655* are also available from the cMonkey website.

4.2 Inferelator: inference of transcriptional regulatory influences

4.2.1 Introduction and summary

The Inferelator algorithm is a method for deriving genome-wide transcriptional regulatory interactions from mRNA expression levels [10]. Inferelator is a direct inference procedure [43]. It models transcriptional regulation as a kinetic process, incorporating time information, when available, and a user-defined time constant. Inferelator uses standard regression and variable selection to identify transcriptional influences on genes or biclusters based on their mean expression levels. These influences include expression levels of TFs, environmental factors, and interactions between the two. The procedure simultaneously models equilibrium and time course expression levels. Thus both kinetic and equilibrium expression levels may be predicted by the resulting models. Through explicit inclusion of time and gene knockout information, the method is capable of learning causal relationships. The inferred network is a predictive model comprised of linear combinations of expression profiles of various transcriptional regulators, that can predict global expression under novel perturbations with predictive power similar to that seen over training data [10].

4.2.2 Updates since original publication

Several modifications have been made to improve the originally published Inferelator algorithm [10].

1. Elimination of pre-grouping highly correlated regulators into "TF groups". This step became obsolete by replacing the L_1 (LASSO) constraint with the elastic-net linear regression constraint. It has been shown that the elastic-net constraint results in highly correlated predictors being grouped as part of the optimization. This relieves the necessity of pre-grouping predictors [67]. We note that in all Inferelator runs the elastic-net parameter value α was set to $\alpha = 0.8$ (*i.e.*, close to the original LASSO L_1 constraint (corresponding to $\alpha = 1$), but with a "small amount" of L_2). We used the elastic-net implementation provided by the R `glmnet` package [23]

2. Elimination of the "pre-filtering" of regulators for each bicluster based upon high correlation. The procedure now allows the elastic-net to choose among all potential regulators (excluding TF members of the bicluster, which are automatically considered possible regulators, and are removed from the list of candidate predictors prior to applying the elastic-net).

3. Capability to up-weight measurements. This was utilized in the EGRIN 2.0 model to up-weight measurements with lower variance (*i.e.*, more tightly co-expressed) among the genes in a bicluster by standard weighted linear least-squares, $w_i = 1/\sigma_i^2$.

The current implementation of Inferelator is available as an open-source R package.

4.2.3 Detailed algorithm description

Given an input list of p putative transcriptional influences $\mathbf{X} = x_1, x_2, \cdots, x_p$ and the mean expression levels y_i of a bicluster k (over the conditions i included in the bicluster), we model the relationship between y_i and the influences $\mathbf{X}$ by the kinetic equation:

$$\tau \frac{dy_i}{dt} = -y_i + \sum_{j=1}^{p} \beta_j x_{ij}. \qquad (1)$$

In the steady state scenario, $dy/dt = 0$ and Eq. 1 simplifies to

$$y_i = \sum_{j=1}^{p} \beta_j x_{ij},$$

and for time series measurements, we approximate Eq.1 as:

$$\tau \frac{y_{i+1} - y_i}{t_{i+1} - t_i} + y_i = \sum_{j=1}^{p} \beta_j x_{ij}.$$

Clearly not all p putative influences $\mathbf{X}$ influence a given bicluster, so we use the elastic-net [67] for variable selection. This involves performing the minimization:

$$\vec{\beta} = \arg\min \left\{ \sum_{i=1}^{N} \left(y_i - \sum_{j=1}^{p} \beta_j x_{ij} \right)^2 + \sum_{j=1}^{p} \boldsymbol{w}_i (\lambda_1 |\beta_j| + \lambda_2 \beta_j^2) \right\} \tag{2}$$

subject to a constraint which is a tuneable combination of the L_1 (LASSO) and L_2 (Ridge) regression constraints:

$$\sum_{j=1}^{p} |\beta_j| \leq \lambda_1 |\beta_{\text{ols}}| \qquad (L_1 \text{ constraint}),$$

$$\sum_{j=1}^{p} \beta_j^2 \leq \lambda_2 \beta_{\text{ols}}^2 \qquad (L_2 \text{ constraint}).$$

The w_i in Eq. 2 allow different variables (β's, in this case) to be selectively constrained. For this work, we set all $w_i = 1$, *i.e.*, no differential constraints. Redefining the constraint, such that:

$$\vec{\beta} = \arg\min \left\{ \sum_{i=1}^{N} \left(y_i - \sum_{j=1}^{p} \beta_j x_{ij} \right)^2 + \sum_{j=1}^{p} \boldsymbol{w}_i \lambda \left(\alpha |\beta_j| + (1-\alpha) \beta_j^2 / 2 \right) \right\} \tag{3}$$

defines $0 \leq \alpha \leq 1$ as a tuning parameter between the ridge (L_2; $\alpha = 0$) and LASSO (L_1; $\alpha = 1$) solutions, and λ is the single complexity parameter, which is chosen to minimize the cross-validation error (we use 10-fold cross-validation), exactly as in [10]. Substituting Eq. 1 into Eq. 3, we obtain the complete equation describing the minimization performed by Inferelator:

$$\vec{\beta} = \arg\min \left\{ \sum_{i=1}^{N} \left(\tau \frac{y_{i+1} - y_i}{t_{i+1} - t_i} + y_i - \sum_{j=1}^{p} \beta_j x_{i,j} \right)^2 + \sum_{j=1}^{p} \boldsymbol{w}_i \lambda \left(\alpha |\beta_j| + (1-\alpha) \beta_j^2 / 2 \right) \right\}. \tag{4}$$

For the current implementation, we set $\tau = 10$ minutes for all TFs, and $\alpha = 0.8$ for all biclusters. In the future, we could choose τ and/or α by cross-validation as well. When $\alpha = 0$, there is no constraint, and we get the ordinary least-squares (OLS) solution with all βs non-zero. With $\alpha = 1$, we select the null model. The optimal solution is somewhere in-between, and this is usually the selected solution for each bicluster, usually ~ 6 TFs, on average; although the null model (no solution) is selected for a number of biclusters.

4.3 EGRIN 2.0 model construction

4.3.1 Background and motivation

The procedure to infer a single global Environment and Gene Regulatory Influence Network (EGRIN) model from genome-wide data was described previously [9, 10, 50]. In short, the two-step procedure involves running cMonkey once to obtain a single set of ~ 300 biclusters of genes. Genes in these biclusters have tight co-expression over a subset of the measured conditions (usually about half), are supported by common putative *cis*-regulatory motif(s) in their promoters (gene regulatory elements, GREs), and are often substantiated by high connectivity in functional association networks. Next, given a set of "predictors" (mRNA expression levels of transcription factors and/or quantitative values for environmental factors; *e.g.*, concentrations, growth media, etc.), and the mean expression levels of genes in each bicluster, Inferelator is run to choose a parsimonious subset of those predictors that can accurately predict the expression levels of that bicluster (*i.e.*., those with non-zero β [Eq 4]) . Predictors are selected independently for each bicluster. The combined set of TF→bicluster interactions and their associated weights (βs) give the degree of activation (or repression) predicted.

The EGRIN 2.0 modeling procedure updates this process by applying updated cMonkey and Inferelator algorithms (described above) repeatedly to subsets of the available expression data. The end result is an ensemble of EGRIN models, each model containing biclusters and their predicted regulators, tuned to a relatively small subset

of the overall input expression compendium. The experimental subsets were selected semi-randomly, with available biological information constraining the selection procedure (*i.e.*., including whole groups of related experiments when one was randomly selected). For *H. salinarum*, we used manually curated metadata about each experiment to group related experiments. Since we did not have sufficient metadata from the public *E. coli* data set, we grouped the conditions based upon individual experiments instead (*e.g.*, time series).

The EGRIN 2.0 inference methodology is an ensemble learning approach, more specifically a form of bootstrap aggregation [11], or sub-bagging. Advantages of sub-bagging include simplicity (*i.e.*, basic model averaging), reduced model variance compared to individual runs [13], and avoidance of overfitting [36]. The power of ensemble learning approaches stems from their ability to average out errors in individual models. For EGRIN models, this feature helps overcome artifacts due to both experimental and algorithmic noise. Incorrect classification in a single model that are not the result of systematic error will re-occur infrequently in subsequent runs. Similarly, overfitting is mitigated by training each individual model on a small subset of the available data. Only consistently re-discovered relationships are considered significant.

Sub-bagging of experimental conditions further allows the model to effectively up-weight a restricted set of conditions for each individual EGRIN model in the ensemble. This forces each EGRIN to model regulatory behaviors present within a more narrow range of conditions. As a result, the individual EGRIN models have the opportunity to discover features that may distinguish highly related responses or occur in a very limited number of conditions in the data set (*e.g.*, conditions, genes, GREs).

To quantify this assumption, we constructed a separate ensemble of 30 EGRIN models trained on the complete *H. salinarum* data set (*i.e.*, 1,495 conditions; no sub-setting performed). We asked how often we would discover a GRE corresponding to the well-characterized anoxic *H. salinarum* TF, Bat. Given frequent detection of the Bat GRE in our full ensemble, we expected to detect ~ 20 instances of the Bat GRE in the new ensemble (*i.e.*, motifs similar to GRE #22; Figure S2 [6]). Surprisingly, we did not detect a single GRE matching Bat when all conditions were used for training (data not shown). This is likely because the anoxic conditions in which Bat is active represents only a small portion of the entire data set.

Ensemble-based approaches are being used more frequently in biological data analyses, including random forests (*i.e.*, bags of decision trees) [12], and the recently-published DREAM5 community ensemble of regulatory network predictions [41], which we used as a benchmark in this manuscript to evaluate EGRIN 2.0 predictions for *E. coli K-12 MG1655*. Moreover, in principle, our approach is similar to the stochastic LeMoNe algorithm [29], which uses Gibbs sampling to learn ensembles of regulatory modules from gene expression data. EGRIN 2.0 is distinguished from LeMoNe and similar algorithms by its ability to predict transcriptional control mechanisms (*i.e.*, GREs) and the conditions in which they operate, both globally and within individual gene promoters.

To construct and mine the EGRIN 2.0 ensemble we utilized additional model aggregation and compilation procedures, including (1) motif clustering [59] and scanning [3] (Section 4.3.3); (2) gene co-regulation network construction and backbone extraction [55] (Section 4.3.5.1); and (3) network community detection [1] (Section 4.3.5.3). These methods were used to identify GREs and their genome-wide locations, gene-gene co-regulatory associations, and corems, respectively. Each of these procedures is described in more detail below. A comprehensive workflow is provided in Figure S1. Statistics for the two models is provided in Table S3.

4.3.2 "Ensemble of EGRINs": generation and statistical mining

EGRIN 2.0 model construction and analysis was performed using primarily the R statistical analysis environment, with add-on packages data.table and filehash for off-line storage (maintaining all information in memory was impossible for our large ensembles). Once the full set of cMonkey and Inferelator runs were completed and stored, a round of post-processing was performed to agglomerate all results into a single ad-hoc database for storage and query. The following relationships could be queried to identify significant associations between biological entities described in the model:

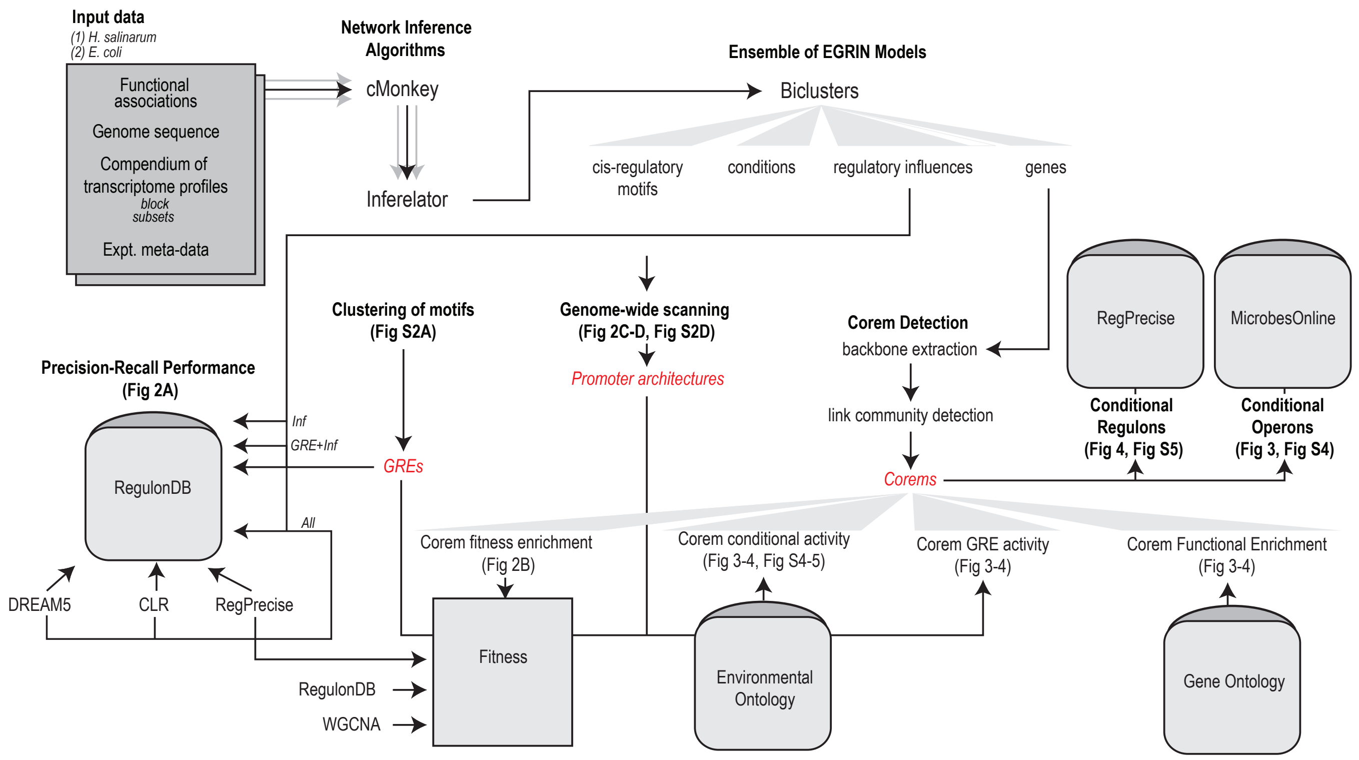

Figure S1: **Detailed workflow for EGRIN 2.0 inference procedure.** Data input, processing and analysis to construct EGRIN 2.0 model for *H. salinarum* and *E. coli*, and predictions generated. Predictions highlighted in individual figures are noted.

Entity_1	Entity_2	Relationship	Associated info.
Bicluster	Gene	Contains	-
Bicluster	Condition	Contains	-
Bicluster	Motif	Contains	Associated genes
Regulator	Bicluster	Regulates	Weight
Motif	Motif	Similar	FDR q–value
Motif	Genomic coordinate	Overlaps	p-value

These relationships could then be extended to second-degree relationships, including (these relationships below are by no means all-inclusive; for brevity we denote g, g_1, and g_2 as separate genes, b as a bicluster, m as a motif, r as a regulator, and c as an experimental condition):

1. g_1 is co-regulated with g_2 if they occur in the same b.
2. g_1 is co-regulated with g_2 under condition c if g_1, g_2, and c occur in the same b.
3. m regulates g if m and g are both observed in the same b.
4. m regulates g under condition c if m, g, and c are all observed in the same b.
5. r putatively regulates gene g via m if r is predicted to regulate b which contains both g and m.

The frequency with which any of these relationships occurs throughout the entire ensemble of EGRIN models could subsequently be counted by querying the database, and a p-value describing the significance of the frequency computed via the cumulative hypergeometric distribution. p-values were then converted to false discovery rate q-values using the BenjaminiHochberg procedure. We use this basic procedure to identify conditions associated with GRE influence, and GREs associated with gene co-regulation, as we describe below.

**Statistic**	***H. salinarum***	***E. coli***
Arrays analyzed	1495	868
Genes/transcripts	2400	4213
Number of EGRIN models constructed	572	106
Number of cMonkey biclusters	142600	46520
Fraction of ”good” cMonkey biclusters	61%	43%
Residual cutoff defining ”good” biclusters	0.40	0.55
Number of genes/transcripts in $\leq$ 1000 ”good” biclusters (Hal)	2104	
Number of genes/transcripts in $\leq$ 200 ”good” biclusters (Eco)		4201
Average number of biclusters per gene	1210	212
Number of motifs (total)	269770	86167
Number of motifs (E-value $\leq$ 1)	118546	15588
Number of motifs (E-value $\leq$ 1e-6)	32739	3506
Number of motif clusters	162	402
Number of motifs contained in motif clusters	37713	13519
Number of unique GREs	135	337
Number of motifs in unique GREs	27991	12773
Number of RegulonDB GREs detected (p $\leq$ 0.01)		53
RegulonDB TFs with $\geq$ 3 experimentally characterized binding sites		86
Corems	679	590
Genes modeled by corems	1363	1572
Genes per corem: min(max)	3(377)	3(153)
Active conditions per corem: min(max)	21(1279)	69(598)
GREs per corem: min(max)	0(9)	0(12)
Co-regulatory associations: prior to backbone extraction	1573836	3094954
Co-regulatory associations: after backbone extraction	141850	170723
Co-regulatory associations: corems	56738	25976

Table S3: Global properties of *H. salinarum* and *E. coli* ensembles

4.3.3 Clustering of cis-regulatory motifs to identify GREs

Each cMonkey bicluster contains at least one *de novo* MEME- detected [3] *cis*-regulatory motif. These motifs are used by cMonkey to guide bicluster optimization (in addition to other scoring metrics). There were 86,167 and 269,770 motifs detected across the entire ensemble for *E. coli* and *H. salinarum*, respectively. Each motif was represented in the model as a position-specific scoring matrix (PSSM). To determine which of these motifs represented *bona fide* GREs (as opposed to false positives), we computed pairwise similarities between all motifs using Tomtom [25] (Euclidean distance metric; minimum overlap of 6 nt) and clustered the most highly similar PSSM pairs using mcl [59].

The Tomtom motif similarity p-value threshold and the mcl inflation parameter (I) were selected to (1) maximize the density (unweighted) of edges between PSSMs inside clusters relative to the edges between clusters, and (2) ensure that the mcl “jury pruning synopsis” was at least 80 (out of 100). Criterion (1) aims to find a clustering that is as inclusive as possible, while minimizing over-clustering, while (2) is a built-in mcl metric that evaluates the quality of the clusters resulting from the user-selected pruning strategy (I). More specifically for criterion (1), we chose the clustering parameters (mcl inflation parameter I, Tomtom p-value cutoff p_c) which maximize:

$$(I, p_c) = \arg\max \left\{ \sum_{I=1}^{N} \sum_{i=1}^{n_I} \frac{\sum_{j=1}^{n_I} \delta_{ij}}{\sum_{J=1}^{N} \sum_{k=1}^{n_J} \delta_{ik}} \right\}, \quad (5)$$

where N is the total number of motif clusters for a given set of parameters, δ_{ij} indicates a significant similarity (subject to the given p-value threshold) the between PSSMs i and j within motif cluster I (which contains a total of

n_I PSSMs), and δ_{ij} indicates a significant similarity between PSSM i in motif cluster I and PSSM j in motif cluster J. The final parameters that maximized expression 5 and resulted in an mcl "jury pruning synopsis" of at least 80 were different for the two EGRIN 2.0 models: $p_c = 10^{-6}$ and mcl $I = 4.5$ for the *H. salinarum* ensemble and $p_c = 10^{-5}$ and mcl $I = 1.5$ for the *E. coli* ensemble.

We did not filter the motifs by E-value or other intrinsic motif quality metrics; rather, we enforced a cluster size threshold to ensure that GREs were re-detected consistently. Clusters containing at least 10 PSSMs were considered GREs. This criterion resulted in 135 GREs for *H. salinarum* (representing 27,991 PSSMs, Table E2) and 337 for *E. coli* (representing 12,773 PSSMs, Table E3). Finally, we computed a "combined PSSM" for each GRE as the unweighted mean of aligned PSSMs within each cluster. This combined PSSM could be visualized as a motif logo identically to standard motif PSSMs.

The motif clustering procedure is summarized in Figure S2.

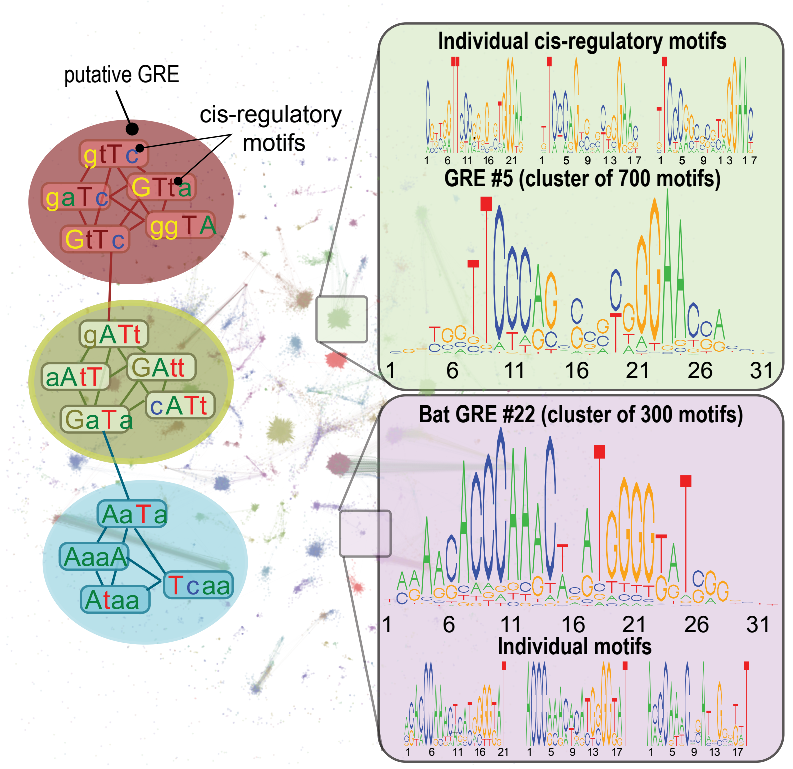

Figure S2: **Motif clustering and GRE identification.** (Left) A schematic of the approach used to align and cluster individually detected motifs to define GREs. In this example, similar motifs were aligned and clustered into three GREs using Tomtom and mcl (Details in Methods and Supplementary Methods). (Center) The *H. salinarum* network of aligned and clustered motifs. (Right) Two *H. salinarum* GREs discovered by this method. The motif logo of each GRE was generated by summing PSSMs of the individual aligned motifs in the cluster, as illustrated by three examples of individual motifs (prior to alignment) for each of the two GREs. Note that relative to the individual motifs, the averaged GRE motif is more palindromic - a hallmark of binding sites for dimeric TFs.

4.3.4 Genome-wide scanning of motifs to obtain GRE locations

We used motif scanning to discover GRE locations that were missed by the rigid definition of a promoter in cMonkey (typically -250 to +50 nucleotides surrounding the translation start site). This procedure was critical for discovering GREs in non-canonical locations, such as internal to operons. We computed how well each PSSM (described above) matched every position in the genome using MAST [3], and recorded significant matches at each genomic location subject to a position p-value threshold of 10^{-5}. This p-value cutoff corresponds to an expectation of discovering ~ 20 sites at random across the genome. For each GRE, we summed the number of significant matches to each of

the GREs PSSMs at each genomic position. These counts were used to represent GRE composition in promoters (Figures 2-3). In addition, we used these scanned locations to identify GREs located predominantly inside coding regions. Since these GREs may be spurious (*e.g.*, protein sequence motifs or trinucleotide patterns) they were flagged, although they were not removed from our global analysis.

We compared the genome-wide distribution of GRE locations to annotated start sites in *H. salinarum*. We discovered that most GREs occur in consistent locations with respect to gene start sites. The global position of all GREs and select GREs relative to experimentally determined gene start sites is depicted in Figure S3.

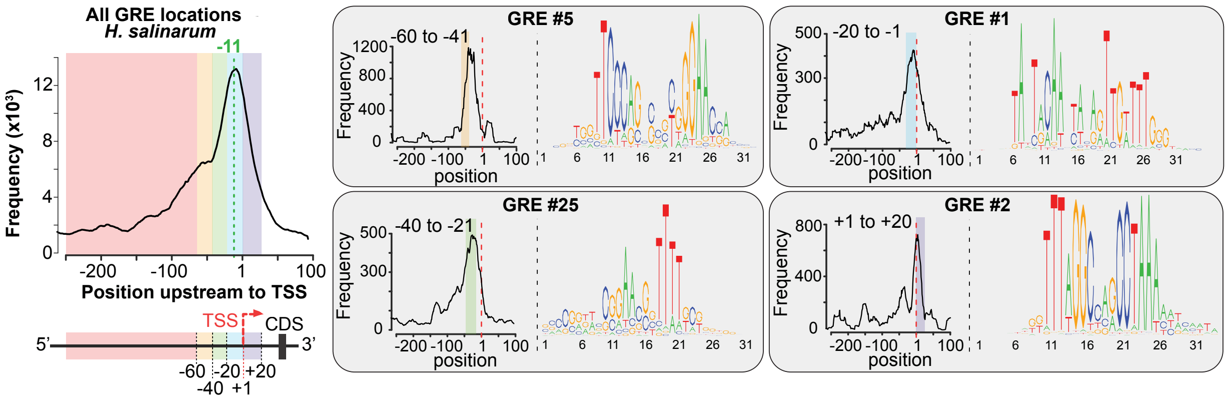

Figure S3: **Genome-wide distribution of GREs relative to experimentally mapped transcriptional start sites in *H. salinarum*.** (Left) Predicted positions for all GREs in gene promoters upstream of experimentally mapped transcription start sites (TSSs; [35]) in and (Right) four example elements. Distribution peaks for most GREs occur at characteristic locations. For instance, the location of TATA box-like elements (GRE #25) between -21 to -40 nt upstream to TSSs in *H. salinarum* is consistent with the characterized location of basal elements in archaeal promoters (-25 to 30 nt upstream to TSS). GRE location enables prediction of putative roles for the cognate TF (*e.g.*repressor, activator or a basal factor).

4.3.5 Identifying corems

4.3.5.1 Gene-gene co-occurrence network

We post-processed the EGRIN 2.0 ensemble to refine the underlying network structure and discover functionally meaningful gene co-regulatory modules present in the model. To do so, we transformed the ensemble of biclusters into a weighted gene-gene association graph G, where the nodes of G are genes and the weight of edges between the nodes is proportional to their frequency of co-occurrence in biclusters:

$$w_{ij} = \frac{|B_i \cap B_j|}{\min(B_i, B_j)}, \quad (6)$$

where w_{ij} is the weight of the edge between genes i and j, B_i is the set of all biclusters containing gene i. The weights were normalized by the minimum number of biclusters containing either gene, rather than by the more typically applied union (which would make the score identical to the Jaccard Index) to avoid penalizing genes that occur infrequently in biclusters. The sum of edge weights for each gene was normalized to one. This gene-gene co-occurrence network represents how often cMonkey discovers co-regulation between every pair of genes in the genome. We note that since this network is derived from biclusters, it is also a reflection of conditional co-expression and predicted *cis*-regulatory motifs.

4.3.5.2 Network backbone extraction

After transforming the ensemble into a normalized graph, we removed edges that were statistically indistinguishable by multiscale backbone extraction (null hypothesis of uniform edge weight distribution given a node of degree k)

[55]. We retained all edges satisfying the following relation:

$$\alpha_{ij} = 1 - (k-1)\int_0^{w_{ij}} (1-x)^{k-2}dx \leq 0.05, \tag{7}$$

where α_{ij} is the probability that the normalized weight w_{ij} between genes i and j is compatible with the null hypothesis, and k is the degree of gene i. For *H. salinarium NRC-1*, backbone extraction reduced the number of regulatory edges from 1,576,643 to 141,667; in *E. coli K-12 MG1655* the number of edges was reduced from 3,094,954 to 170,723.

4.3.5.3 Network link-community detection

Following backbone extraction, we detected corems by application of a recently described link-community detection algorithm [1]. For this algorithm to work on our data set we modified it to accept input of a weighted graph [30]. We implemented it in C++ for efficiency. The algorithm computes a similarity score between all pairs of edges sharing a common keystone node, k, according to the Tanimoto coefficient, T:

$$T(e_{ik}, e_{kj}) = \frac{a_i \cdot a_j}{|a_i|^2 + |a_i|^2 + a_i \cdot a_j}, \tag{8}$$

where

$$a_i = w_{ij} + \frac{\delta_{ij}}{k_i}\sum_{l \in n(i)} w_{il}. \tag{9}$$

Here, e_{ik} is the edge between gene i and the keystone gene k, and δ_{ij} is the Kroenecker delta. The score reflects the similarity of gene neighborhoods adjacent to two edges sharing a gene, with the score increasing in value as the number and weight of overlapping adjacent edges increases. To transform the Tanimoto coefficient into a distance metric, we compute $1 - T$.

Following scoring, the edges were aggregated by standard hierarchical clustering. The resulting tree is cut at many thresholds to optimize the local weighted density D of the resulting clusters:

$$D = \frac{1}{M\langle w\rangle}\sum_{c \in C} m_c \langle w\rangle_c \left(\frac{m_c - (n_c - 1)}{n_c(n_c-1)/2 - (n_c - 1)}\right), \tag{10}$$

where M is the total number of edges in the entire network, $\langle w\rangle$ is the average weight of edges in the entire network, C is the set of all link communities at a given threshold, m_c is the number of edges in community c, $\langle w\rangle_c$ is the average weight of edges in community c, and n_c is the number of genes in community c. The density scoring metric D had a clear optimum corresponding exactly to the cutoff that would have been chosen had we used the unweighted scoring metric originally described (Figure S4). Only communities with more than two genes were retained.

Since the communities produced by this algorithm are comprised of sets of edges, we defined a corem to include all genes incident to the edges in a community. Because of this definition, each gene can be a member of multiple different corems. In *H. salinarum*, this procedure generated 679 corems ranging in size from 3 to 377 genes, covering 1,363 of the 2,400 genes in the genome, and comprising 56,738 co-regulatory associations. In *E. coli*, we discovered 590 corems, ranging in size from 3 to 153 genes, covering 1,572 of 4,213 genes and 25,976 regulatory edges. See Table E1 and Figure S4 for additional statistics. Gene-to-corem and corem-to-gene mappings for the *H. salinarum* and *E. coli* models are available online.

4.4 Functional enrichment estimates for genes in corems

We computed functional enrichment for genes organized into corems using DAVID [19] and the DAVIDQuery [16] R-package. Enrichments for each corem are available on the web site.

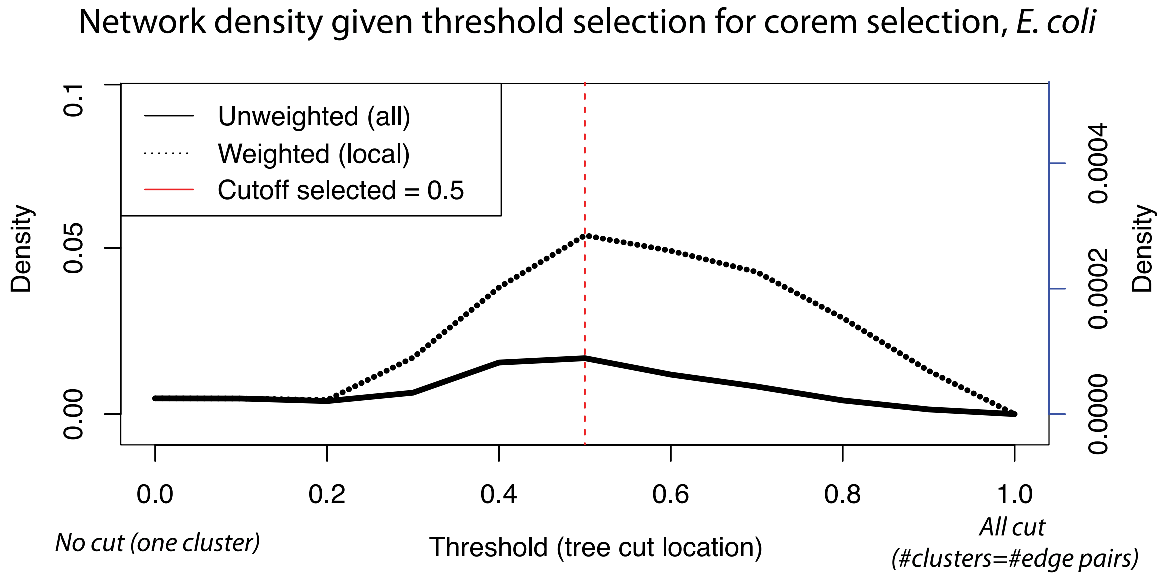

Figure S4: **Corem density as a function of clustering cutoff threshold.** Hierarchical clustering cut threshold chosen to maximize the density of resulting clusters. The cutoff chosen with modified weighted density metric is identical to unweighted density metric.

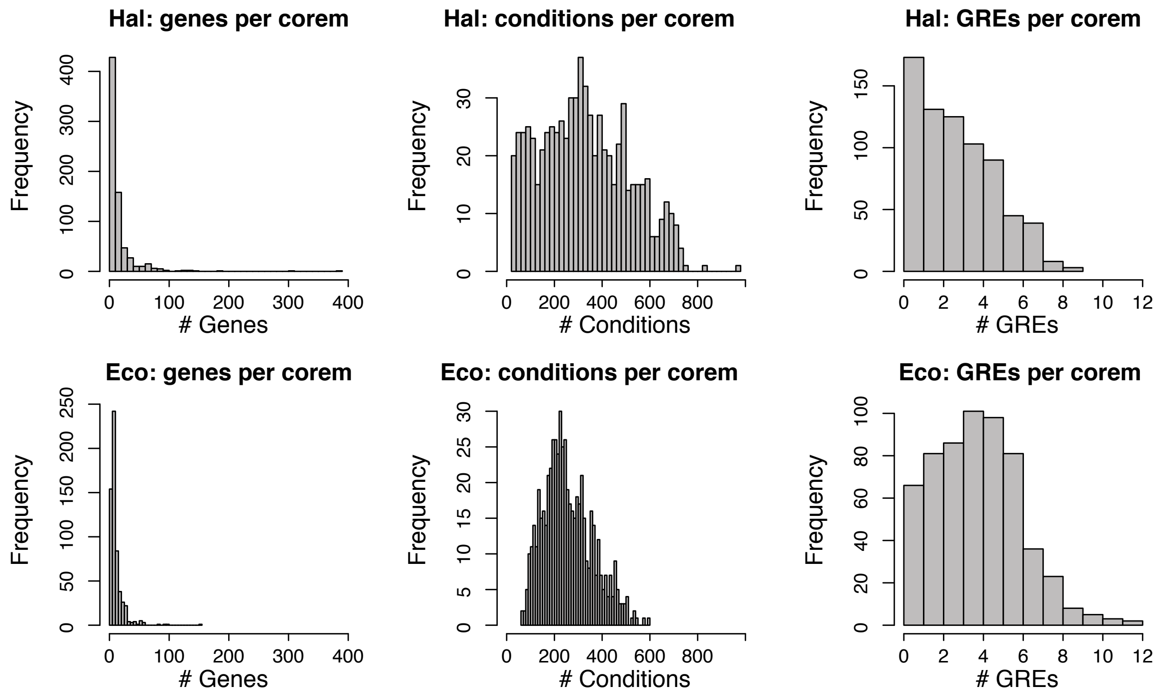

Figure S5: **Corem statistics.** Number of genes, conditions, and GREs per corem for *E. coli* and *H. salinarum* EGRIN 2.0 models.

4.5 Conditional co-regulation of genes organized in corems

We defined the conditions in which genes in a corem were co-regulated as the set of experiments in which the genes of a corem are more tightly co-expressed than one would expect at chance. We statistically evaluated tight

co-expression using relative standard deviation (RSD $= |\sigma/\mu|$) by resampling. We chose RSD (rather than, for example, standard deviation, σ) to avoid over-weighting conditions in which the mean relative expression is close to zero. The significance of an RSD value for a given condition relative to each corem was estimated by resampling: for a corem with k gene members, and for each condition, c, we computed at least 20,000 RSD values for k randomly sampled expression measurements in c, to determine the likelihood that the observed co-expression has lower RSD than expected by chance (p-value < 0.01). The resampling procedure resulted in condition sets for corems that contained from 1.4% to 85.5% of the conditions in *H. salinarium NRC-1* and 7.9% to 66.6% conditions in *E. coli K-12 MG1655* (Figure S5).

4.6 Conditionality of GRE influence

The upstream promoter regions of most genes contain multiple EGRIN 2.0-predicted GREs (*e.g.*, *carA* in Figure 2). A key insight of our model is that not all of these sites are equally important for controlling gene expression in all experimental conditions. We refer to changes in the relative influence of GREs across conditions as "conditional activity" of GRE elements. Although, to be clear, we do not imply that the transcriptional activity at a GRE is attributable to the DNA sequence itself, but rather the TF that binds to that sequence in particular environments. We leveraged the GREs discovered in genes grouped into corems and the conditional co-expression of those groups of genes to predict conditionally active GREs in EGRIN 2.0.

To identify the active GREs for each corem we combined predictions from (1) genome-wide motif scans (Section 4.3.4 above) that predict the GRE locations in an expanded region around each genes promoter in the corem using all of the ensemble predictions (1,000 nt window: -875 nt upstream to 125 nt downstream), and (2) the conditions discovered in biclusters that are most representative of the corem (*i.e.*, containing the largest fraction of genes from the corem, top decile). GREs that occurred frequently in these biclusters were considered putatively responsible for co-regulating the set of genes in the condition-specific context of the corem (q-value $\leq$ 0.05). Finally, we computed the average distances of all GREs to the start codons of each gene in the list (collapsing sites if they occurred within 25 nt of one another). The precise locations of all GREs for the *H. salinarum dpp* operon-related corems (Figure 3) are listed in Table E8, while the locations of GREs involved in conditional modulation of the PurR regulon (Figure 4) are provided in Table E9.

We represented the active GREs upstream of a gene or within a corem as a pie chart, showing the normalized frequency with which the GREs computed above occurred in biclusters containing that gene. For example, if GREs 1, 2, and 3 occurred in 25, 50, and 200 biclusters containing gene A, the pie chart for gene A would have sectors of area 0.09, 0.18, and 0.73 respectively. For corems, we computed the normalized frequency of GREs for all genes of the corem. For example, if GREs 1, 2, and 3 occurred in promoters of 10, 10, and 20 of the genes of the corem, their areas would be 0.25, 0.25, and 0.5 respectively.

4.7 Detection of conditional operons

Condition-specific transcriptional isoforms of operons were predicted through corem membership. If any of the genes in an operon were found in a corem that did not contain all the other genes of the operon, we predicted that the operon had conditional isoforms. Operon annotations for both *H. salinarum* and *E. coli* were derived from MicrobesOnline [2, 48]. All predicted conditional operons, including the specific break sites and transcriptional isoforms is available on the website. The full list of validated predictions is provided in Table E7.

4.8 Environmental ontology construction and usage

We recorded a rich set of meta-data for all 1,495 experiments conducted with *H. salinarum* and used for construction of the *H. salinarium NRC-1* EGRIN 2.0 model. The meta-data includes a detailed description of each experiment, including, for example: media composition, genetic background, concentration of perturbant, internal reference batch id, person who conducted the experiment, etc. We used this meta-information to classify experiments in an ontological framework, where two experiments can share specific meta-descriptions (*e.g.*, 10^{-3} mol/L EDTA),

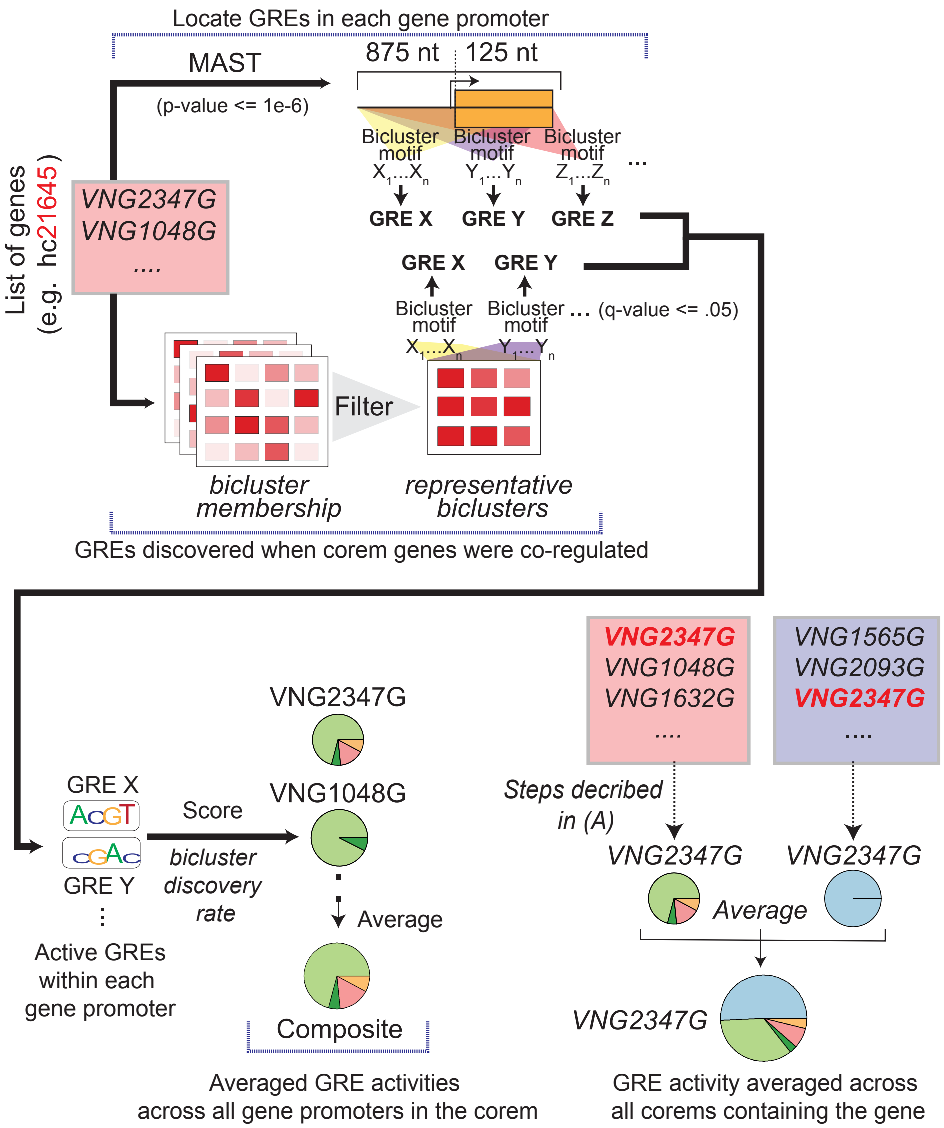

Figure S6: **Deciphering GREs responsible for regulating corems.** A GRE is implicated in regulation of a corem when it is both (1) located within an expanded region (-875nt to +125nt) around the translation start site of any gene in the corem; and (2) present in biclusters containing a large fraction of corem genes (top decile). Relative GRE influence is computed as the frequency with which each GRE was discovered in these representative biclusters (see Supplementary Methods for more details). Influence scores are illustrated as pie charts and reported for each gene individually (*e.g.*, *VNG2347G*); and as a composite by averaging across all genes in a corem. The width of each sector in the pie charts is proportional to the frequency of GRE discovery.

or inherit more general relationships from the ontological structure (*e.g.*, chemical perturbation). We used OBO-edit [17] to construct the ontology. The ontology contained 198 terms organized across three primary branches (environmental state, experimental state, and genetic state). The ontology flat file is available for download and meta-data annotations for every array in the dataset are available online.

We used the ontology to classify enriched environmental features for GREs and corems (Figures 3-4). For corems, we used the set of conditions in which genes in the corem are significantly co-expressed (see Section 4.5 above) to compute term enrichment using the ontoCAT [37] R-package. Term enrichment was assessed statistically and reported as q-values using the hypergeometric test with Benjamini-Hochberg correction for multiple hypothesis testing.

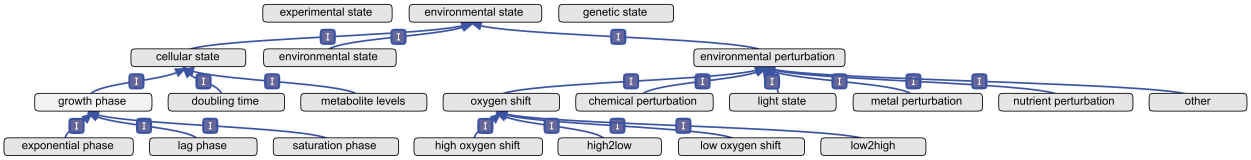

Figure S7: **Environmental ontology hierarchically organizes relationships between experimental conditions from metadata collected across 1495 experiments in *H. salinarum*.** Subset of the environmental ontology constructed for *H. salinarum* demonstrates many is-a (boxed I) relationships that organize similarities between descriptor terms descending from one of three root nodes (i.e., generic categorical descriptions). In this case a generic ontological term called 'environmental state' gives rise to much more specific terms (e.g., exponential phase or high oxygen shift) that inherit (at the highest level) a relationship through their being related to the environmental state of cells in the experiment. Each condition in the compendium is annotated with the most specific descriptors relevant to the experiment given metadata. The full environmental ontology is available for download from http://egrin2.systemsbiology.net.

5 Model validation

5.1 Global validation of gene regulatory elements predicted by EGRIN 2.0

We compared the genome-wide locations of predicted GREs in the *E. coli* EGRIN 2.0 model to experimentally mapped TF binding sites from RegulonDB (BindingSiteSet table, filtered for experimental evidence and TFs with ≥ 3 unique binding sites; a total of 88 TFs). We considered a GRE to be a significant match to a TF if a significant fraction (q-value ≤ 0.05) of its predicted non-coding locations overlapped with the known binding locations for a particular TF (hypergeometric p-value ≤ 0.01; see GRE definition in Section 4.3.3). In cases where a GRE significantly matched multiple TFs, only the most significant was reported.

We observed several instances where more than one GRE significantly matched the same TF. We were unable to determine whether this was the result of incomplete GRE clustering, ambiguities related to GRE scanning, limitations of the experimental data itself, or a reflection of subtle context-dependent variations in the binding preferences of these TFs. Since we did not observe clustering of GREs that map to the same TF upon re-clustering, we hypothesize that the observations may have biological origins, *i.e.*, reflect condition-dependent variations in TF binding preferences that are the result, for example, of co-activator/repressor interaction or small molecule binding. It is interesting to note that TFs with the largest fraction of GRE matches include transcriptional dual regulators, such as FlhDC and UlaR (*i.e.*, TFs with the ability to act as both activators and repressors). This is consistent with the observation that these TFs have context-dependent binding preferences. The complete set of validations, for both TFs and σ-factors, is listed in Table E4.

5.2 Global validation of regulatory interactions predicted by EGRIN 2.0

We assessed the ability of the EGRIN 2.0 model to correctly infer known regulatory interactions using the RegulonDB database as a standard metric for comparison. Comparison to the RegulonDB gold-standard is common practice for evaluating model performance [41]. We performed our evaluation with the version of RegulonDB used by the DREAM5 ensemble (based on RegulonDB release 6.8 [41]) so that we could directly compare our results. The authors [41] restricted the gold-standard to well-established interactions, annotated in RegulonDB with the 'strong evidence' classification. In all cases, networks were integrated from predictions among the ensemble using an approach similar to that of [41], with subtle variations noted in each section, below. To facilitate a direct comparison, we reconstructed a new *E. coli* EGRIN 2.0 model using the same DREAM5 expression consortium as was used for the original DREAM5 competition (Section 2.1.2.2). The predictions of this model were used *solely* for global validation and direct comparison with the DREAM5 community network, as described in this subsection.

We performed two global evaluations of the *E. coli* EGRIN 2.0: (1) a comparison of the GREs detected in the model with experimentally mapped TF binding sites in RegulonDB (Section 5.1), and (2) a comparison of

the predicted (TF $\rightarrow$ gene) regulation in EGRIN 2.0 with the gene regulatory network from [41]. For (2), we computed predicted regulatory networks from EGRIN 2.0 in two ways: (a) direct (TF $\rightarrow$ target) predictions from Inferelator (Section 5.2.1, and (b) a gene regulatory network derived from predicted GREs that were matched to TFs in RegulonDB (Section 5.2.2). Construction of each of these networks is described in detail below (Section 5.2.1 and Section 5.2.2). The methods for, and results of the comparisons are described in Section 5.2.4.

5.2.1 Conversion of EGRIN 2.0 Inferelator influence predictions into a GRN

We computed a direct (TF $\rightarrow$ gene) inferred *E. coli* gene regulatory network (GRN) from the Inferelator predictions in the EGRIN 2.0 ensemble. As with the original EGRIN model [9], Inferelator influence predictions were originally made between the 296 putative *E. coli* TFs (Section 2.2.3.2) and each of the $\sim 40,000$ biclusters in the ensemble. We then used a weighted average of the predicted influences among all networks in the ensemble, as follows. If Inferelator predicted a (TF $\rightarrow$ bicluster) influence with weight β then we added β to a regulatory interaction between that TF and all genes in that bicluster. Weights β were summed for each recurrence of the same (TF $\rightarrow$ gene) interaction. Note, we did not use $|\beta|$ in the individual sums, since we considered contradicting evidence to be cancelling rather than reinforcing. Finally, all (TF $\rightarrow$ gene) interactions in the final network were ranked by absolute total weight (here we *did* use $|\beta|$). As with the DREAM5 competition networks, the top 100,000 rankings were retained in the final network. The final EGRIN 2.0 Inferelator influence network is available online.

5.2.2 Conversion of EGRIN 2.0 GRE detections into a predicted GRN

We computed a separate inferred *E. coli* gene regulatory network from predicted GREs in EGRIN 2.0 that were matched to TFs as described in Section 5.1. We would like to stress that this inference relies upon (in this case, for *E. coli*) annotated binding sites for regulators, which could be statistically linked to predicted GREs through significant overlaps in their genomic locations. This enables inference of (TF $\rightarrow$ gene) direct influence predictions through the indirect relationship:

$$\mathrm{TF} \overset{\text{anno.}}{\rightarrow} \mathrm{GRE} \overset{\text{pred.}}{\rightarrow} \mathrm{gene}. \tag{11}$$

Thus for an understudied organism, such as *H. salinarum*, such a network of (TF $\rightarrow$ gene) influences could *not* be inferred; rather a (GRE $\rightarrow$ gene) interaction network would be the final product. Such a network still contains predictions which could be validated and acted upon, for example, for engineering purposes. A future direction of our research will be to statistically link TFs to predicted GREs, for example using direct GRN predictions such as those described above (*e.g.* Section 5.2.1, or [41]).

(GRE $\rightarrow$ gene) predictions (in Eq. 11) were extracted from the EGRIN 2.0 model directly using the MEME predictions for motif instances in the promoters of genes in each of the ~40,000 cMonkey biclusters. We then used an unweighted average of the predictions among all bicluster in the ensemble, as follows. A (TF $\rightarrow$ gene) edge with a weight of 1 was added to the predicted network if the annotated binding sites for that TF could be matched with locations of a motif (Section 5.1), which was detected by MEME in a bicluster in the promoter of the gene. Edge weights (1) were added for each additional prediction, in the ensemble of biclusters, of the same (TF $\rightarrow$ gene) interaction. As with the Inferelator influence network (Section 5.2.1), the top 100,000 rankings were retained in the final network. The final EGRIN 2.0 GRE-based network is available online.

5.2.3 Integration of predicted EGRIN 2.0 Inferelator- and GRE-based GRNs

Prior to integration of the two different predicted GRNs described above (Sections 5.2.1 and 5.2.2), we ensured that they were both equally represented in the integrated GRN by re-scaling their weights so that their sums would be equal. The GRNs were then combined into a single, integrated predicted EGRIN 2.0 GRN by simply summing the re-scaled weights for any edge predicted in both networks. Thus, this final network integration was a form of weighted average of the two (GRE and Inferelator) networks. This is *not* identical to the weighted rank average method described by [41], as it does not use a posteriori assessments of each network to assign their relative weights; rather the weights are simply adjust so that each network contributes equally to the predictions.

5.2.4 Network comparisons and global performance assessments

To compare EGRIN 2.0 performance to the DREAM5 ensemble, we computed standard precision-recall statistics for each network using the previously described DREAM5 gold standard GRN. We computed area-under-the-precision-recall (AUPR) statistics to summarize the predictive performance. AUPR statistics were compared directly with the DREAM5 community ensemble network. By extension, the EGRIN 2.0 AUPR performance can be compared to the individual best performers in DREAM5 as well (Figure 2A in [41]). The results of these analyses are summarized in Figure 2A in the main text. We have made all network predictions available online. Complete precision-recall curves are shown in Figure S8. The curves are also available in tabular form online.

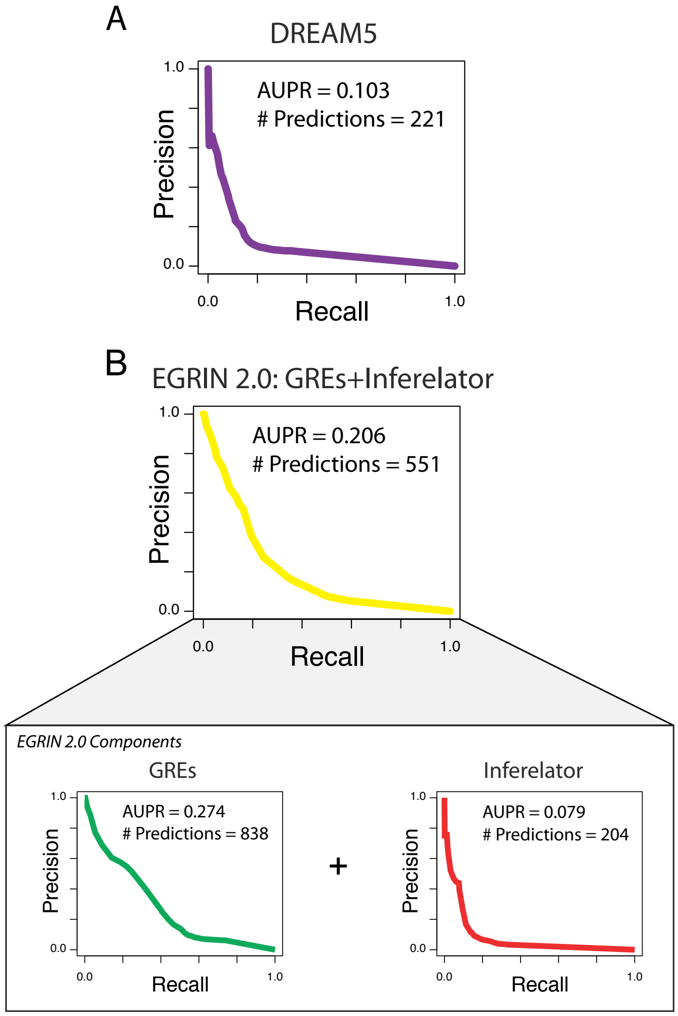

Figure S8: **Precision-recall performance for *E. coli* networks.** Comparison of precision-recall performance on *E. coli* RegulonDB gold-standard (Section 3.2.6), for the DREAM5 ensemble network (A), compared to EGRIN 2.0(B). We compare the GRE-based and Inferelator-based networks (bottom)to the integrated EGRIN 2.0 network (top). The integrated EGRIN 2.0 network consists of an equal weighting of the GRE-based and Inferelator-based networks. The EGRIN 2.0 networks were inferred using the DREAM5 mRNA expression compendium (Section 2.1.2.2). Area under the curve (AUPR) and the number of true-positive predictions at a precision of 25% are listed for each curve.

We further investigated the convergence of the AUPR statistics for each of the EGRIN 2.0-predicted regulatory networks as additional individual EGRIN models are added to the ensemble. This assessment helps to address the question of whether the approach utilized for ensemble integration has the desired property of performing better than most (if not all) of the individual models. Additionally, it can address the question of how many individual EGRIN models are necessary to achieve a given performance level. We observed that this is indeed the case for the Inferelator-based predictions extracted from the EGRIN 2.0 model (Figure S9a), whose final AUPR of 8.5%

far exceeds the rather poor performance of all 106 individual component EGRIN models (with an average AUPR of 5.0% and a maximum of 7.4%). The performance of the ensemble for this measure converges rather quickly to the final measure, after roughly 50 of the 106 EGRIN models are integrated (taking into account the variance in models observed with integrating the models in different orders). For the EGRIN 2.0 GRE-based predicted network (Figure S9b), ensemble surpasses 84 (79%) of the 106 individual component EGRIN models. This measure continues to improve until ~ 80 of the 106 models are integrated, suggesting that for this data set (the DREAM5 *E. coli* expression compendium), ~ 100 EGRIN models was a reasonable number to use in construction of the EGRIN 2.0 ensemble.

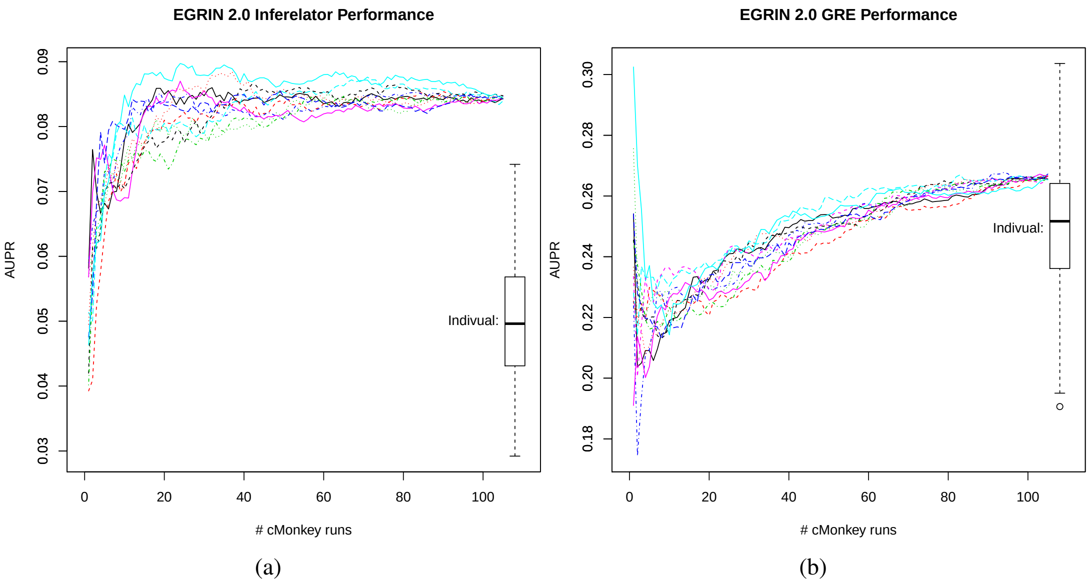

Figure S9: **Ensemble performance of individual GRN predictions.** EGRIN 2.0-inferred *E. coli* regulatory network predictive performance (AUPR vs. *E. coli* DREAM5 [41] gold standard) for Inferelator-based predictions (a) and GRE-based predictions (b) from EGRIN 2.0. Shown for both networks is the cumulative AUPR as each of the 106 individual model components is integrated in to the ensemble (as described in Section 5.2). Lines showing the cumulative AUPR for randomized orderings of the components' integration into the ensemble reveal the slight variations in performance that could be observed, and that these converge prior to integration of the final (106th) component. Also included for comparison is a box-whisker plot which shows the distribution of corresponding AUPR scores for the 106 individual EGRIN models.

Figure S10 shows the inferred networks for two genes regulated by PurR and ArgR (comparing predictions from EGRIN 2.0, CLR, DREAM5, and RegPrecise to the annotations in RegulonDB). The result demonstrates that GRE-based approaches can discover interactions that are not predicted using direct approaches (See Section 5.2.2).

5.3 Validation of condition-specific operon isoforms by tiling array transcriptome measurements

We validated the prevalence of multiple, condition-specific transcriptional isoforms from operons in *E. coli K-12 MG1655* by measuring changes in the transcriptome across growth, from lag-phase (OD600 = 0.05) to late stationary phase (OD600 = 7.3). The experimental platform and other experimental details are described in Section 3.2.1. We used multivariate recursive partitioning, including signals from both relative changes in expression along the growth curve, as well as raw RNA hybridization signal to call putative transcription breaks as previously described [35]. To determine the significance of our finding, we computed a p-value describing the significance of the overlap between our predictions (see Section 4.7) and the experimental observations using the cumulative hypergeometric distribution.

Figures S11, S12, and S13 below depict several operons annotated with condition-specific transcriptional isoforms. We have integrated GRE elements discovered near break sites with the transcriptional measurements.

5.4 Gene-gene co-fitness correlations in regulatory modules

To assess the phenotypic consequences of co-regulation in corems, we assessed whether genes grouped into corems had significantly similar fitness consequences in many environments (*i.e.*, the effect of deleting one gene is highly similar to the effect of deleting the other across many environments). We used the high-throughput fitness screen described in Section 3.2.3 to quantify these relationships.

We compared the enrichment for high co-fitness relationships in corems to other ways of assigning co-regulatory modules, including regulons (RegPrecise, RegulonDB), operons, and WGCNA. The gene modules for regulons (annotated in RegulonDB or RegPrecise [45]) consisted of genes annotated to a common TF. For WGCNA, we assigned modules using the same community detection procedures that we used to define corems from the EGRIN 2.0 ensemble (See 4.3.5.1). The gene co-expression modules were computed from the weighted WGCNA adjacency matrix.

For the results presented in Figure 2B, we compared the distributions of Pearson correlations between relative changes in fitness across pairs of genes within each module, using the one-tailed Kolmogorov-Smirnov test (KS-test). We report the KS D-statistic. The precision/recall characteristics for each model are contained in Table E5.

We extended this analysis by investigating whether the enriched high co-fitness gene-gene relationships in corems consist of relationships that could be described fully by regulons or operons. To answer this question, we removed all gene pairs from corems that are also present in operons or regulons and computed the KS-test again (Figure S14). We still observe a significant number of high co-fitness relationships, suggesting that corems capture physiologically meaningful co-regulatory relationships between genes that cannot be explained by existing paradigms.

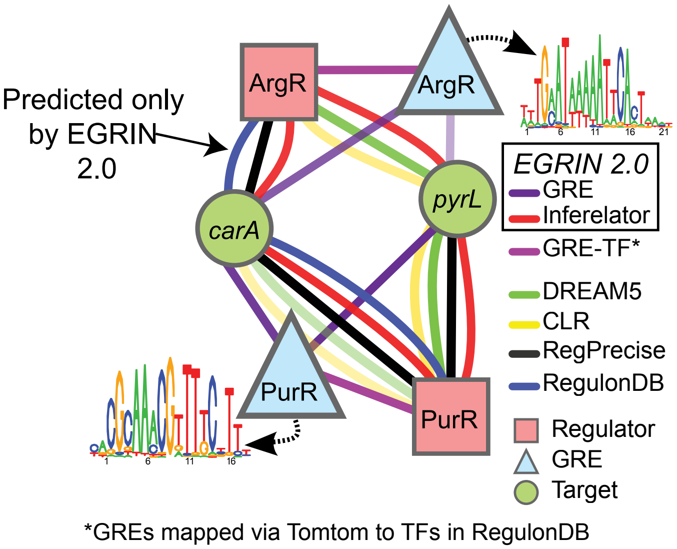

Figure S10: **Integration of GRE discovery and Inferelator predictions yields comprehensive and detailed gene regulatory networks.** EGRIN 2.0-inferred *E. coli* regulatory subnetwork for two genes (green circles) in the PurR/ArgR regulon: *carA* (*b0032*) and *pyrL* (*b4246*). The EGRIN 2.0 predictions are divided into GRE-based (dark violet) and Inferelator-based (red), and compared to predictions (or annotations) from other algorithms/databases (yellow: CLR; green: DREAM5 ensemble; black: RegPrecise; blue: RegulonDB). In two cases (ArgR→carA and ArgR→pyrL), EGRIN 2.0 discovers regulatory interactions that were missed by either hand-curated databases or expression-based inference procedures.

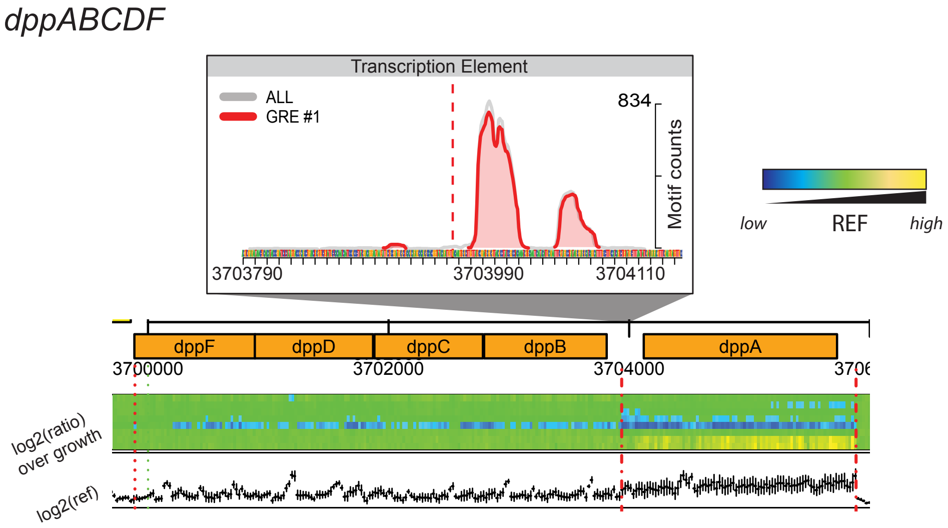

Figure S11: **GREs regulate multiple transcript isoforms from operons in** ***E. coli*****,** ***dppABCDF*****.** GREs coincide with experimentally measured break sites. Three examples of experimentally determined transcription break sites (red dashed lines) in operons predicted by corems to be conditionally segmented. Expression levels of these regions were profiled across growth in rich media (heatmap). Inset contains region immediately surrounding a transcriptional break site, including counts of GREs discovered at these locations (as in Figure S19).

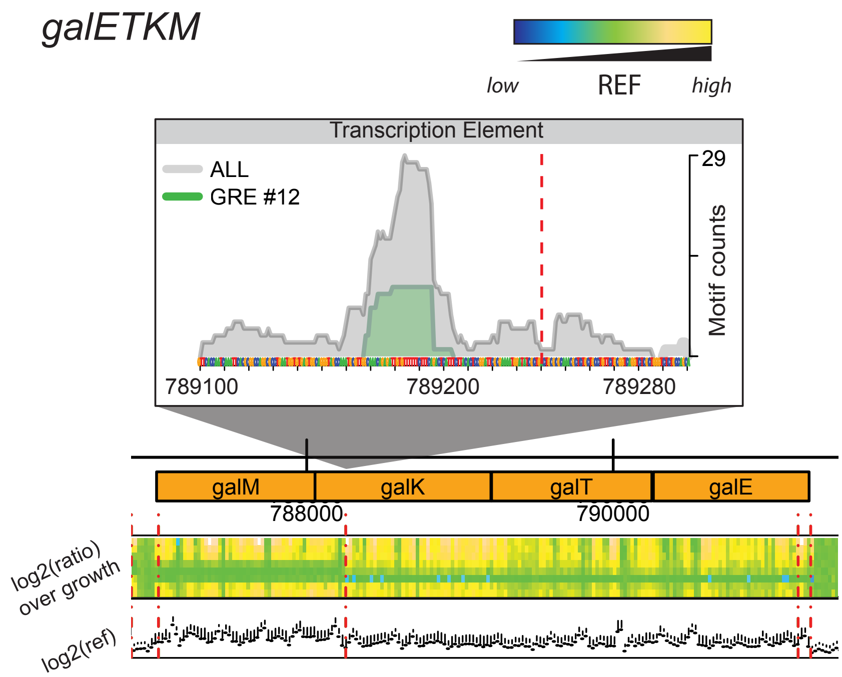

Figure S12: **GREs regulate multiple transcript isoforms from operons in** *E. coli*, ***galETKM*.** Caption details included in Figure S11.

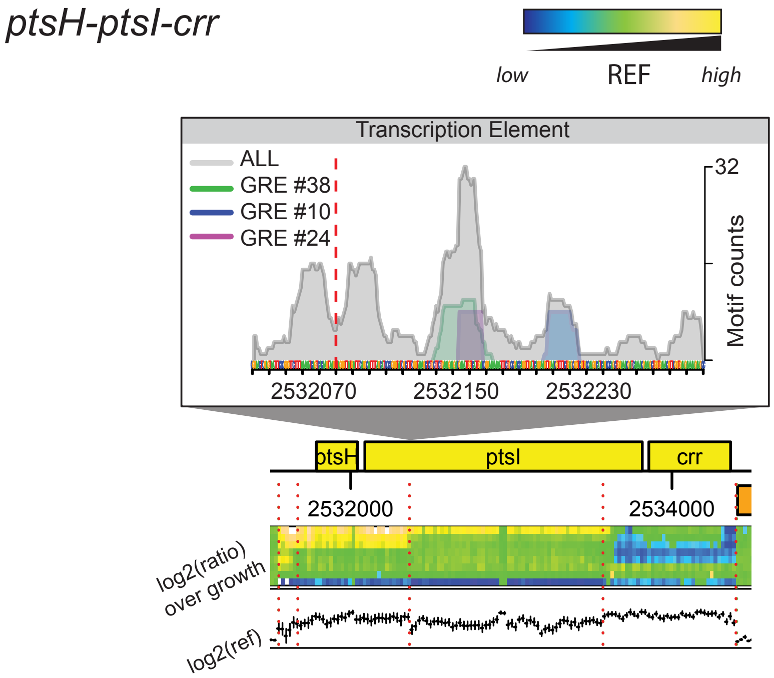

Figure S13: **GREs regulate multiple transcript isoforms from operons in** *E. coli*, ***ptsH-ptsI-crr*.** Caption details included in Figure S11.

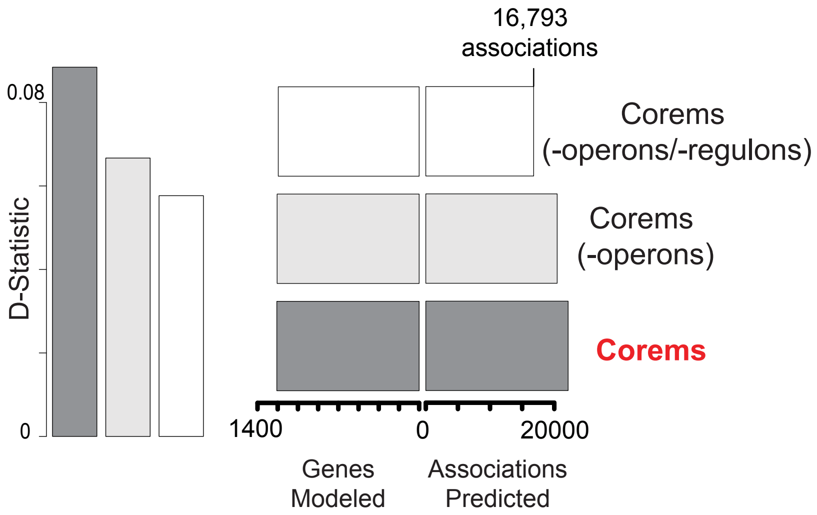

Figure S14: **EGRIN 2.0 models highly correlated co-fitness relationships that cannot be explained by operons or regulons.** (Left) Enrichment for highly correlated, pairwise fitness measurements in gene knock outs across 324 conditions before and after removing gene associations annotated by operons (Microbes Online) and regulons (RegulonDB and RegPrecise) (KS-test,D-statistic). Two-thirds of gene-pairs with most highly correlated fitness within corems are not annotated by operons or regulons. (Right) Number of genes and associations predicted.

6 Model evaluation

In this section we evaluate the performance of the EGRIN 2.0 model as a function of several important parameters. We focus in particular on how the performance of the model changes as a function of the number of runs included. From these evaluations, we conclude that (1) the model performs well in its final form, (2) the model has reached a stable-state wherein inclusion of additional runs does not significantly increase model performance, and (3) the model is not over-fit to particular experiments within a data set or to any data set as a whole.

6.1 Comparison with other module detection algorithms

We compared the number of RegulonDB TFs detected in the EGRIN 2.0 model to individual cMonkey runs as well as to several other module detection/clustering algorithms that were computed on subsets of the experimental data (similar to the EGRIN 2.0 ensemble; Figure S15). We evaluated: (a) k-means clustering, (b) WGCNA [38], and (c) DISTILLER [40]. For (a) and (b), we computed modules 100 times on random subsets of the *E. coli* expression data set (using 200-250 randomly chosen experiments per run; selection criteria were identical to *E. coli* EGRIN 2.0; see Table S2). We then predicted *de novo cis*-regulatory GREs in the promoter regions of genes in each module using MEME (MEME parameters were also identical to EGRIN 2.0; Table S2). For (c), we performed the comparison using the original modules generated by [40]. Rather than alter module composition by re-detection, we instead varied MEME parameters applied to the modules 100 times (again, within the same ranges as those used for EGRIN 2.0). TF-GRE matches were assigned by comparing GREs to RegulonDB TF binding sites, as previously described (Section 5.1).

We found that individual cMonkey runs discovered a greater number of RegulonDB binding sites, on average, than the other methods (an average of 41 for cMonkey, compared to averages of 30, 25, and 29 for k-means, WGCNA, and DISTILLER, respectively), which is consistent with previous findings [50] (Figure S15). Integration of all cMonkey biclusters into the complete EGRIN 2.0 ensemble outperformed all individual cMonkey runs (53 total, as described in the Manuscript). This result is typical of ensemble-based inference approaches, and supports the value of ensemble integration as part of the EGRIN 2.0 model.

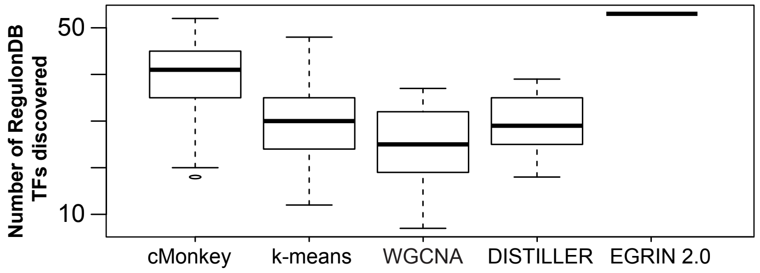

Figure S15: **Number of TFs in RegulonDB re-discovered by various regulatory module detection methods.** Comparison of EGRIN 2.0 (solid line, far right) to individual cMonkey runs, as well as multiple runs of k-means, WGCNA, and DISTILLER on subsets of the expression data. Evaluation made with respect to re-discovery of binding sites for 88 TFs with ≥ 3 unique sites in RegulonDB based on genome-wide binding site locations (FDR ≤ 0.05).

6.2 Convergence and stability of the inferred network

To evaluate the stability of the inferred EGRIN 2.0 network, we quantified how the model changes as individual cMonkey runs are excluded from the ensemble. Since the sub-bagging, as performed for the EGRIN 2.0 model inference, reduce model over-fitting, we used this evaluation understand whether the model is over-fit to particular experiments in the data set. For this task, we computed the number of individual EGRIN runs required to converge on a consistent gene-gene co-occurrence network (see Section 4.3.5.1). We computed gene-gene co-occurrence networks based upon randomly selected subsets of the 106 available *E. coli K-12 MG1655* cMonkey runs, and

varied the percentage selected between 1%-99% of the 106 runs. 5 replicate samples were computed for each. To compare the networks, we computed the Pearson correlation between the two matrices (sub-sampled gene-gene co-occurrence versus the final EGRIN 2.0 gene-gene co-occurrence network). Note that since the gene-gene co-occurrence network is a weighted adjacency matrix, the correlation reflects the weighted discovery rate for every pair of genes (rather than simple presence/absence). In Figure S16 we demonstrate that the underlying networks converge rapidly to the final solution. By the time $\sim$ 50% of the runs have been included ($\sim$ 50 runs), the inferred network is nearly identical to the final network ($\sim$ 100 runs; cor $>$ 0.9). The backbone extracted network takes a slightly longer time to converge, likely because it requires more observations of gene-gene pairs to retain them in the final network. Since corem detection is deterministic and strictly based on the underlying gene-gene co-occurrence matrix, this convergence means that the inferred corems would be nearly identical even if up to half of the runs were excluded.

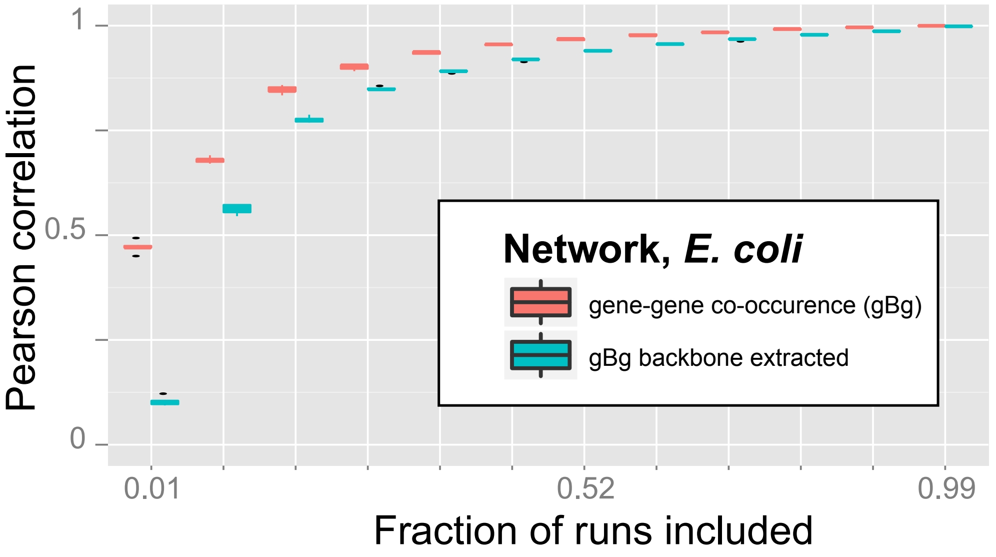

Figure S16: **Convergence of EGRIN 2.0 co-occurrence networks.** The co-regulation of genes predicted by the *E. coli K-12 MG1655* EGRIN 2.0 model converges rapidly to a stable network. Shown is the similarity of the gene-gene co-occurrence matrix (and the backbone extraction of this matrix) to the final EGRIN 2.0 *E. coli K-12 MG1655* network, computed when varying fractions of the cMonkey runs were excluded (Pearson correlation vs. the complete model). Each point contains a box plot representing 5 replicate sub-samples.

6.3 Discovery of corems in an independent data set

To determine whether EGRIN 2.0 model predictions are over-fit to the DISTILLER expression compendium (or are the result of biases in that data set), we tested whether support for corems existed in an independent *E. coli* expression data set. Such evidence would suggest that corems are *bona fide* gene regulatory modules that can be re-discovered in independent data, and that their degree of condition-specificity is not biased due to normalization differences in any given data set. For this test, we used the DREAM5 gene expression compendium. As described above (Section 2.1.2.2), this data set is comprised of different conditions, array platforms, and, most important, was normalized by different methods, than the DISTILLER data set used for model training. We determined the condition-specific activity of corems in the DREAM5 data set using the methods described in Section 4.5. If a corem was significantly co-expressed (p-value $\leq$ 0.05) in at least one condition, we classified it 'supported'. To our surprise, we not only discovered support for $\sim$ 99% of the predicted corems, we also discovered that their conditionality was very similar across both data sets – *i.e.*, corems discovered to be co-expressed in few conditions in the DISTILLER data set are also co-expressed in few conditions in the DREAM5 data set (same for corems regulated in many conditions), and similarly for corems co-expressed in a large number of conditions (Figure S17). Even after we removed the intrinsic relationship between the number of genes in a corem and the number of conditions in which it is co-expressed, we

still observed a significant partial correlation of 0.49 (p-value $< 10^{-6}$) between the number of conditions in corems as defined from the two data sets.

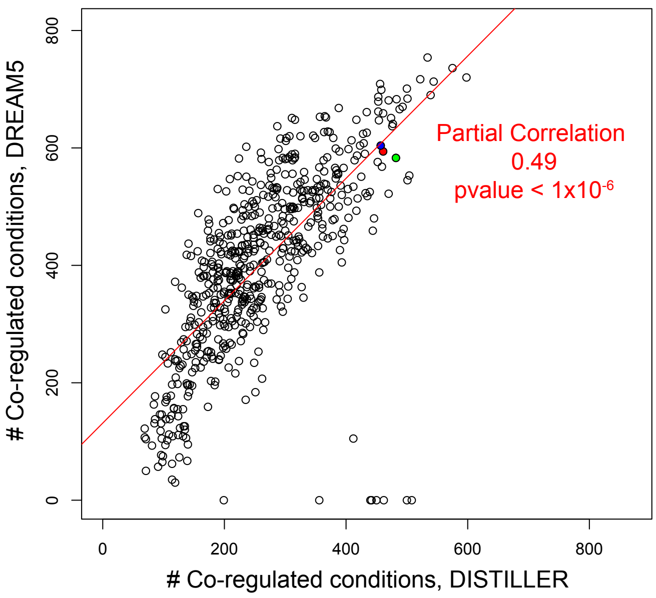

Figure S17: **Reproducibility of corems across data sets.** Number of co-expressed conditions for corems in the DISTILLER and DREAM5 expression compendia. Conditions were selected as in Section 4.5. Significant partial correlation of 0.49 is observed after removing the affect of gene set size (log) on the number of conditions co-expressed (p-value $< 10^{-6}$). The three corems detailed in the main manuscript are identified with their respective colors (ec512157, ec516034, ec516031)

7 Additional Supporting Figures Referenced From Main Text

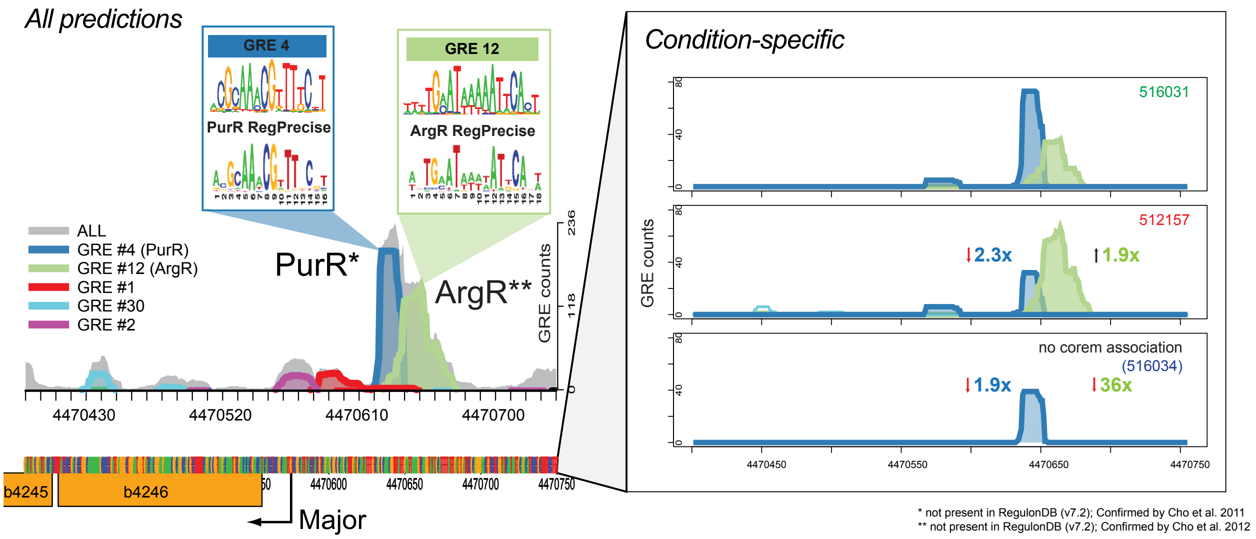

Figure S18: **Differential GRE activity in *pyrL* promoter, *E. coli*.** (Left) Predicted promoter architecture for *E. coli pyrL* (*b4246*). Overlapping GREs matching to PurR (GRE #4) and ArgR (GRE #12) were detected upstream of pyrL. These sites were not annotated in RegulonDB, but were validated in independent ChIP-chip experiments [14, 15]. Transcription start site indicated with arrow. (Bottom) Condition-specific promoter architectures for *E. coli pyrL* (as in Figure 2E). Variation in predicted GRE activity across three different subsets of experimental conditions (counts and fold-change) for two GREs in the *pyrL* promoter. Experimental subsets correspond to conditions under which at least one of three nucleotide biosynthetic corems is regulated (denoted by colored names at top-right of each plot)

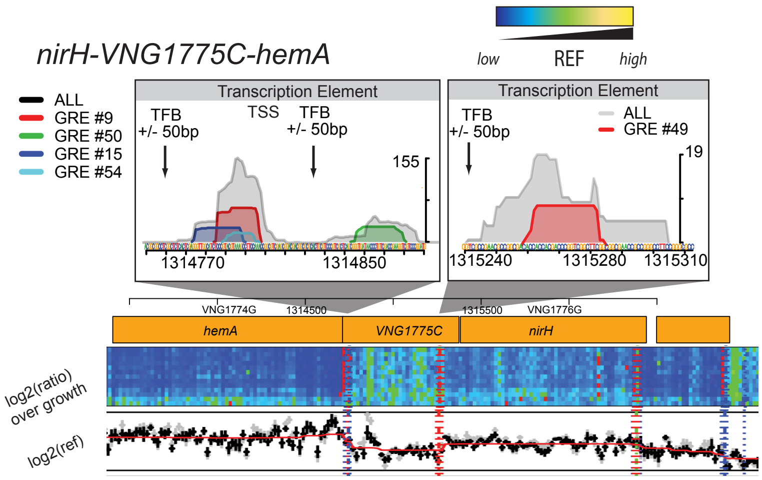

Figure S19: **GREs regulate multiple transcript isoforms from operons in** *H. salinarum*, ***nirH-VNG1775C-hemA*.** GREs located inside operons coincide with experimentally measured transcriptional break sites. Experimentally determined transcription break sites (red dashed lines) above expression profiles of these regions across growth (heatmap, [35] and ChIP-chip TFBs ([22], vertical arrows) support the role of GREs in regulating segmentation of the operon in certain conditions. Insets contain regions immediately surrounding transcriptional break sites, including counts of GREs discovered at these locations.

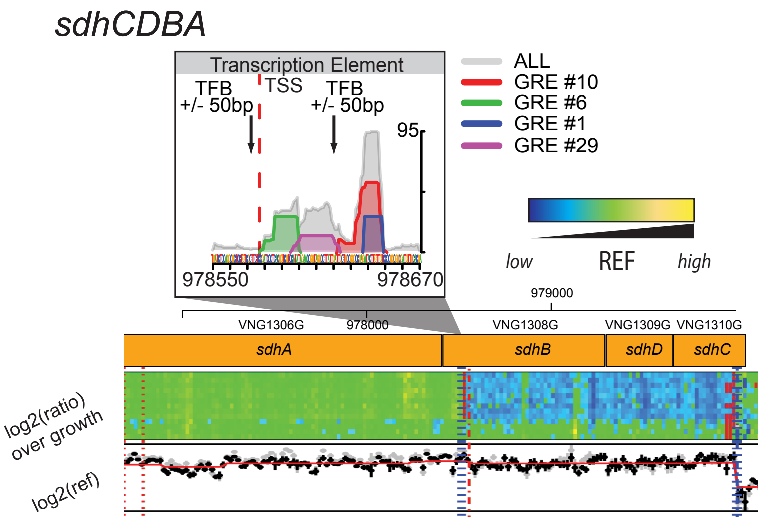

Figure S20: **GREs regulate multiple transcript isoforms from operons in** *H. salinarum*, ***sdhCDBA*.** Caption details included in Figure S19

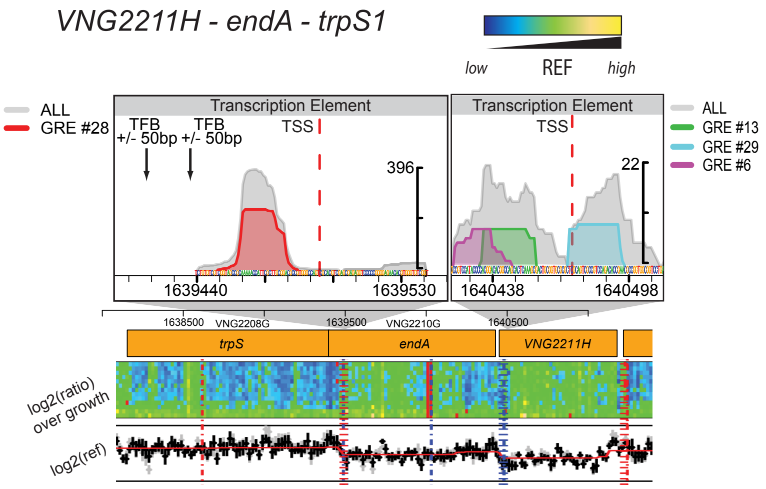

Figure S21: **GREs regulate multiple transcript isoforms from operons in** *H. salinarum*, ***VNG2211H-endA-trpS1*.** Caption details included in Figure S19

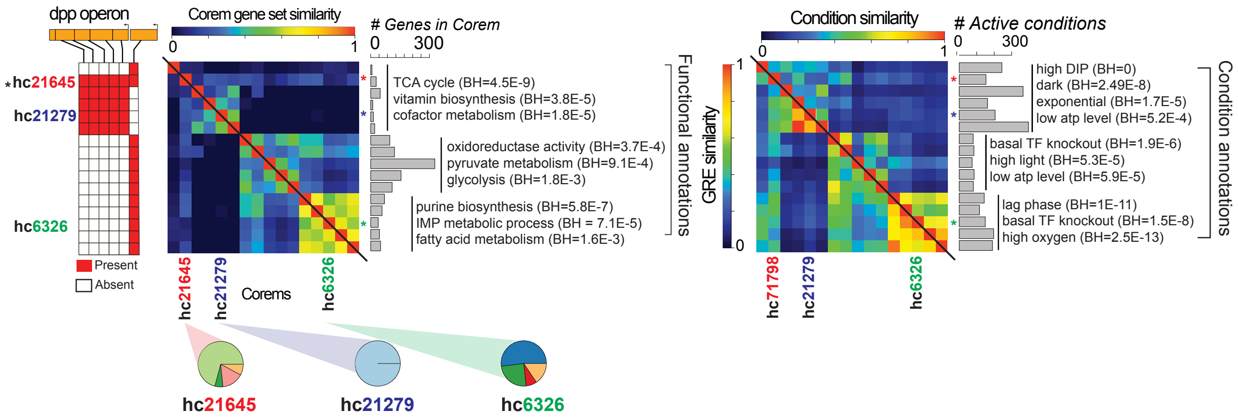

Figure S22: **Alternate regulatory modes for *dpp* operon predicted by corems.** Corems group together functionally related sets of genes that are co-regulated in similar environments by similar factors (Left) Presence/absence of *dpp* operon genes in corems. Three classes of corems exist for the dpp operon: (1) the entire operon (*e.g.*hc21645), (2) the leader gene *dppA* (*e.g.*hc6326), and (3) five "tail" genes excluding *dppA* (hc21279). (Middle) Gene similarity between corems (heatmap, Jaccard index). Functional annotations of genes in three highly similar clusters of corems to right. GRE composition for three corems shown below (pie chart, see Figure S6). (Right) Similarity of conditions regulated (heatmap, upper triangle, Jaccard index) and GREs (heatmap, lower triangle, Jaccard index) among corems. Ordering is identical to (Middle). Environmental Ontology term enrichment (see ref) for three clusters depicted to right.

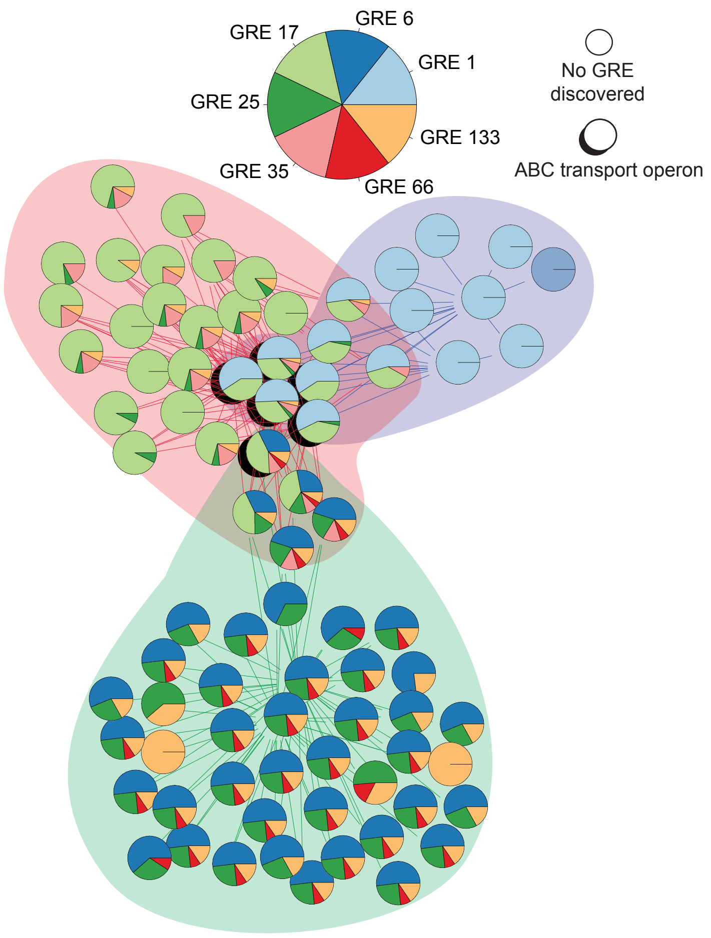

Figure S23: **Network representation of transcriptional isoforms for the *dpp* operon predicted by corems.** Network representation for three corems described in S22. Genes represented by circles. Edge colors and colored region behind the network indicate corem membership. Pie charts reflect GRE composition of each gene (see Figure S6). Key for pie charts at top. Shading behind nodes (center of network) indicates *dpp* operon genes.

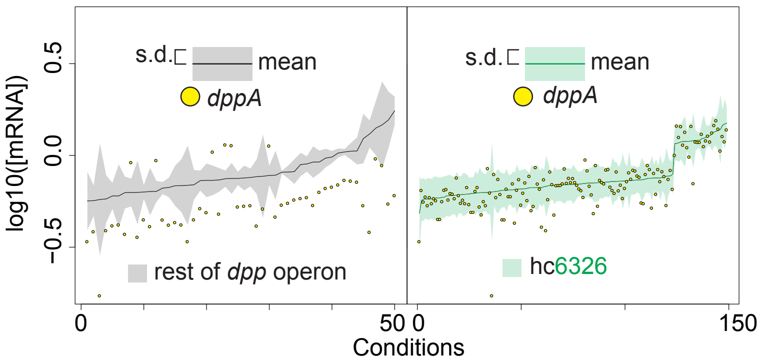

Figure S24: ***dppA* is more tightly co-expressed with genes of hc6326 in some environments than the other genes in the *dpp* operon.** Relative expression of *dppA* compared to (left) other genes of *dpp* operon and (right) hc6326.

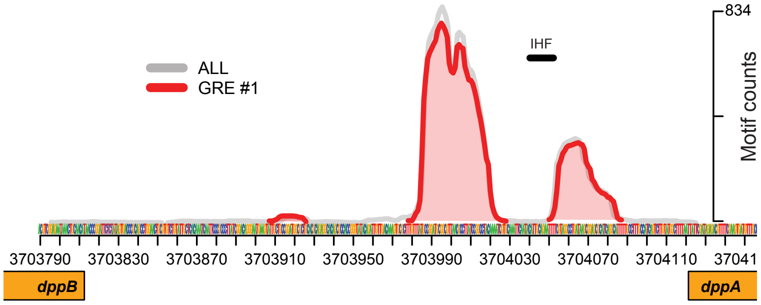

Figure S25: **Evidence for condition-specific transcript isoforms of the *dpp* operon in *E. coli*.** EGRIN 2.0 predicts conditional modulation of *dpp* operon in *E. coli* as well. Promoter architecture within intergenic space between *dppA* and *dppB* suggested locations for TF binding internal to the operon (as in Figure 3A). GRE binding sites are proximal to an experimentally characterized IHF binding site (black horizontal bar; RegulonDB).

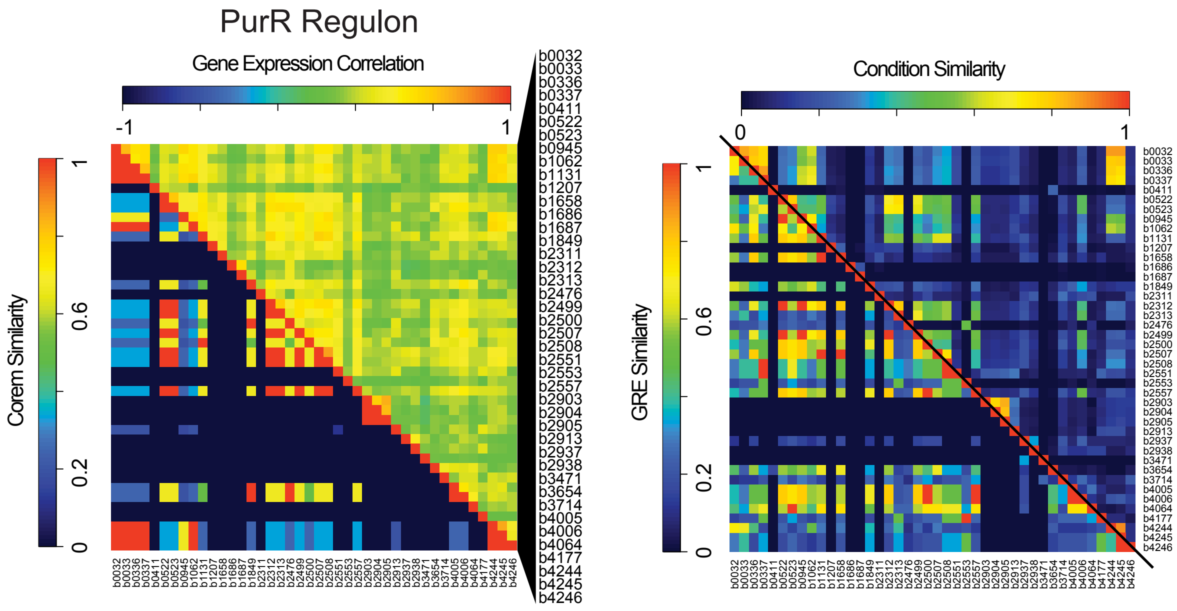

Figure S26: **Corems model the mechanistic basis for conditional subdivision of the PurR regulon, *E. coli*.** (Left) Corems identify the most highly correlated subgroupings of genes in PurR regulon. Gene expression correlation across all experiments (upper triangle) compared to similarity of corem membership (lower-triangle, Jaccard index) for genes of the PurR regulon (gene identifiers expanded to right). (Right) Similarity of regulated conditions (upper triangle, Jaccard index) and GREs composition for these genes (bottom triangle, Jaccard index). Consistent patterns of conditional-activity and GRE composition in their promoter regions further supports subdivision of PurR genes into separate corems. Gene order is same as left.

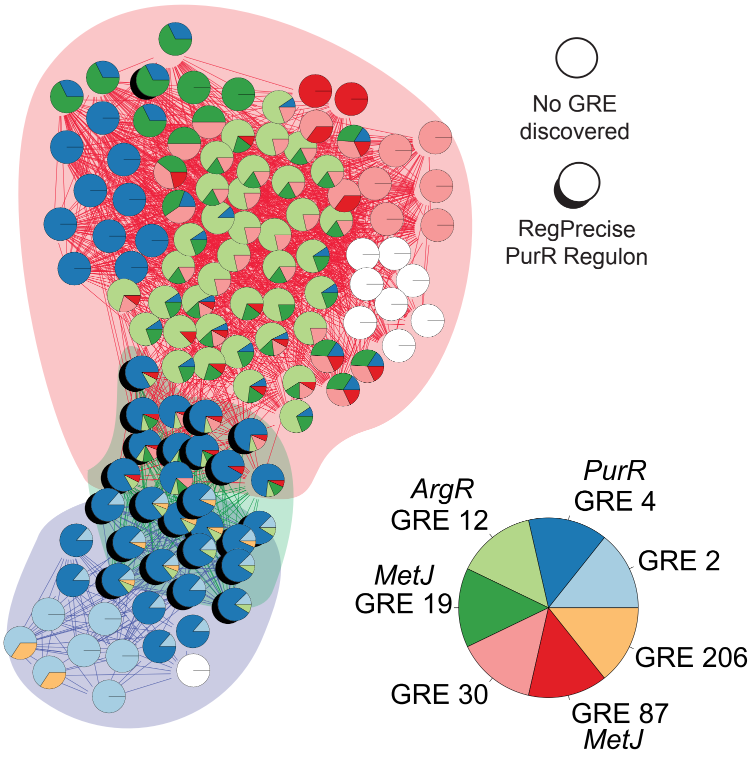

Figure S27: **Corems integrate diverse regulatory mechanisms, *E. coli*.** Network representation for three corems described in Figure S26. Genes are represented by circles. Edge colors and colored region behind the network indicate corem membership. Pie charts reflect GRE composition of each gene (see Figure S6). Key for pie charts at bottom. GRE-TF matches are indicated. Shading behind nodes denotes PurR regulon genes. At least 7 different mechanisms regulate the expression of these genes.

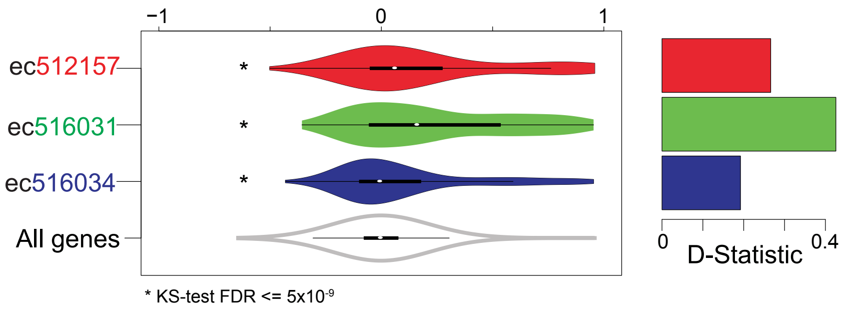

Figure S28: **Genes from corems related to nucleotide biosynthesis have highly similar fitness effects when they are deleted.** (Left) Violin plot shows distribution of all fitness correlations for genes in three nucleotide biosynthesis-associated corems compared to all genes in the data set. (Right) KS D-Statistic relates to enrichment for highly correlated gene-gene fitness associations in the corems. All three corems enrich for similar fitness effects (KS FDR $< 5 \times 10^{-9}$)

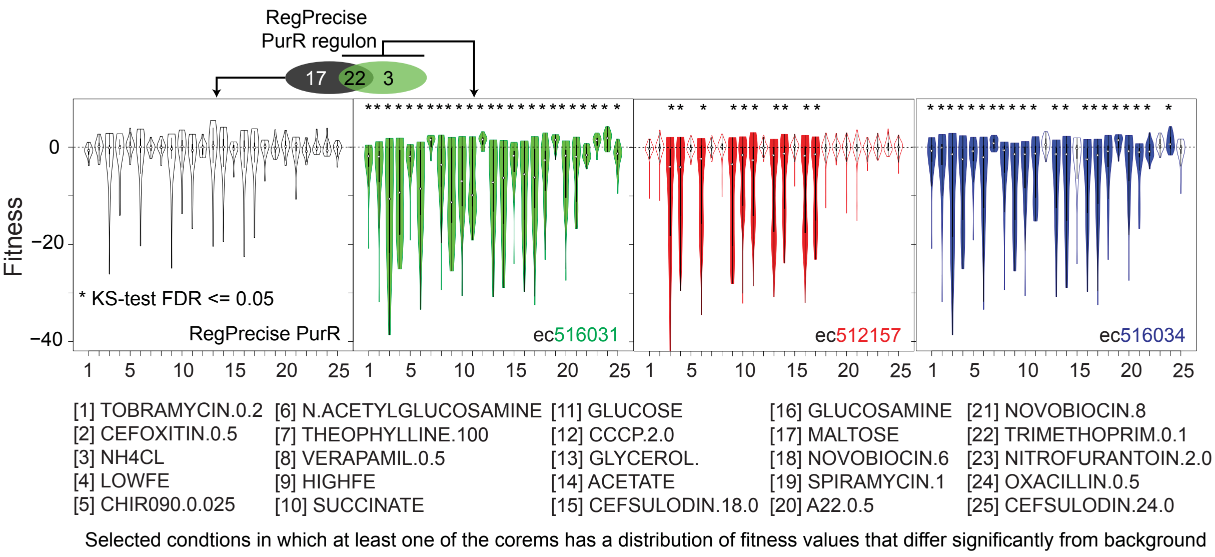

Figure S29: **Corems model fitness effects that occur in specific environments.** Violin plots show distribution of relative fitness among corems across conditions (negative values indicate lower fitness relative to WT). Brief condition descriptions are displayed below. Shading within the violin plot indicates that the distribution of fitness values is significantly in that condition (KS-test FDR ≤ 0.05). Fitness values for the subset of genes from the PurR regulon that do not occur in ec516031 are displayed to the left. These genes do not have significant fitness effects in any of the environments tested. Data from [44].

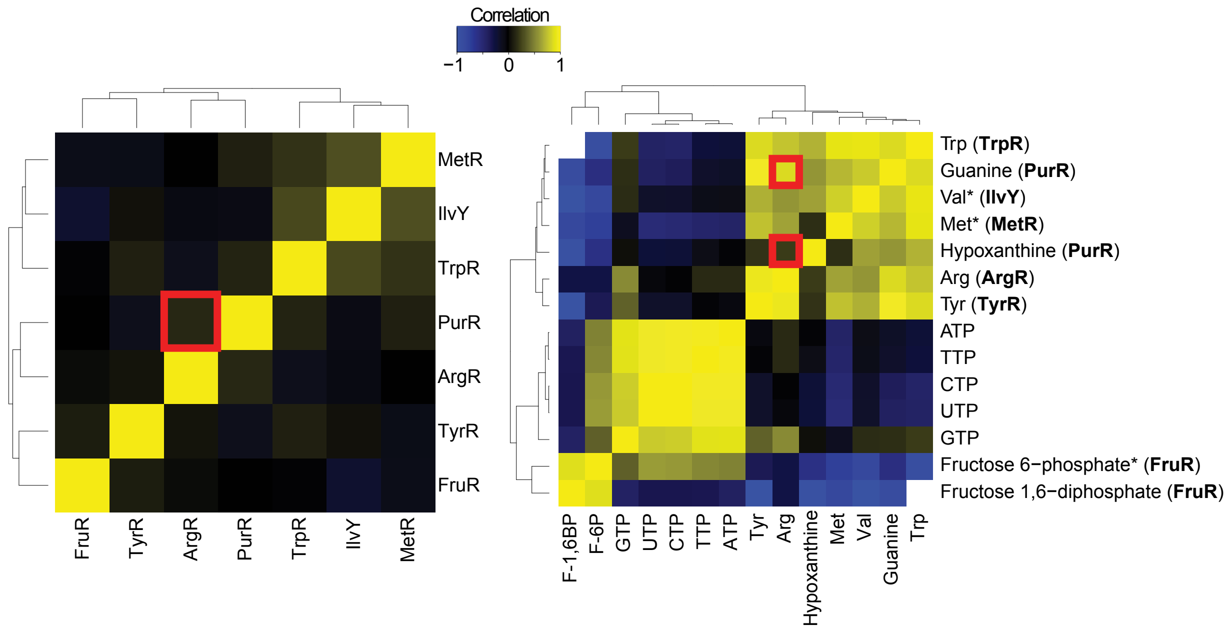

Figure S30: **Metabolite correlations may explain co-regulation within metabolically-linked corems.** (Left) Expression correlation for TFs associated with three corems described in the text (ec516031,ec512157,ec516034). (Right) Correlation allosteric regulators for these TFs. TF regulated by each biomolecule listed in parentheses [46]. Red boxes indicate PurR-ArgR and their corresponding effector molecules. Data from [28].

References

[1] Y.-Y. Ahn, J. P. Bagrow, and S. Lehmann. Link communities reveal multiscale complexity in networks. *Nature*, 466(7307):761–764, Aug 2010.

[2] E. J. Alm, K. H. Huang, M. N. Price, R. P. Koche, K. Keller, I. L. Dubchak, and A. P. Arkin. The microbesonline web site for comparative genomics. *Genome Res*, 15(7):1015–1022, Jul 2005.

[3] T. L. Bailey and M. Gribskov. Methods and statistics for combining motif match scores. *J Comput Biol*, 5(2):211–221, 1998.

[4] N. Baliga, M. Pan, Y. Goo, E. Yi, D. Goodlett, K. Dimitrov, P. Shannon, R. Aebersold, W. Ng, and L. Hood. Coordinate regulation of energy transduction modules in halobacterium sp. analyzed by a global systems approach. *Proc Natl Acad Sci USA*, 99(23):14913–14918, 2002.

[5] N. S. Baliga, S. J. Bjork, R. Bonneau, M. Pan, C. Iloanusi, M. C. H. Kottemann, L. Hood, and J. DiRuggiero. Systems level insights into the stress response to uv radiation in the halophilic archaeon halobacterium nrc-1. *Genome Res*, 14(6):1025–1035, Jun 2004.

[6] N. S. Baliga, S. P. Kennedy, W. V. Ng, L. Hood, and S. DasSarma. Genomic and genetic dissection of an archaeal regulon. *Proc Natl Acad Sci U S A*, 98(5):2521–2525, Feb 2001.

[7] T. Barrett, D. B. Troup, S. E. Wilhite, P. Ledoux, D. Rudnev, C. Evangelista, I. F. Kim, A. Soboleva, M. Tomashevsky, and R. Edgar. Ncbi geo: mining tens of millions of expression profiles–database and tools update. *Nucleic Acids Res*, 35(Database issue):D760–D765, Jan 2007.

[8] R. Bonneau, N. S. Baliga, E. W. Deutsch, P. Shannon, and L. Hood. Comprehensive de novo structure prediction in a systems-biology context for the archaea halobacterium sp. nrc-1. *Genome Biol*, 5(8):R52, 2004.

[9] R. Bonneau, M. T. Facciotti, D. J. Reiss, A. K. Schmid, M. Pan, A. Kaur, V. Thorsson, P. Shannon, M. H. Johnson, J. C. Bare, W. Longabaugh, M. Vuthoori, K. Whitehead, A. Madar, L. Suzuki, T. Mori, D.-E. Chang, J. Diruggiero, C. H. Johnson, L. Hood, and N. S. Baliga. A predictive model for transcriptional control of physiology in a free living cell. *Cell*, 131(7):1354–1365, Dec 2007.

[10] R. Bonneau, D. J. Reiss, P. Shannon, M. Facciotti, L. Hood, N. S. Baliga, and V. Thorsson. The inferelator: an algorithm for learning parsimonious regulatory networks from systems-biology data sets de novo. *Genome Biol*, 7(5):R36, 2006.

[11] L. Breiman. Bagging predictors. In *Machine Learning*, pages 123–140, 1996.

[12] L. Breiman. Random forests. *Machine Learning*, 45(1):5–32, 2001.

[13] P. Buhlmann and B. Yu. Analyzing bagging. *Annals of Statistics*, (30):927–961, 2002.

[14] B.-K. Cho, S. Federowicz, Y.-S. Park, K. Zengler, and B. . Palsson. Deciphering the transcriptional regulatory logic of amino acid metabolism. *Nat Chem Biol*, 8(1):65–71, Jan 2012.

[15] B.-K. Cho, S. A. Federowicz, M. Embree, Y.-S. Park, D. Kim, and B. . Palsson. The purr regulon in escherichia coli k-12 mg1655. *Nucleic Acids Res*, 39(15):6456–6464, Aug 2011.

[16] R. Day and A. Lisovich. DAVIDQuery: retrieval from the DAVID bioinformatics data resource into r, 2010.

[17] J. Day-Richter, M. A. Harris, M. Haendel, Gene Ontology OBO-Edit Working Group, and S. Lewis. OBO-Edit–an ontology editor for biologists. *Bioinformatics (Oxford, England)*, 23(16):2198–2200, Aug. 2007. PMID: 17545183.

[18] J. Demeter, C. Beauheim, J. Gollub, T. Hernandez-Boussard, H. Jin, D. Maier, J. C. Matese, M. Nitzberg, F. Wymore, Z. K. Zachariah, P. O. Brown, G. Sherlock, and C. A. Ball. The stanford microarray database: implementation of new analysis tools and open source release of software. *Nucleic Acids Res*, 35(Database issue):D766–D770, Jan 2007.

[19] J. Dennis, Glynn, B. T. Sherman, D. A. Hosack, J. Yang, W. Gao, H. C. Lane, and R. A. Lempicki. DAVID: database for annotation, visualization, and integrated discovery. *Genome biology*, 4(5):P3, 2003. PMID: 12734009 PMCID: PMC3720094.

[20] M. T. Facciotti, W. L. Pang, F.-y. Lo, K. Whitehead, T. Koide, K.-i. Masumura, M. Pan, A. Kaur, D. J. Larsen, D. J. Reiss, L. Hoang, E. Kalisiak, T. Northen, S. A. Trauger, G. Siuzdak, and N. S. Baliga. Large scale physiological readjustment during growth enables rapid, comprehensive and inexpensive systems analysis. *BMC Syst Biol*, 4:64, 2010.

[21] M. T. Facciotti, D. J. Reiss, M. Pan, A. Kaur, M. Vuthoori, R. Bonneau, P. Shannon, A. Srivastava, S. M. Donohoe, L. E. Hood, and N. S. Baliga. General transcription factor specified global gene regulation in archaea. *Proceedings of the National Academy of Sciences of the United States of America*, 104(11):4630–4635, Mar. 2007. WOS:000244972700069.

[22] M. T. Facciotti, D. J. Reiss, M. Pan, A. Kaur, M. Vuthoori, R. Bonneau, P. Shannon, A. Srivastava, S. M. Donohoe, L. E. Hood, and N. S. Baliga. General transcription factor specified global gene regulation in archaea. *Proc Natl Acad Sci U S A*, 104(11):4630–4635, Mar 2007.

[23] J. H. Friedman, T. Hastie, and R. Tibshirani. Regularization paths for generalized linear models via coordinate descent. *Journal of Statistical Software*, 33(1):1–22, 2 2010.

[24] S. Gama-Castro, H. Salgado, M. Peralta-Gil, A. Santos-Zavaleta, L. Muiz-Rascado, H. Solano-Lira, V. Jimenez-Jacinto, V. Weiss, J. S. Garca-Sotelo, A. Lpez-Fuentes, L. Porrn-Sotelo, S. Alquicira-Hernndez, A. Medina-Rivera, I. Martnez-Flores, K. Alquicira-Hernndez, R. Martnez-Adame, C. Bonavides-Martnez, J. Miranda-Ros, A. M. Huerta, A. Mendoza-Vargas, L. Collado-Torres, B. Taboada, L. Vega-Alvarado, M. Olvera, L. Olvera, R. Grande, E. Morett, and J. Collado-Vides. Regulondb version 7.0: transcriptional regulation of escherichia coli k-12 integrated within genetic sensory response units (gensor units). *Nucleic Acids Res*, 39(Database issue):D98–105, Jan 2011.

[25] S. Gupta, J. A. Stamatoyannopoulos, T. L. Bailey, and W. S. Noble. Quantifying similarity between motifs. *Genome Biol*, 8(2):R24, 2007.

[26] T. Ideker, V. Thorsson, A. F. Siegel, and L. E. Hood. Testing for differentially-expressed genes by maximum-likelihood analysis of microarray data. *J Comput Biol*, 7(6):805–817, 2000.

[27] R. Ihaka and R. Gentleman. R: A language for data analysis and graphics. *Journal of Computational and Graphical Statistics*, 5(3):299–314, 1996.

[28] N. Ishii, K. Nakahigashi, T. Baba, M. Robert, T. Soga, A. Kanai, T. Hirasawa, M. Naba, K. Hirai, A. Hoque, P. Y. Ho, Y. Kakazu, K. Sugawara, S. Igarashi, S. Harada, T. Masuda, N. Sugiyama, T. Togashi, M. Hasegawa, Y. Takai, K. Yugi, K. Arakawa, N. Iwata, Y. Toya, Y. Nakayama, T. Nishioka, K. Shimizu, H. Mori, and M. Tomita. Multiple high-throughput analyses monitor the response of e. coli to perturbations. *Science*, 316(5824):593–597, Apr. 2007. PMID: 17379776.

[29] A. Joshi, R. De Smet, K. Marchal, Y. Van de Peer, and T. Michoel. Module networks revisited: computational assessment and prioritization of model predictions. *Bioinformatics*, 25(4):490–496, Feb 2009.

[30] A. T. Kalinka and P. Tomancak. linkcomm: an r package for the generation, visualization, and analysis of link communities in networks of arbitrary size and type. *Bioinformatics*, 27(14):2011–2012, Jul 2011.

[31] A. Kaur, M. Pan, M. Meislin, M. Facciotti, R. El-Geweley, and N. Baliga. Survival strategies of an archaeal organism to withstand stress from transition metals. *Genome Research*, 2006.

[32] A. Kaur, P. T. Van, C. R. Busch, C. K. Robinson, M. Pan, W. L. Pang, D. J. Reiss, J. DiRuggiero, and N. S. Baliga. Coordination of frontline defense mechanisms under severe oxidative stress. *Molecular Systems Biology*, 6:393, July 2010. WOS:000284524200005.

[33] A. B. Khodursky, J. A. Bernstein, B. J. Peter, V. Rhodius, V. F. Wendisch, and D. P. Zimmer. Escherichia coli spotted double-strand DNA microarrays. In M. J. Brownstein and A. B. Khodursky, editors, *Functional Genomics*, number 224 in Methods in Molecular Biology, pages 61–78. Humana Press, Jan. 2003.

[34] D. Kixmller, H. Strahl, A. Wende, and J.-C. Greie. Archaeal transcriptional regulation of the prokaryotic kdpfabc complex mediating k(+) uptake in h. salinarum. *Extremophiles*, 15(6):643–652, Nov 2011.

[35] T. Koide, D. J. Reiss, J. C. Bare, W. L. Pang, M. T. Facciotti, A. K. Schmid, M. Pan, B. Marzolf, P. T. Van, F.-Y. Lo, A. Pratap, E. W. Deutsch, A. Peterson, D. Martin, and N. S. Baliga. Prevalence of transcription promoters within archaeal operons and coding sequences. *Mol Syst Biol*, 5:285, 2009.

[36] A. Krogh and P. Sollich. Statistical mechanics of ensemble learning. *Physical Review E*, 55(1):811–825, 1997.

[37] N. Kurbatova, T. Adamusiak, P. Kurnosov, M. A. Swertz, and M. Kapushesky. ontoCAT: an r package for ontology traversal and search. *Bioinformatics (Oxford, England)*, 27(17):2468–2470, Sept. 2011. PMID: 21697126.

[38] P. Langfelder and S. Horvath. Wgcna: an r package for weighted correlation network analysis. *BMC Bioinformatics*, 9:559, 2008.

[39] K. Lemmens, T. De Bie, T. Dhollander, S. C. De Keersmaecker, I. M. Thijs, G. Schoofs, A. De Weerdt, B. De Moor, J. Vanderleyden, J. Collado-Vides, K. Engelen, and K. Marchal. Distiller: a data integration framework to reveal condition dependency of complex regulons in escherichia coli. *Genome Biol*, 10(3):R27, 2009.

[40] K. Lemmens, T. De Bie, T. Dhollander, P. Monsieurs, B. De Moor, J. Collado-Vides, K. Engelen, and K. Marchal. The condition-dependent transcriptional network in escherichia coli. *Ann N Y Acad Sci*, 1158:29–35, Mar 2009.

[41] D. Marbach, J. C. Costello, R. Kffner, N. M. Vega, R. J. Prill, D. M. Camacho, K. R. Allison, D. R. E. A. M. C. , M. Kellis, J. J. Collins, and G. Stolovitzky. Wisdom of crowds for robust gene network inference. *Nat Methods*, 9(8):796–804, Aug 2012.

[42] B. Marzolf, E. W. Deutsch, P. Moss, D. Campbell, M. H. Johnson, and T. Galitski. Sbeams-microarray: database software supporting genomic expression analyses for systems biology. *BMC Bioinformatics*, 7:286, 2006.

[43] T. Michoel, R. De Smet, A. Joshi, Y. Van de Peer, and K. Marchal. Comparative analysis of module-based versus direct methods for reverse-engineering transcriptional regulatory networks. *BMC Syst Biol*, 3:49, 2009.

[44] R. J. Nichols, S. Sen, Y. J. Choo, P. Beltrao, M. Zietek, R. Chaba, S. Lee, K. M. Kazmierczak, K. J. Lee, A. Wong, M. Shales, S. Lovett, M. E. Winkler, N. J. Krogan, A. Typas, and C. A. Gross. Phenotypic landscape of a bacterial cell. *Cell*, 144(1):143–156, Jan. 2011. PMID: 21185072 PMCID: PMC3060659.

[45] P. S. Novichkov, A. E. Kazakov, D. A. Ravcheev, S. A. Leyn, G. Y. Kovaleva, R. A. Sutormin, M. D. Kazanov, W. Riehl, A. P. Arkin, I. Dubchak, and D. A. Rodionov. Regprecise 3.0 - a resource for genome-scale exploration of transcriptional regulation in bacteria. *BMC Genomics*, 14:745, 2013.

[46] P. S. Novichkov, O. N. Laikova, E. S. Novichkova, M. S. Gelfand, A. P. Arkin, I. Dubchak, and D. A. Rodionov. Regprecise: a database of curated genomic inferences of transcriptional regulatory interactions in prokaryotes. *Nucleic Acids Res*, 38(Database issue):D111–D118, Jan 2010.

[47] H. Parkinson, M. Kapushesky, M. Shojatalab, N. Abeygunawardena, R. Coulson, A. Farne, E. Holloway, N. Kolesnykov, P. Lilja, M. Lukk, R. Mani, T. Rayner, A. Sharma, E. William, U. Sarkans, and A. Brazma. Arrayexpress–a public database of microarray experiments and gene expression profiles. *Nucleic Acids Res*, 35(Database issue):D747–D750, Jan 2007.

[48] M. N. Price, K. H. Huang, E. J. Alm, and A. P. Arkin. A novel method for accurate operon predictions in all sequenced prokaryotes. *Nucleic Acids Res*, 33(3):880–892, 2005.

[49] M. N. Price, K. H. Huang, A. P. Arkin, and E. J. Alm. Operon formation is driven by co-regulation and not by horizontal gene transfer. *Genome Res*, 15(6):809–819, Jun 2005.

[50] D. J. Reiss, N. S. Baliga, and R. Bonneau. Integrated biclustering of heterogeneous genome-wide datasets for the inference of global regulatory networks. *BMC Bioinformatics*, 7:280, 2006.

[51] D. J. Reiss, M. T. Facciotti, and N. S. Baliga. Model-based deconvolution of genome-wide dna binding. *Bioinformatics*, 24(3):396–403, Feb 2008.

[52] A. K. Schmid, M. Pan, K. Sharma, and N. S. Baliga. Two transcription factors are necessary for iron homeostasis in a salt-dwelling archaeon. *Nucleic Acids Res*, 39(7):2519–2533, Apr 2011.

[53] A. K. Schmid, D. J. Reiss, A. Kaur, M. Pan, N. King, P. T. Van, L. Hohmann, D. B. Martin, and N. S. Baliga. The anatomy of microbial cell state transitions in response to oxygen. *Genome Res*, 17(10):1399–1413, Oct 2007.

[54] A. K. Schmid, D. J. Reiss, M. Pan, T. Koide, and N. S. Baliga. A single transcription factor regulates evolutionarily diverse but functionally linked metabolic pathways in response to nutrient availability. *Mol Syst Biol*, 5:282, 2009.

[55] M. A. Serrano, M. Bogu, and A. Vespignani. Extracting the multiscale backbone of complex weighted networks. *Proc Natl Acad Sci U S A*, 106(16):6483–6488, Apr 2009.

[56] P. Shannon, A. Markiel, O. Ozier, N. S. Baliga, J. T. Wang, D. Ramage, N. Amin, B. Schwikowski, and T. Ideker. Cytoscape: a software environment for integrated models of biomolecular interaction networks. *Genome Res*, 13(11):2498–2504, Nov 2003.

[57] P. T. Shannon, D. J. Reiss, R. Bonneau, and N. S. Baliga. The gaggle: an open-source software system for integrating bioinformatics software and data sources. *BMC Bioinformatics*, 7:176, 2006.

[58] D. Szklarczyk, A. Franceschini, M. Kuhn, M. Simonovic, A. Roth, P. Minguez, T. Doerks, M. Stark, J. Muller, P. Bork, L. J. Jensen, and C. von Mering. The string database in 2011: functional interaction networks of proteins, globally integrated and scored. *Nucleic Acids Res*, 39(Database issue):D561–D568, Jan 2011.

[59] S. van Dongen and C. Abreu-Goodger. Using mcl to extract clusters from networks. *Methods Mol Biol*, 804:281–295, 2012.

[60] J. van Helden, B. André, and J. Collado-Vides. A web site for the computational analysis of yeast regulatory sequences. *Yeast*, 16(2):177–187, Jan 2000.

[61] D. J. Watts and S. H. Strogatz. Collective dynamics of 'small-world' networks. *Nature*, 393(6684):440–442, Jun 1998.

[62] K. Whitehead, A. Kish, M. Pan, A. Kaur, D. J. Reiss, N. King, L. Hohmann, J. DiRuggiero, and N. S. Baliga. An integrated systems approach for understanding cellular responses to gamma radiation. *Mol Syst Biol*, 2:47, 2006.

[63] K. Whitehead, M. Pan, K.-i. Masumura, R. Bonneau, and N. S. Baliga. Diurnally entrained anticipatory behavior in archaea. *Plos One*, 4(5):e5485, May 2009. WOS:000265933800015.

[64] S. H. Yoon, D. J. Reiss, J. C. Bare, D. Tenenbaum, M. Pan, J. Slagel, R. L. Moritz, S. Lim, M. Hackett, A. L. Menon, M. W. W. Adams, A. Barnebey, S. M. Yannone, J. A. Leigh, and N. S. Baliga. Parallel evolution of transcriptome architecture during genome reorganization. *Genome Res*, 21(11):1892–1904, Nov 2011.

[65] S. H. Yoon, S. Turkarslan, D. J. Reiss, M. Pan, J. A. Burn, K. C. Costa, T. J. Lie, J. Slagel, R. L. Moritz, M. Hackett, J. A. Leigh, and N. S. Baliga. A systems level predictive model for global gene regulation of methanogenesis in a hydrogenotrophic methanogen. *Genome Res*, 23(11):1839–1851, Nov 2013.

[66] J. Zhou and K. E. Rudd. Ecogene 3.0. *Nucleic Acids Res*, 41(Database issue):D613–D624, Jan 2013.

[67] H. Zou and T. Hastie. Regularization and variable selection via the elastic net. *Journal of the Royal Statistical Society, Series B*, 67:301–320, 2005.
